# Silver Organometallics that are Highly Potent Thioredoxin and Glutathione Reductase Inhibitors: Exploring the Correlations of Solution Chemistry with the Strong Antibacterial Effects

**DOI:** 10.1021/acsinfecdis.4c00104

**Published:** 2024-04-12

**Authors:** Igor V. Esarev, Bianka Karge, Haoxuan Zeng, Petra Lippmann, Peter G. Jones, Hedda Schrey, Mark Brönstrup, Ingo Ott

**Affiliations:** † Institute of Medicinal and Pharmaceutical Chemistry, Technische Universität Braunschweig, Beethovenstraße 55, 38106 Braunschweig, Germany; ‡ Department of Chemical Biology, Helmholtz Centre for Infection Research, Inhoffenstraße 7, 38124 Braunschweig, Germany; § Department of Microbial Drugs, Helmholtz Centre for Infection Research GmbH and German Centre for Infection Research (DZIF), Partner Site Hannover/Braunschweig, Inhoffenstraße 7, 38124 Braunschweig, Germany; ∥ Institute of Microbiology, Technische Universität Braunschweig, Spielmannstraße 7, 38106 Braunschweig, Germany; ⊥ Institute of Inorganic and Analytical Chemistry, Technische Universität Braunschweig, Hagenring 30, 38106 Braunschweig, Germany

**Keywords:** antibacterial, bioorganometallics, N-heterocyclic carbene, silver

## Abstract

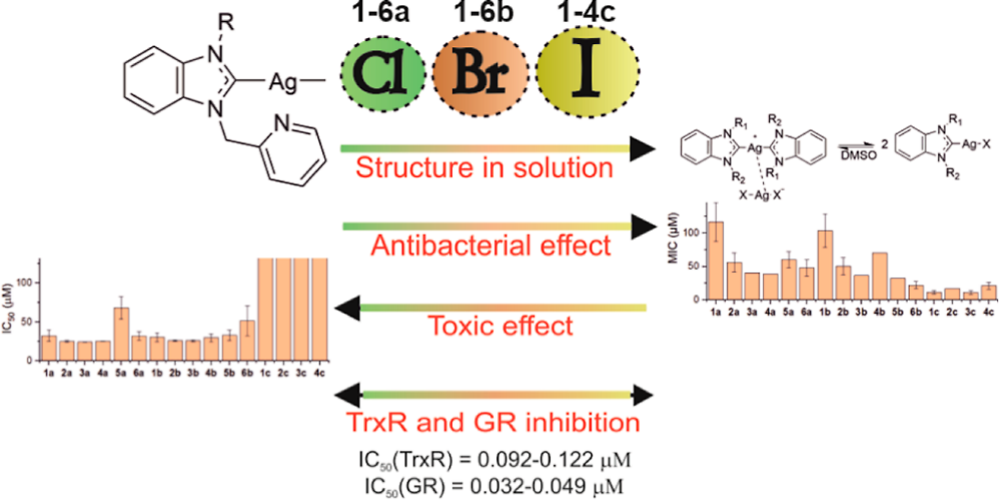

The antibacterial activity of silver species is well-established; however, their mechanism of action has not been adequately explored. Furthermore, issues of low-molecular silver compounds with cytotoxicity, stability, and solubility hamper their progress to drug leads. We have investigated silver N-heterocyclic carbene (NHC) halido complexes [(NHC)AgX, X = Cl, Br, and I] as a promising new type of antibacterial silver organometallics. Spectroscopic studies and conductometry established a higher stability for the complexes with iodide ligands, and nephelometry indicated that the complexes could be administered in solutions with physiological chloride levels. The complexes showed a broad spectrum of strong activity against pathogenic Gram-negative bacteria. However, there was no significant activity against Gram-positive strains. Further studies clarified that tryptone and yeast extract, as components of the culture media, were responsible for this lack of activity. The reduction of biofilm formation and a strong inhibition of both glutathione and thioredoxin reductases with IC_50_ values in the nanomolar range were confirmed for selected compounds. In addition to their improved physicochemical properties, the compounds with iodide ligands did not display cytotoxic effects, unlike the other silver complexes. In summary, silver NHC complexes with iodide secondary ligands represent a useful scaffold for nontoxic silver organometallics with improved physicochemical properties and a distinct mechanism of action that is based on inhibition of thioredoxin and glutathione reductases.

The development and drug discovery of the first antibiotics, such as the penicillins, almost a century ago represent one of the biggest achievements in medicinal chemistry.^1^ However, over the decades antimicrobial resistance has emerged and is nowadays considered as a leading cause of death worldwide that cannot be lowered with the existing pool of antibacterial drugs.^2^ This leads to a high demand for the development of effective antibiotics that do not belong to the existing compound classes,^3^ for which resistance phenomena are becoming more and more common.

Silver and its compounds represent an ancient antimicrobial treatment that may help supply the need for effective antibiotics in modern times.^4−8^ Silver species have many applications in consumer products and in medicine and cosmetics because of their antimicrobial effects. Silver nitrate and silver sulfadiazine (SSD), the silver complex of a sulfonamide antibiotic, are currently applied as topical anti-infectives. At low dosages, silver is efficiently removed from the body and does not trigger severe toxic effects. However, toxicity upon acute or chronic overexposure limits the potential for systemic use.^9−12^ Silver probably triggers its biological effects by a multitarget mechanism that is not fully understood at present.^12−14^ Relevant mechanisms include effects on the cell wall, interactions with DNA, binding or inhibition of enzymes and membrane proteins, or the generation of reactive oxygen species (ROS).

A recent report by Sun and colleagues identified 38 silver binding proteins for silver antimicrobials in *Staphylococcus aureus* and concluded that the multitarget mode of action endows silver with its sustainable antimicrobial efficacy.^15^ The same group also reported the restoration of colistin efficacy by silver ions, thus highlighting the potential of silver species to combat bacterial resistance mechanisms.^16^

As the biological effects of silver are attributed to the release of Ag^+^ ions, the identification of robustly coordinated ligands is crucial for silver metallodrug development, in order to enhance bioavailability, improve targeting, and optimize binding to molecular drug targets. N-heterocyclic carbenes (NHCs) are ligands that form highly stable organometallic complexes with many transition metals and are therefore nowadays widely employed in bioinorganic medicinal chemistry.^17,18^ Youngs and co-workers have pioneered the field of antibacterial silver NHC complexes and confirmed the antibacterial efficacy of this type of complexes in vitro and in vivo (see Figure 1 for **1** as a representative example).^19−23^ The rapidly increasing number of reported silver NHC complexes with antibacterial activity confirms the recognized potential of such complexes for drug discovery.^24−29^ For example, complex **2**, developed by Tacke and colleagues, represents a promising example of a silver drug candidate, for which detailed proteomic responses have been recorded, and the inhibition of bacterial thioredoxin reductase was demonstrated.^21,30−32^ Notably, silver NHC complexes have also been described as antiviral and cytotoxic agents.^33−36^

**Figure 1:**
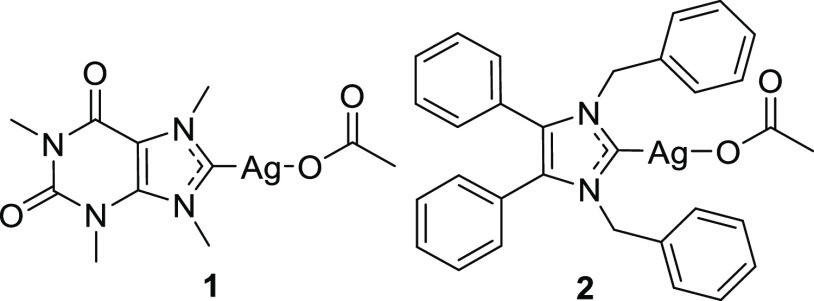
Examples of antibacterial silver NHC complexes.

Despite such growing interest, limited information on the physicochemical properties of silver NHC complexes and on their speciation under physiological conditions restricts further development of this type of metal-based drug candidates. Moreover, cytotoxic effects have to be carefully considered when assessing their potential use as safe antibiotics. Here, we report our studies addressing these critical issues with the aim of providing the basis for validating antibacterial silver NHC complexes as drug leads.

## Results and Discussion

### Chemistry

**Scheme 1:**
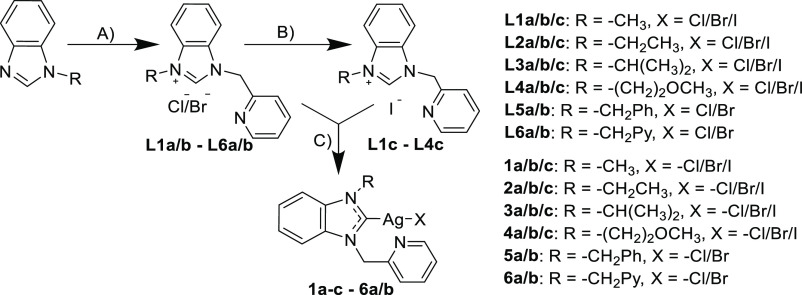
Synthesis of Ag(I) NHC Complexes (A) PicX, CH_3_CN, reflux, 48 h/BnX, toluene, reflux, 12 h; (B) KI, acetone/MeOH mixture (9:1), r.t., 48 h; and (C) 0.6 equiv Ag_2_O, dry CH_2_Cl_2_, 3 Å sieves (max. 10 wt %) r.t., 3–6 h, darkness

As a NHC core structure, a benzimidazole-based ligand with a pyridine-containing side chain was chosen, for which stable coordination of silver could be expected. In the first step of the synthesis procedure, the nonsymmetrical benzimidazolium cations (**L1a–L6b**) with chloride or bromide counterions were obtained by the reaction of the 1-substituted benzimidazoles with the respective picol-2-yl or benzyl halides (Scheme 1). The iodide analogues **L1c–L4c** were synthesized via ion exchange reaction using potassium iodide. All pro-ligands were fully characterized via elemental analysis, NMR spectroscopy, and ESI mass spectrometry.

The target silver NHC complexes **1a/c**–**6a/b** were synthesized according to the established procedure described by Wang and Lin.^37^ The reaction was carried out in dry dichloromethane over 3 Å sieves, which accelerate complex formation by adsorption of water formed during the process.^38^ The formation of the chloride (**1a**–**6a**) and bromide (**1b**–**6b**) analogues was completed in 6 h with moderate to good yields (60–82%). It is worth noting that, unlike the chloride and bromide analogues, the formation of a gray insoluble precipitate was observed during the synthesis of iodide complexes **1c**–**4c**, leading to reduced yields (42–45%). In addition, all attempts to isolate iodide analogues of **5a/b** and **6a/b** with aromatic rings on the second side chain were unsuccessful because of poor solubility of the resulting products in organic solvents.

To determine the composition of the abovementioned precipitate, the complexation reaction with **L1c–L4c** was performed using 0.45 equiv Ag_2_O to exclude the presence of residual silver oxide. According to the results of elemental analyses, the resulting composition formally corresponded to the formula [Ag(NHC)I]·AgI (see Table S1). Furthermore, it was noticed that the precipitation intensified with increasing the amount of Ag_2_O and that the amount of insoluble solid in the mixture was very small for complexes with larger side chains (i.e., complexes **3c** and **4c**). Taken together, these observations are in good agreement with the proposed formation of insoluble AgI adducts and the lower yields of the iodide NHC complexes.

Elemental analysis revealed that the composition of the isolated solids of **1a**–**6b** matched the expected (NHC)AgX structures (maximum deviation of the theoretical values: 0.5%). Analysis by ESI-MS showed the formation of biscarbene [(NHC)_2_Ag]^+^ fragments in the positive and AgX_2_^–^ ions in the negative mode, as is common for silver NHC complexes (see the Supporting Information).^19^ A characteristic feature of the ^1^H NMR spectra is the disappearance of the NC*H*N signals at ∼11 ppm for **1a**–**6b** upon formation of the metal–carbon bonds. Interestingly, for the CH_2_–Py protons, two singlet signals instead of one are observed for **4a**–**4c** and, to a lower extent, for several other complexes (see ^1^H NMR spectra in the Supporting Information). Such an effect was reported previously and interpreted as a manifestation of the equilibrium between mono- and biscarbene forms of silver NHC complexes (see below).^39−41^^13^C NMR spectroscopy of **1a**–**6b** in DMSO showed slight differences in the shift of the C2 signals with a deshielding effect correlating with increasing halide size (e.g., 189.33 ppm for **4a**, 190.38 ppm for **4b**, and 191.32 ppm for **4c**).

**Scheme 2:**
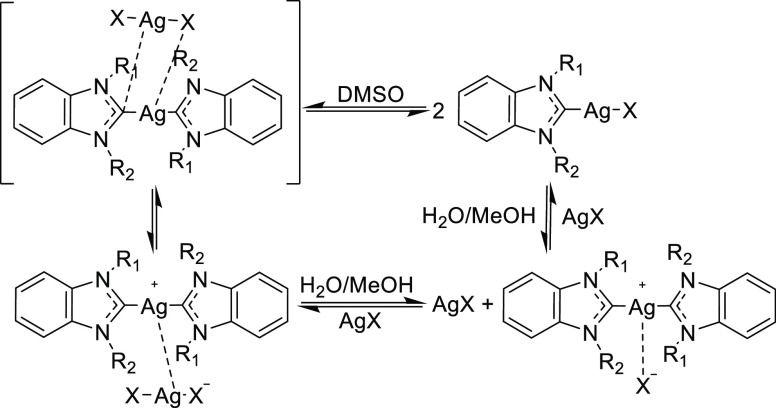
Proposed Fluxional Behavior of Silver NHC Complexes

One of the most remarkable features of silver NHC complexes is their structural flexibility. The generally dominant monocarbene [(NHC)AgX] form exists in solution in a dynamic equilibrium with the biscarbene [(NHC)_2_Ag]^+^[AgX_2_]^−^ form (see Scheme 2).^37,42^ Depending on several factors, such as lipophilicity and bulkiness of the ligands, the strength of the Ag–C2 bond, and the polarity of the solvent used, neutral monocarbene or charged biscarbene complexes can be formed and isolated.^39,40,43,44^ Clearly, such behavior may greatly affect the silver ion release rate and consequently the bioactivity of silver NHC complexes. To study the equilibrium, various analytical techniques have been previously utilized. In particular, ^13^C and ^1^H NMR 2D EXSY experiments were performed to determine the bonding strength and exchange rate in silver complexes.^39,41,43^ However, the reports are almost exclusively dedicated to complexes with chloride as the halide ligand.

**Figure 2:**
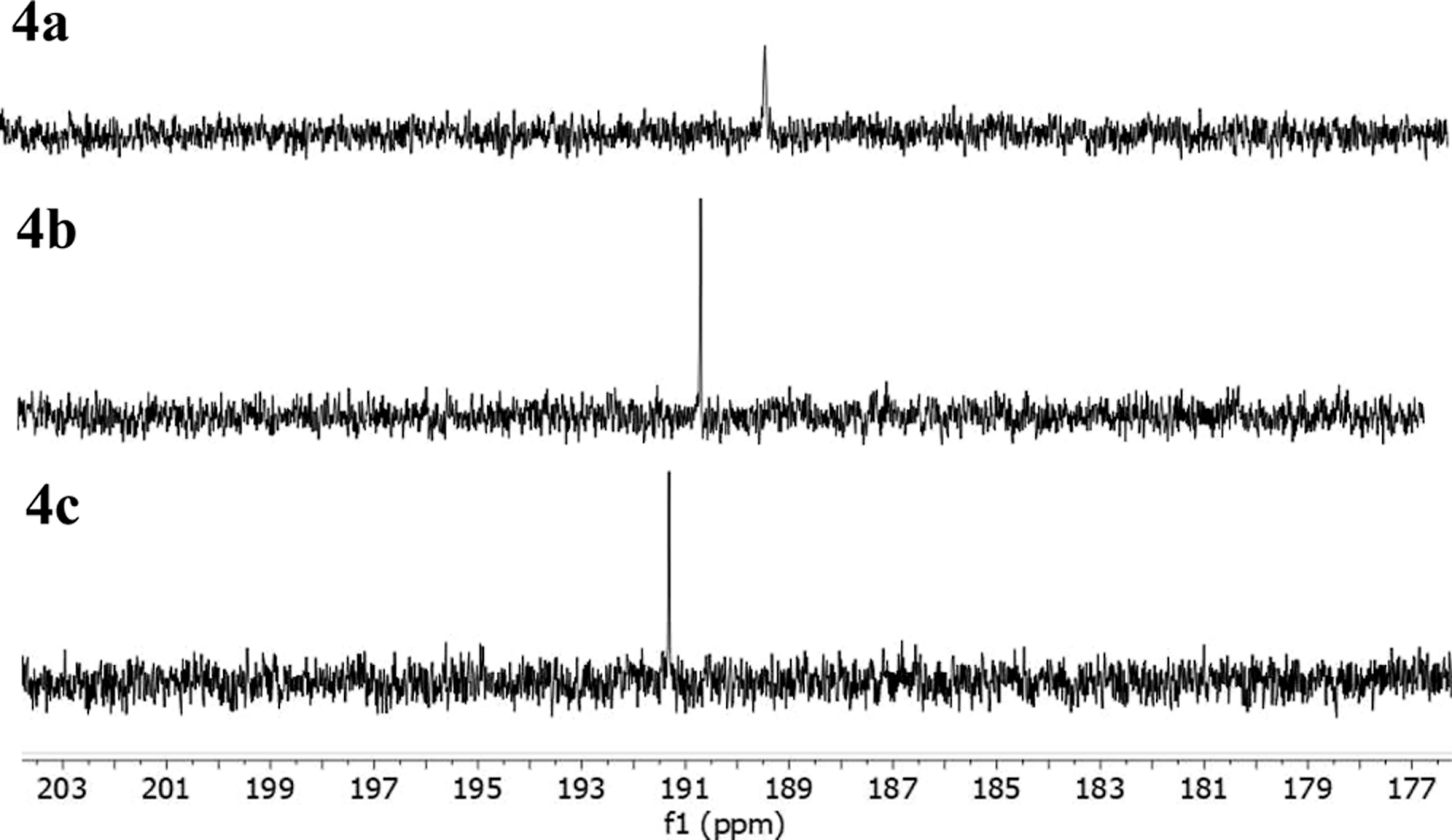
^13^C NMR (DMSO, 126 MHz) of silver complexes **4a** (top), **4b** (middle), and **4c** (bottom) in the region 203–177 ppm, showing the C2 carbon signals.

To reveal the influence of different halide ions on the monocarbene/biscarbene-equilibrium, ^13^C NMR spectra of selected compounds **4a**–**4c** in DMSO at 25 mM concentrations were recorded (see Figure 2). The C2 signals in the ^13^C NMR spectra appeared as sharp singlets for all complexes, which is considered as evidence of the dynamic equilibrium in solution.^19^ The abovementioned slight deshielding effect with the increase of halide size implies a decrease of the electron density on the carbene ligand and a possible increase of the electron donation toward the metal center.^43^ Such behavior may indicate the stabilization of one form in solution with the introduction of larger halides.

To detect the dynamic equilibrium between two forms of silver NHC complexes occurring in DMSO solution, EXSY experiments were performed with selected compounds **4a**–**c** (see Figures S1–S3). For **4a**, the spectra show clearly visible positive cross-peaks between the CH_2_–CH_2_ hydrogens of the methoxyethyl side chain and in the aromatic region. Such signals provide evidence of dynamic exchange processes between various structures of the complex. Interestingly, once bromide (**4b**) or iodide (**4c**) is introduced, the positive cross peaks can barely be observed, indicating the stabilization of one structural form of the complex upon increasing halide size.

Since both the neutral monocarbene and the ionic biscarbene form of the complexes may exist in various proportions in the solution, conductometry studies were performed to reveal the dominant structure. The molar conductivity of 1.0 mM solutions of each complex in DMSO was determined after different periods up to 24 h using AgNO_3_ as 1:1 electrolyte reference (see Table 1). According to the literature, the expected range of molar conductivity for 1:1 electrolytes in DMSO is 35–90 S cm^2^ mol^–1^.^45,46^ The resulting conductivities of all compounds used in this study do not exceed 10 S cm^2^ mol^–1^ (when calculated as the monocarbene form). This indicates that all complexes maintain the neutral monocarbene form in DMSO, with minor amounts of the charged biscarbene formed immediately after dissolution.

**Table 1:** Molar Conductivities (Λ_M_) of 1.0 mM Solutions of Silver Complexes in DMSO at Different Time after Dissolution Calculated as Monocarbenes (NHC)AgX)

compound	Λ_M_, S cm^2^ mol^–^^1^	
	1 h	24 h
AgNO_3_	37.8	38.1
**1a**	2.88	3.05
**1b**	4.75	4.82
**1c**	7.82	8.13
**2a**	3.47	3.03
**2b**	5.47	5.07
**2c**	6.98	6.28
**3a**	3.34	2.91
**3b**	5.25	4.71
**3c**	8.15	7.62
**4a**	3.38	3.01
**4b**	5.11	5.08
**4c**	7.18	8.83
**5a**	4.24	3.70
**5b**	6.58	5.68
**6a**	3.75	4.15
**6b**	6.13	6.50

Additionally, selected complexes **1a/b** and **4a**–**c** were used for conductivity studies in diluted solutions (Figures S4 and S5). Molar conductivities measured at different concentrations of the complexes indicate whether they act as strong (with moderate, almost linear increase of conductivity) or weak electrolytes (exponential increase of conductivity).^47,48^ A sharp, roughly two-fold increase of molar conductivity with the decrease of concentration for chloride (**1a**, **4a**), and to a smaller extent, for bromide (**1b**, **4b**) complexes was observed in the aprotic polar solvent DMSO. In contrast, only a slight increase was observed for the iodide complexes (**1c**, **4c**). Considering the results both of NMR and conductivity studies, it can be concluded that the monocarbene/biscarbene equilibrium shifts toward the monocarbene form upon increasing the halide size. It is interesting to note that these results contrast with the report of Su et al., where a more rapid exchange was observed for iodide complexes bearing imidazole-based NHC ligands.^39^ However, electronic factors and influences of the NHC ligand are probably the reason for such differences.

**Figure 3:**
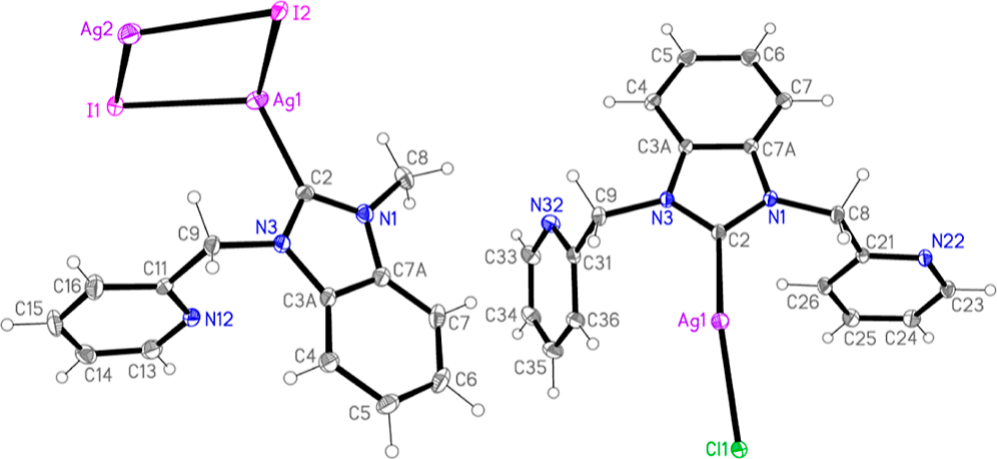
Asymmetric units of compounds 1c*AgI (left) and **6a** (right) in the crystal.

For the determination of structures of silver NHC halide complexes, the crystals of selected compounds **1c**, **6a**, and **6b** were grown by slow diffusion of Et_2_O into concentrated solutions of the respective compounds in CH_2_Cl_2_. Compound **1c** as crystallized has the unexpected stoichiometry (NHC)Ag_2_I_2_ (i.e., 1c*AgI, Figure 3), which is in line with results of the elemental analysis of the insoluble precipitates obtained upon preparation of the some of the (NHC)AgI species (see above). The extended structure of 1c*AgI is a tube polymer involving linked Ag_2_I_2_ quadrilaterals (Figure S6). The silver atom Ag1 is coordinated by the NHC carbon atom and by three iodine atoms (I1 and I2 within the asymmetric unit and I2′ via translation symmetry parallel to the *a*-axis). The silver atom I2 is coordinated by four iodine atoms (I1 and I2 within the asymmetric unit, I1′ via an inversion operator, and I2′ by translation parallel to the *a*-axis). The coordination geometry is distorted tetrahedral for both Ag atoms. The isotypic compounds **6a** and **6b** have the expected stoichiometry (NHC)AgX (X = Cl or Br) (Figures 3 and S7). The silver atom is coordinated by the NHC carbon and the chlorine atom of the asymmetric unit and by Cl′ (thus forming Ag_2_Cl_2_ quadrilaterals) and the pyridinic nitrogen atom N22′ of the neighboring unit, generated via different inversion centers. The resulting ribbon-shaped polymer runs parallel to the *a* axis (Figure S8). Interestingly, the coordination of nitrogen in the methylpyridine side chain to silver is not observed in the case of **1c** containing an asymmetric NHC ligand.

### Stability and Kinetic Solubility of Silver Complexes

**Figure 4:**
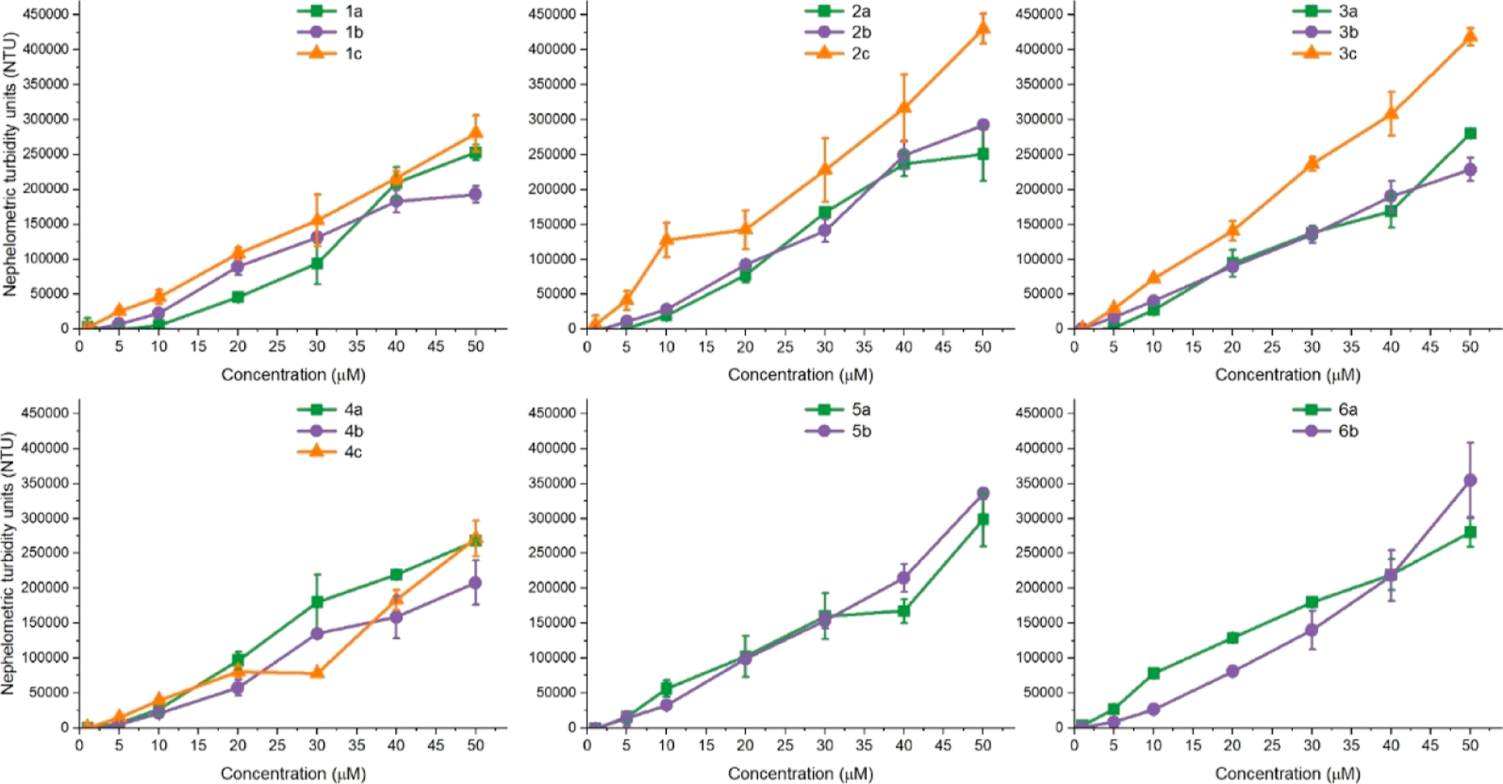
Solubility curves of silver NHC complexes in DMSO/PBS (0.2% v/v) mixture represented as increase of turbidity (NTU) with the increase of compound concentration.

Because of the formation of black colloidal silver in the course of degradation, the solution stability of silver NHC complexes can be monitored visually and with the help of nephelometry. All synthesized complexes were dissolved at 25 mM in DMSO and kept in the dark for 24 h. Visual inspection showed that the iodido complexes **3c** and **4c** were the only ones that did not show clear signs of degradation within the course of the experiment (Figure S9). Among the Cl and Br analogues, the most stable complexes were **3a/b**, **4a/b**, and **6b**. All other complexes (**1a**–**c**, **2a**–**c**, **5a/b**, and **6a)** turned out to be rather unstable, as evidenced by precipitation and silver colloid formation. To determine whether the complexes can be dissolved in physiological media at micromolar concentrations, nephelometric measurements in phosphate-buffered saline (PBS) were performed. The results shown in Figure 4 demonstrate the almost immediate precipitation of all complexes in PBS starting from concentrations of 5 μM. However, some conclusions on the influence of side chains and secondary ligands on solubility can be drawn.

(a) The increase of lipophilicity of the ligand side chains when going from **1a/b** to **4a/b** did not result in significant changes of the solubility. However, in the case of the bulky benzyl- or methylpyridine side chains of **5a/b** and **6a/b**, a slight increase of the nephelometric turbidity (NTU) values was observed (Figures 4 and S10).
(b) With the exception of **4c**, the iodido complexes had a lower solubility than the respective chlorido and bromido analogues.
(c) It is worth noting that silver ions can in principle precipitate in physiological media in form of AgCl, thus drastically affecting bioavailability and biological activity. However, the high linearity of the solubility curves, which is characteristic for kinetic solubility assays,^49,50^ shows that the NTU values increase in direct proportionality to the concentration (see Figure 4). In contrast, AgNO_3_, which has a high solubility in aqueous solutions, showed a more sigmoidal curve shape because of precipitation of AgCl (see Figure S11). Therefore, the high linearity of the functions in Figure 4 indicates that silver NHC complexes have a low tendency to release silver ions in PBS solution and remain as intact organometallics.

### Antibacterial Activity

The antibacterial effect of the silver NHC complexes and selected ligands was evaluated in two Gram-positive [*Enterococcus faecium*, methicillin resistant *S. aureus*, (MRSA)] and in four Gram-negative (*Acinetobacter baumannii*, *Escherichia coli*, *Klebsiella pneumoniae*, and *Pseudomonas aeruginosa*) pathogenic bacterial strains using broth microdilution assays. The resulting minimal inhibitory concentrations (MICs, Table 2) and EC_50_ (Table S6) were determined by a curve fitting procedure. Silver nitrate (AgNO_3_), SSD, and various antibiotics were used as references. The benzimidazolium salts **L3a**–**c** had no effect on the growth of all bacteria up to high concentrations (250 μM). In contrast, all silver compounds showed moderate to good activity against Gram-negative strains, especially against *A. baumannii* (MIC = 4–16 μM) and *K. pneumoniae* (MIC = 4–18 μM). Interestingly, the iodide complexes **3c** and **4c** generally triggered significantly better antibacterial activity against the Gram-negative bacteria in comparison to the respective chloride and bromide analogues. The same superiority of the iodide ligand over chloride and bromide was in principle observed with **1c** and **2c**; however, this trend was not fully consistent in all bacteria strains. In contrast to the promising results with the Gram-negative strains, a poor antibacterial activity against the two Gram-positive strains was determined for all silver complexes. Such observations are in line with previous reports, where Gram-positive bacteria have been shown to be less sensitive toward silver salts and their complexes.^11,26,51,52^

**Table 2:**
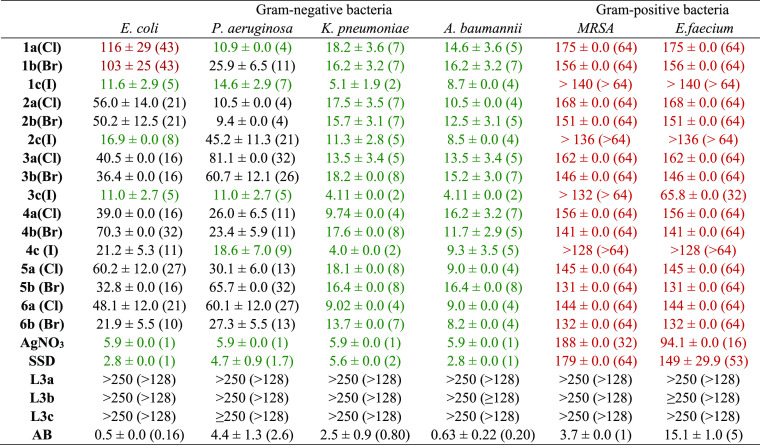
Mean Minimal Inhibitory Concentration (MIC) Values of Silver Complexes and References in μM ± Standard Error (μg/mL Values in Brackets) (*n* = 3)^a^

a MRSA = methicillin-resistant *S. aureus*; amikacin (*P. aeruginosa*), linezolid (*S. aureus*), and ciprofloxacin (*E. faecium*, *E. coli*, *A. baumannii*, and *K. pneumoniae*) were used as antibiotic references (**AB**). High activity with MIC values below 20 μM is marked in green, and low activity (MIC > 100 μM) is marked in red.

### Effect of Growth Media on the Antibacterial Activity

**Figure 5:**
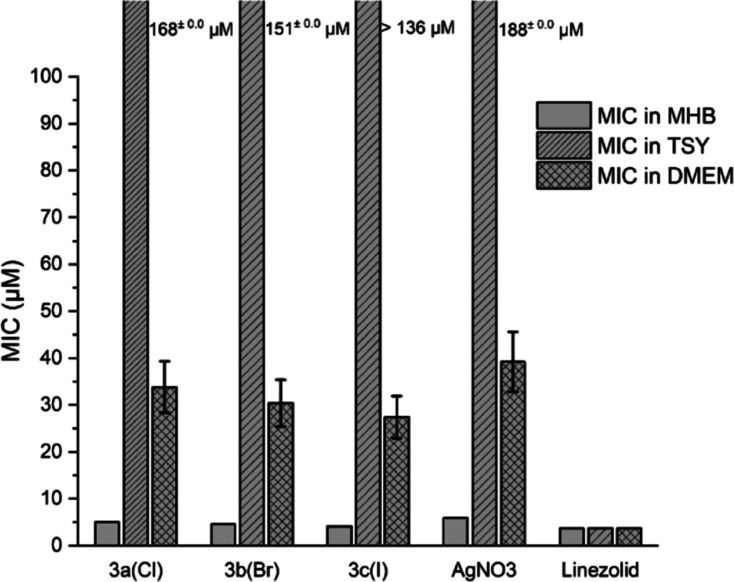
Antibacterial activity (MIC values) against MRSA of selected compounds **3a**–**c** in different media (*n* = 3).

The importance of the culture medium used in antibacterial assays has been pointed out for antibiotics and in particular for silver.^53−55^ In our recent studies, tryptic soy yeast (TSY) broth has been used for growing Gram-positive bacteria. Two components thereof, tryptone and yeast extract, have been reported to affect the antibacterial activity of silver nitrate.^55^ Moreover, such an effect was also observed for other media containing at least one of those components.^11,53^ Because of their favorable stability, complexes **3a**–**c** were selected to study the influence of various media on the antibacterial effects in a selected Gram-positive strain (MRSA). Silver nitrate served as a reference, and three different growth media were studied, namely, Müller-Hinton Broth (MHB, commonly used for Gram-negative bacteria), TSY broth (commonly used for Gram-positive bacteria), and DMEM supplemented with 10% FCS (commonly used for growth of mammalian and human cells). The results in Figure 5 clearly confirm the “deactivation” of the antibacterial effects of all silver species when performing the assay using the TSY broth. In contrast, when the experiments were performed with MHB or DMEM, all silver complexes showed appreciable antibacterial activity.

Analogous experiments in the Gram-negative *E. coli* further confirmed the negative effects of TSY broth in comparison to the routinely used MHB medium, although the decline in activity was less marked as with MRSA (Figure S12).

Further antibacterial studies were performed in order to clarify whether tryptone and yeast extract, as the major components of TSY, were responsible for the negative effect on antibacterial activity and would also prevent activity in experiments with the sensitive Gram-negative species. Therefore, experiments with *E. coli* using MHB with and without the addition of the two components were performed. Indeed, the antibacterial effect against *E. coli* was significantly lower when using MHB with added tryptone or yeast extract compared with MHB alone (see Table 3). More specifically, for the studied complexes **3a**–**c** in MHB with the additives, 3- to 5-fold higher MIC values were observed than without the additives.

**Table 3:** MIC (μM ± SE) for *E. coli* DSM 1116 Determined in Müller-Hinton Broth with the Addition of Tryptone (17 g/L) or Yeast Extract (3 g/L)

compounds	MHB	MHB + tryptone	MHB + yeast extract
**3a(Cl)**	40.5 ± 0.0	162.2 ± 0.0	162.2 ± 0.0
**3b(Br)**	36.4 ± 0.0	145.7 ± 0.0	121.5 ± 24.3
**3c(I)**	11.0 ± 2.7	54.9 ± 11.0	43.9 ± 11.0
AgNO_3_	5.89 ± 0.0	251.1 ± 62.8	123.6 ± 29.4
ciprofloxacin	0.47 ± 0.0	0.47 ± 0.0	0.47 ± 0.0

### Inhibition of Biofilm Formation

Silver and its complexes can strongly affect the growth of biofilms.^56−58^ In view of the promising activity against Gram-negative bacteria as mentioned above, we evaluated the inhibiting effect of the silver complexes on biofilm production by *P. aeruginosa* by a method based on crystal violet staining (Table 4 and Figure S14). Complexes **3a**–**c** and **4a**–**c** were selected for the test based on the results of solubility, stability, and antibacterial screening. With the exception of **3c**, which showed 55% inhibition at 13.0 μM, all complexes were effective, with roughly 70% inhibition at lower dosages, and no significant difference between the respective halide ligands could be noted. The lower activity of **3c** might be explained by its lower kinetic solubility in the aqueous environment (see Figure 4).

**Table 4:** Inhibition of Biofilm Formation of *P. aeruginosa* ± Standard Deviation (*n* = 4) at the Respective Smallest Active Concentration of the Compounds (μM)

compounds	biofilm inhibition [% ± SD]
**3a(Cl)**	71 ± 4 (7.9 μM)
**3b(Br)**	74 ± 5 (7.1 μM)
**3c(I)**	55 ± 19 (13.0 μM)
**4a(Cl)**	71 ± 3 (7.6 μM)
**4b(Br)**	70 ± 5 (6.8 μM)
**4c(I)**	66 ± 6 (6.2 μM)
myxovalargin A	68 ± 0 (18.6 μM)

### Inhibition of Bacterial TrxR and GR

The inhibition of purified *E. coli* thioredoxin reductase (TrxR) and glutathione reductase (GR) from both *E. coli* and baker’s yeast was determined according to a previously applied procedure.^59,60^ The selected silver complexes **3a**–**c** and the reference compound AgNO_3_ were strong inhibitors of both enzymes, with IC_50_ values in the nanomolar range (0.054–0.122 μM for TrxR and 0.031–0.049 μM for both GRs), suggesting that inhibition of both thioredoxin and glutathione reductases could be important mechanisms for the bioactivity of silver compounds (Table 5). The enzyme–inhibitory activity of **3a**–**c** was comparable, showing that the nature of the halide ligand was not important for enzyme inhibition. Surprisingly, the two GRs were inhibited approximately twice as effectively as TrxR. This may be a factor in the high efficacy of silver compounds against Gram-negative strains, which depend in their growth on both GR and TrxR.

**Table 5:** Inhibition of TrxR and GR by Silver Compounds^a^

compounds	TrxR from E. coli	GR from baker’s yeast	GR from E. coli
**3a(Cl)**	0.103 ± 0.008	0.044 ± 0.004	0.032 ± 0.002
**3b(Br)**	0.092 ± 0.008	0.041 ± 0.006	0.041 ± 0.007
**3c(I)**	0.122 ± 0.015	0.049 ± 0.004	0.039 ± 0.006
AgNO_3_	0.054 ± 0.005	0.033 ± 0.003	0.031 ± 0.001

a IC_50_ values ± standard deviation are given in μM (*n* = 3).

### Cytotoxicity Evaluation against Caco-2 Cell Layers

**Figure 6:**
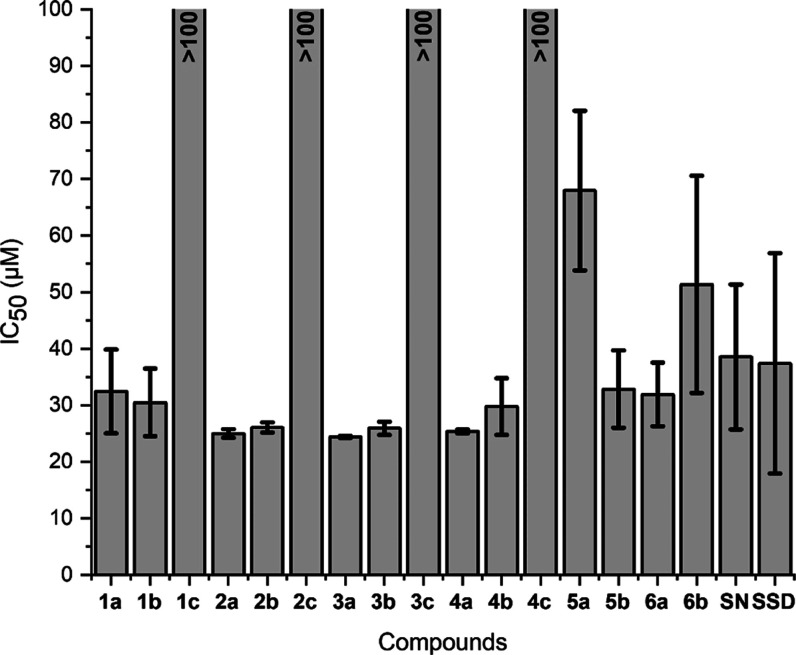
Cytotoxicity of silver NHC complexes and references (SN—AgNO_3_; SSD—silver sulfadiazine) against almost confluent cell layers of Caco-2 cells (as IC_50_ values).

Besides the antimicrobial effects, the toxic effects of silver ions have to be considered.^10,12^ As a primary evaluation of the cell toxicity triggered by silver NHC complexes, almost confluent cell layers of CaCo-2 cells were grown and challenged for 24 h with the silver complexes and also with the silver reference compounds AgNO_3_ and SSD (see Figure 6). Both chloride and bromide complexes and the reference silver compounds showed relevant cytotoxicity, with IC_50_ values of approximately 25 to 65 μM. However, the iodide complexes did not trigger any cytotoxic effects up to the highest applied concentration of 100 μM (Figures 6 and S15). This result is in good agreement with our previous report on various silver NHC complexes and might indicate that the observed nontoxicity of (NHC)AgI complexes could be of general validity.^33^

## Conclusions

Silver NHC halido complexes of the type (NHC)AgX (X = Cl, Br, and I) were prepared, characterized, and their solution chemistry in DMSO was investigated. The spectroscopic and conductometry results together confirm that complexes in the monocarbene form with iodide ligands are more stable than the respective chloride and bromide analogues. The studies also indicate that the dynamic solution equilibrium between the monocarbene and biscarbene organometallics is in favor of the monocarbene form, and such behavior is particularly evident for the complexes with iodide ligands. The higher stability of the iodide species, however, was accompanied by a reduced solubility under physiologically relevant conditions in PBS. Nephelometric experiments confirmed that the investigated (NHC)AgX complexes were generally of low aqueous solubility, however, this was not the consequence of AgCl precipitation as in the case of free silver ions. Therefore, (NHC)AgX complexes might provide a tool to administer silver ions under physiological conditions involving high chloride concentrations. The introduction of strongly solubilizing groups on the organic NHC scaffold, or the introduction of targeting moieties, might therefore be promising future strategies to design relatively stable and soluble carriers for silver ions. The controlled release of silver ions has also been considered as the reason for the biological activity of silver nanoparticles in consumer products and silver formulations.^61^ The evaluation of the antibacterial effects of the silver NHC complexes provided promising results against various pathogenic Gram-negative bacteria; however, the growth of Gram-positive bacteria was hardly affected by all silver compounds. The protein-rich components of the culture medium used for the Gram-positive strains, namely, tryptone and yeast extract, were responsible for this inactivation of the silver compounds. It can be speculated that binding to sulfur-containing molecules within the components is responsible for the reduction of the biological activity. It is worth noting that a similar inactivation effect has neither been observed for gold NHC complexes with a similar structure nor for the used reference antibiotics.^59,62^ It can thus be concluded that the negative effects of the two components might be specific for silver compounds in general and might limit their therapeutic application to topological administration.

The inhibition of biofilm formation was exemplarily confirmed for selected complexes with *P. aeruginosa*, and this is in good agreement with reports on other silver species.^56−58^

Further studies on the mechanism of drug action confirmed that the silver NHC complexes were very strong inhibitors of two investigated GRs as well as TrxR. As Gram-negative bacteria depend on both GR and TrxR systems, this might additionally explain the high efficacy of the complexes against these strains. The strong inhibition of TrxR is in good agreement with literature reports, indicating that inhibition of this enzyme might be of general relevance for the bioactivity of silver species.^30,63^

However, while the (NHC)AgX complexes with chloride or bromide as halide secondary ligand triggered strong cytotoxic effects, all the complexes with iodide ligands were nontoxic against Caco-2 cells up to the highest applied concentration of 100 μM. (NHC)AgI complexes might therefore provide an interesting option for the future design of nontoxic antibacterial silver organometallics with a broad spectrum of activity against Gram-negative bacteria with a mechanism of action involving the perturbation the glutathione and thioredoxin reductase systems. Importantly, this type of organometallics represents silver complexes with enhanced solution stability and improved solubilization in biological media. For future systemic application, the binding to proteins has to be tackled by means of structural modification or galenics.

## Methods

### General

The reagents were purchased from Acros, abcr, or TCI and used without additional purification steps. All reactions were performed without precautions to exclude air or moisture. ^1^H and ^13^C NMR spectra were recorded on a Bruker AVIIIHD 500 or AVII 600. Positive- and negative-ion ESI (electrospray ionization) mass spectra were recorded on an Expression CMS spectrometer (Advion). The elemental analyses were performed using a Flash EA 1112 (Thermo Quest CE Instruments). Absorption measurements for inhibition of enzymes and antiproliferative activity were performed on a PerkinElmer 2030 Multilabel Reader VICTOR X4. For the determination of crystal structures, see the Supporting Information. 1-Ethyl-1*H*-benzimidazole and 1-isopropyl-1*H*-benzimidazole,^63^ 1-(2-methoxyethyl)-1*H*-benzimidazole,^64^ and 1-(pyridin-2-ylmethyl)-1*H*-benzoimidazole^65^ were prepared as described in the literature. Compounds **L1a**,^65^**L1b**,^66^**L1c**,^67^**L2b**,^66^**L4a**,^68^**L5a**,^69^**L5b**,^70^**L6a**,^69^**L6b**,^66^**1a**,^71^**4a**,^68^**5a**,^69^**6a**,^69^ and **6b**(72) were already described. The exact recipes of all culture media used for biological tests are summarized in Table S7.

### Synthesis

#### Benzimidazolium Chlorides and Bromides

The fresh picolyl halide salt used in the reaction (1.2 equiv) was first prepared by neutralization with NaHCO_3_ (1.3 equiv) in 20 mL of distilled water. The organics were extracted with CHCl_3_ (4 × 15 mL), and the resulting solution was washed with brine and dried over Na_2_SO_4_. After filtration and evaporation of solvent, the pure picolyl halide was redissolved in acetonitrile (20 mL). The respective N-substituted benzimidazole (1 equiv) was dissolved in hot acetonitrile (30 mL) and added to the picolyl halide solution. The mixture was stirred under reflux conditions for 48 h. The solvent was then evaporated, and a gummy mass was solidified by treatment with a glass rod. The resulting solid was washed three times with an acetone-diethyl ether mixture (5:95), filtered, and washed with diethyl ether or pentane. The resulting powder was finally dried under reduced pressure.

##### 1-Ethyl-3-(pyridin-2-ylmethyl)-benzimidazolium Chloride (**L2a**)

The compound was prepared from 1-ethyl-1*H*-benzimidazole (1500 mg, 10.26 mmol) and isolated as a brown powder, yield: 2022 mg (7.39 mmol, 72%); ^1^H NMR (600 MHz, CDCl_3_-*d*_1_): δ 11.99 (s, BeIm-H2, 1H), 8.50 (ddd, *J*_H,H_ = 4.8, 1.8, 0.8 Hz, Py-H5, 1H), 7.98 (ddt, *J*_H,H_ = 9.7, 7.9, 0.9 Hz, Py–H2–H3, 2H), 7.75 (td, *J*_H,H_ = 7.7, 1.8 Hz, BeIm-H4/H7, 1H), 7.72–7.66 (m, BeIm-H4/H7, 1H), 7.65–7.57 (m, BeIm-H4/H7, 2H), 7.25 (ddd, *J*_H,H_ = 7.6, 4.9, 1.0 Hz, Py-H4, 1H), 6.08 (s, Py-***CH***_***2***_, 2H), 4.62 (q, *J*_H,H_ = 7.3 Hz Et–***CH***_***2***_, 2H), 1.77 (t, *J*_H,H_ = 7.3 Hz, Et–***CH***_***3***_, 3H); ^13^C NMR (151 MHz, CDCl_3_-*d*_1_): δ 152.71 (Py-C1), 149.51 (Py-C5), 143.48 (BeIm-C2), 137.68 (Py-C3), 131.91, 131.03, 127.02, 126.95 (BeIm–C4–C7), 124.13, 123.85, 123.77 (Py-C2/C4), 114.87, 112.47 (BeIm–C4–C7), 52.50 (Py-***CH***_***2***_), 42.89 (Et–***CH***_***2***_) 14.65 (Et–***CH***_***3***_); elemental analysis for C_15_H_16_ClN_3_ (theoretical/found [%]): C (65.81/65.62), H (5.89/6.06), N (15,35/15,47); MS (ESI): *m*/*z* 238.4 [M – Cl]^+^.

##### 1-Isopropyl-3-(pyridin-2-ylmethyl)-benzimidazolium Chloride (**L3a**)

The compound was prepared from 1-isopropyl-1*H*-benzimidazole (1500 mg, 9.36 mmol and isolated as a brown powder, yield: 1886 mg (6.55 mmol, 70%); ^1^H NMR (600 MHz, CDCl_3_-*d*_1_): δ 12.12 (s, BeIm-H2, 1H), 8.50 (ddd, *J*_H,H_ = 4.9, 1.8, 0.6 Hz, Py–H5, 1H), 8.06–8.02 (m, Py–H2–H3, 2H), 7.78–7.67 (m, BeIm-H4/H7, 2H), 7.65–7.52 (m, BeIm-H4/H7, 2H), 7.25 (ddd, *J*_H,H_ = 7.5, 4,9, 0.9 Hz Py-H4, 1H), 6.13 (s, Py-***CH***_***2***_, 2H), 4.94 (hept, *J*_H,H_ = 6.7 Hz, iPr-***CH***, 1H) 1.84 (d, *J*_H,H_ = 6.6 Hz, iPr-***CH***_***3***_, 6H); ^13^C NMR (151 MHz, CDCl_3_-*d*_1_): δ 152.94 (Py-C1), 149.38 (Py-C5), 142.53 (BeIm-C2), 137.71 (Py-C3), 132.18, 130.56, 126.93, 126.77 (BeIm–C4–C7), 124.51, 123.82 (Py-C2/C4), 115.15, 112.85 (BeIm–C4–C7), 52.49 (Py-***CH***_***2***_), 51.73 (iPr-***CH***) 22.28 (iPr-***CH***_***3***_); elemental analysis for C_16_H_18_ClN_3_ (theoretical/found [%]): C (66.78/66.66), H (6.30/6.24), N (14.60/14.51); MS (ESI): *m*/*z* 252.4 [M – Cl]^+^.

##### 1-Isopropyl-3-(pyridin-2-ylmethyl)-benzimidazolium Bromide (**L3b**)

The compound was prepared from 1-isopropyl-1*H*-benzimidazole (1000 mg, 6.24 mmol) and isolated as a brown powder, yield: 1679 mg (5.05 mmol, 81%); ^1^H NMR (600 MHz, CDCl_3_-*d*_1_): δ 11.73 (s, BeIm-H2, 1H), 8.49 (ddd, *J*_H,H_ = 4.9, 1.8, 0.9 Hz, Py-H5, 1H), 8.06–7.99 (m, Py –H2–H3, 2H), 7.79–7.69 (m, BeIm-H4/H7, 2H), 7.65–7.52 (m, BeIm-H4/H7, 2H), 7.25 (ddd, *J*_H,H_ = 7.6, 4,9, 1.1 Hz Py-H4, 1H), 6.14 (s, Py-***CH***_***2***_, 2H), 4.96 (hept, *J*_H,H_ = 6.7 Hz, iPr-**CH**, 1H) 1.86 (d, *J*_H,H_ = 6.7 Hz, iPr-**CH**_**3**_, 6H); ^13^C NMR (151 MHz, CDCl_3_-*d*_1_): δ 152.70 (Py-C1), 149.49 (Py-C5), 141.63 (BeIm-C2), 137.64 (Py-C3), 132.18, 130.49, 127.04, 126.89 (BeIm–C4–C7), 124.28, 123.84 (Py-C2/C4), 115.04, 112.91 (BeIm–C4–C7), 52.37 (Py- ***CH***_***2***_), 51.79 (iPr-***CH***) 22.32 (iPr-***CH***_***3***_); elemental analysis for C_16_H_18_BrN_3_ (theoretical/found [%]): C (57.84/57.70), H (5.46/5.33), N (12.65/12.64); MS (ESI): *m*/*z* 252.4 [M – Br]^+^.

##### 1-(2-Methoxyethyl),3-(pyridin-2-ylmethyl)-benzimidazolium Bromide (**L4b**)

The compound was prepared from 1-(2-methoxyethyl)-1*H*-benzimidazole (1000 mg, 5.67 mmol) and isolated as a brown powder, yield: 1225 mg (3.52 mmol, 62%); the compound is generally pure but shows a duplication of BeIM-H2 and Py-***CH***_***2***_ signals (see Figure S9). ^1^H NMR (600 MHz, CDCl_3_-*d*_*1*_): δ 11.46 (s, BeIm-H2, 1H), 8.51 (ddd, *J*_H,H_ = 4.7, 1.8, 0.9 Hz, Py-H5, 1H), 7.97–7.81 (m, Py –H2–H3, 2H), 7.82–7.72 (m, BeIm-H4/H7, 2H), 7.64–7.52 (m, BeIm-H4/H7, 2H), 7.29–7.24 (m, Py-H4, 1H), 6.03 (s, Py-***CH***_***2***_, 2H), 4.81–4.79 (m, N–CH_2_***CH***_***2***_OCH_3_, 2H), 4.01–3.95 (m, N–***CH***_***2***_CH_2_OCH_3_, 2H), 3.38 (s, N–CH_2_CH_2_O***CH***_***3***_, 3H); ^13^C NMR (151 MHz, CDCl_3_-*d*_1_): δ 152.35 (Py-C1), 149.63 (Py-C5), 143.20 (BeIm-C2), 137.71 (Py-C3), 132.00, 131.49, 127.01, 126.97 (BeIm–C4–C7), 123.95, (Py-C2/C4), 114.25, 113.62 (BeIm–C4–C7), 70.11 (N–CH_2_***CH***_***2***_OCH_3_), 59.17 (N–***CH***_***2***_CH_2_OCH_3_), 52.54 (Py-***CH***_***2***_), 47.81 (N–CH_2_CH_2_O***CH***_***3***_); elemental analysis for C_16_H_17_BrN_3_O (theoretical/found [%]): C (55.18/55.28), H (5.21/5.06), N (12.07/12.10); MS (ESI): *m*/*z* 268.4 [M – Br]^+^.

#### Benzimidazolium Iodides

A 50 mL flask was charged with 1 equiv of the appropriate benzimidazolium chloride (**L1a**–**L4a**), 5 equiv of KI, and 15 mL of an acetone–methanol mixture (9:1). The resulting suspension was stirred at room temperature for 48 h. The solvents were then evaporated, 15 mL CHCl_3_ were added, and the mixture was filtered. The filtrate was evaporated until 2 mL of solvent remained, and 15 mL of diethyl ether was added. The resulting precipitate was filtered off and dried under reduced pressure.

##### 1-Ethyl-3-(pyridin-2-ylmethyl)-1*H*-benzimidazolium Iodide (**L2c**)

The compound was prepared from **L2a** (500 mg, 1.83 mmol) and isolated as a pale orange powder, yield: 565 mg (1.55 mmol, 85%); ^1^H NMR (500 MHz, CDCl_3_-*d*_1_): δ 11.26 (s, BeIm-H2, 1H), 8.50 (ddd, *J*_H,H_ = 4.8, 1.8, 0.8 Hz, Py-H5, 1H), 7.97–7.89 (m, Py–H2–H3, 2H), 7.77 (td, *J*_H,H_ = 7.7, 1.8 Hz, BeIm-H4/H7, 1H), 7.73–7.69 (m, BeIm-H4/H7, 1H), 7.67–7.59 (m, BeIm-H4/H7, 2H), 7.32–7.24 (m, Py-H4, 1H), 6.04 (s, Py-***CH***_***2***_, 2H), 4.63 (q, *J*_H,H_ = 7.4 Hz Et–***CH***_***2***_, 2H), 1.81 (t, *J*_H,H_ = 7.3 Hz, Et–CH_3_, 3H); ^13^C NMR (126 MHz, CDCl_3_-*d*_1_): δ 152.14 (Py-C1), 149.72 (Py-C5), 141.89 (BeIm-C2), 137.70 (Py-C3), 131.92, 130.98, 127.28, 127.18 (4C, BeIm–C4–C7), 124.00, 123.96 (Py-C2/C4), 114.63, 112.61 (BeIm–C4–C7), 52.37 (Py-***CH***_***2***_), 43.05 (Et–***CH***_***2***_) 14.68 (Et–***CH***_***3***_); elemental analysis for C_15_H_16_IN_3_ (theoretical/found [%]): C (49.47/49.42), H (4.15/4.29), N (11.54/11.33); MS (ESI): *m*/*z* 238.4 [M – I]^+^.

##### 1-Isopropyl-3-(pyridin-2-ylmethyl)-benzimidazolium Iodide (**L3c**)

The compound was prepared from **L3a** (500 mg, 1.74 mmol) and isolated as a pale yellow powder, yield: 565 mg (1.49 mmol, 86%); ^1^H NMR (500 MHz, CDCl_3_-*d*_1_): δ 11.33 (s, BeIm-H2, 1H), 8.49 (ddd, *J*_H,H_ = 4.9, 1.8, 1.0 Hz, Py-H5, 1H), 8.02–7.94 (m, Py–H2–H3, 2H), 7.81–7.68 (m, BeIm-H4/H7, 2H), 7.67–7.56 (m, BeIm-H4/H7, 2H), 7.31–7.23 (m, Py-H4, 1H), 6.12 (s, Py-***CH***_***2***_, 2H), 4.97 (hept, *J*_H,H_ = 6.7 Hz, iPr-***CH***, 1H) 1.87 (d, *J*_H,H_ = 6.7 Hz, iPr-***CH***_***3***_, 6H); ^13^C NMR (126 MHz, CDCl_3_-*d*_1_): δ 152.38 (Py-C1), 149.63 (Py-C5), 140.85 (BeIm-C2), 137.66 (Py-C3), 132.27, 130.44, 127.20, 127.05 (BeIm–C4–C7), 124.14, 123.93 (Py-C2/C4), 114.91, 112.98 (BeIm–C4–C7), 52.34 (Py-***CH***_***2***_), 51.96 (iPr-***CH***) 22.40 (iPr-***CH***_***3***_); elemental analysis for C_16_H_17_IN_3_ (theoretical/found [%]): C (50.81/50.96), H (4.53/4.61), N (11.11/11.13); MS (ESI): *m*/*z* 252.5 [M – I]^+^.

##### 1-(2-Methoxyethyl)-3-(pyridin-2-ylmethyl)-benzimidazolium Iodide (**L4c**)

The compound was prepared from **L4a** (500 mg, 1.65 mmol) and isolated as a yellowish powder, yield: 566 mg(1.44 mmol, 87%); ^1^H NMR (500 MHz, CDCl_3_-*d*_1_): δ 11.05 (s, BeIm-H2, 1H), 8.52 (ddd, *J*_H,H_ = 4.8, 1.8, 1.0 Hz, Py-H5, 1H), 7.92–7.84 (m, Py–H2–H3, 2H), 7.82–7.74 (m, BeIm-H4/H7, 2H), 7.63–7.55 (m, BeIm-H4/H7, 2H), 7.31–7.25 (m, Py-H4, 1H), 6.00 (s, Py-***CH***_***2***_, 2H), 4.80–4.78 (m, N–CH_2_***CH***_***2***_OCH_3_, 2H), 4.03–3.96 (m, N–***CH***_***2***_CH_2_OCH_3_, 2H), 3.39 (s, N–CH_2_CH_2_O***CH***_***3***_, 3H); ^13^C NMR (126 MHz, CDCl_3_-*d*_1_): δ 152.00 (Py-C1), 149.74 (Py-C5), 142.42 (BeIm-C2), 137.74 (Py-C3), 131.90, 131.48, 127.17, 127.12 (BeIm–C4–C7), 124.03, 123.92 (Py-C2/C4), 114.15, 113.61 (BeIm–C4–C7), 69.84 (N–CH_2_***CH***_***2***_OCH_3_), 59.28 (N–***CH***_***2***_CH_2_OCH_3_), 52.52 (Py-***CH***_***2***_), 47.87 (N–CH_2_CH_2_O***CH***_***3***_); elemental analysis for C_16_H_17_IN_3_O (theoretical/found [%]): C (48.75/49.05), H (4.35/4.19), N (10.66/10.75); MS (ESI): *m*/*z* 268.4 [M – I]^+^.

#### General Procedure for Synthesis of (NHC)AgX Complexes

The respective benzimidazolium halide was dissolved in 20 mL of dry dichloromethane, and 0.6 equiv of Ag_2_O was added to the solution along with 3 Å molecular sieves (maximum 10 wt %). The flask was covered with aluminum foil, and the mixture was stirred at room temperature for 6 h (for the chloride and bromide complexes) or 3 h (for the iodide complexes). The reduced reaction time for the iodide analogues was chosen because of formation of a large amount of white solid after 4 h of stirring. The reaction mixture was filtered through a pad of Celite, the filtrate was reduced to 5 mL, and 30 mL of diethyl ether or pentane was added to precipitate a solid. The solid was washed three times with pentane, isolated by filtration, and dried.

##### Bromido (1-Methyl-3-(pyridin-2-ylmethyl)-benzimidazol-2-ylidene) Silver(I) (**1b**)

The compound was prepared from **L1b** (300 mg, 0.99 mmol) and isolated as an off-white powder, yield: 321 mg (0.78 mmol,79%); ^1^H NMR (600 MHz, DMSO-*d*_6_): δ 8.47 (ddd, J_H,H_ = 4.8, 1.8, 1.0 Hz, Py-H5, 1H), 7.78 (td, J_H,H_ = 7.7, 1.8 Hz, Py–H2–H3, 2H), 7.77–7.71 (m, BeIm-H4/H7, 1H), 7.49–7.42 (m, BeIm-H4/H7, 2H), 7.45–7.38 (m, BeIm-H4/H7, 1H), 7.31 (ddd, J_H,H_ = 7.6, 4.8, 1.2 Hz, Py-H4, 1H), 5.83 (s, Py-***CH***_***2***_, 2H), 4.07 (s, CH_3_, 3H); ^13^C NMR (151 MHz, DMSO-*d*_6_): δ 190.25 (BeIm-C2), 155.29 (Py-C1), 149.40 (Py-C5), 137.20 (Py-C3), 133.87, 133.43 (BeIm–C4–C7), 123.78, 123.89, 123.13, 122.16 (Py-C2/C4), 112.01, 111.95 (BeIm–C4–C7), 53.06 (Py-***CH***_***2***_), 35.48 (CH_3_); elemental analysis for C_14_H_13_AgBrN_3_ (theoretical/found [%]): C (40.91/41.23), H (3.19/3.07), N (10.22/10.06); MS (ESI+): *m*/*z* 555.7 [NHC–Ag–NHC]^+^, 224.3[M-AgBr]^+^; (ESI−): 266.7 [Br–Ag–Br]^−^.

##### Iodido (1-Methyl-3-(pyridin-2-ylmethyl)-benzimidazol-2-ylidene) Silver(I) (**1c**)

The compound was prepared from **L1c** (200 mg, 0.57 mmol) and isolated as a white powder, yield: 120 mg (0.26 mmol, 46%); ^1^H NMR (500 MHz, DMSO-*d*_6_): δ 8.47 (ddd, J_H,H_ = 4.9, 1.8, 0.9 Hz, Py-H5, 1H), 7.81–7.74 (m, Py–H2–H3/BeIm-H4/H7, 3H), 7.50–7.44 (m, BeIm-H4/H7, 2H), 7.46–7.39 (m, BeIm-H4/H7, 1H), 7.30 (ddd, *J*_H,H_ = 7.6, 4.8, 1.2 Hz, Py-H4, 1H), 5.86 (s, Py-***CH***_***2***_, 2H), 4.10 (s, CH_3_, 3H); ^13^C NMR (126 MHz, DMSO-*d*_6_): δ 191.15 (BeIm-C2), 155.28 (Py-C1), 149.40 (Py-C5), 137.23 (Py-C3), 133.89, 133.43 (BeIm–C4–C7), 123.90, 123.80, 123.15, 122.25 (Py-C2/C4), 111.98, 111.94 (BeIm–C4–C7), 52.98 (Py-***CH***_***2***_), 35.43 (CH_3_); elemental analysis for C_14_H_13_AgIN_3_ (theoretical/found [%]): C (36.71/36.69), H (2.86/2.69), N (9.17/8.83); MS (ESI+): *m*/*z* 553.1 [NHC–Ag–NHC]^+^, 224.1[M – AgI]^+^; (ESI−): 360.7 [I–Ag–I]^−^.

##### Chlorido (1-Ethyl-3-(pyridin-2-ylmethyl)-benzimidazol-2-ylidene) Silver(I) (**2a**)

The compound was prepared from **L2a** (300 mg, 1.1 mmol) and isolated as an off-white powder, yield: 345 mg (0.91 mmol, 82%); ^1^H NMR (500 MHz, DMSO-*d*_6_): δ 8.49 (ddd, *J*_H,H_ = 4.8, 1.8, 0.9 Hz, Py-H5, 1H), 7.87–7.76 (m, Py –H2–H3, 2H), 7.76–7.70 (m, BeIm-H4/H7, 1H), 7.48–7.36 (m, BeIm-H4/H7, 3H), 7.26 (ddd, *J*_H,H_ = 7.6, 4.8, 1.1 Hz, Py-H4, 1H), 5,81 (s, Py-***CH***_***2***_, 2H), 4.53 (q, J_H,H_ = 7.2 Hz Et–***CH***_***2***_, 2H), 1.45 (t, *J*_H,H_ = 7.2 Hz, Et–***CH***_***3***_, 3H); ^13^C NMR (126 MHz, DMSO-*d*_6_): δ 188.36 (BeIm-C2), 155.28 (Py-C1), 149.42 (Py-C5), 137.20 (Py-C3), 133.61, 132.70, 123.89, 123.81 (BeIm–C4–C7), 123.11, 122.03 (Py-C2/C4), 112.25, 111.91 (BeIm–C4–C7), 53.26 (Py-***CH***_***2***_), 43.83 (Et–***CH***_***2***_) 15.82 (Et–***CH***_***3***_); elemental analysis for C_15_H_15_AgClN_3_ (theoretical/found [%]): C (47.33/47.74), H (3.97/4.06), N (11.04/10.76); MS (ESI+): *m*/*z* 580.9 [NHC–Ag–NHC]^+^, 238.0 [M – AgCl]^+^; (ESI−): 178.7 [Cl–Ag–Cl]^−^.

##### Bromido (1-Ethyl-3-(pyridin-2-ylmethyl)-benzimidazol-2-ylidene) Silver(I) (**2b**)

The compound was prepared from **L2b** (300 mg, 0.94 mmol) and isolated as a brownish powder, yield: 263 mg (0.62 mmol,65%); ^1^H NMR (500 MHz, DMSO-*d*_6_): δ 8.49 (ddd, *J*_H,H_ = 4.8, 1.8, 0.9 Hz, Py-H5, 1H), 7.88–7.80 (m, BeIm-H4/H7, 1H), 7.83–7.72 (m, Py–H2–H3, 2H), 7.49–7.37 (m, BeIm-H4/H7, 3H), 7.26 (ddd, *J*_H,H_ = 7.6, 4.9, 1.2 Hz, Py-H4, 1H), 5,83 (s, Py-***CH***_***2***_, 2H), 4.54 (q, *J*_H,H_ = 7.2 Hz Et–***CH***_***2***_, 2H), 1.45 (t, *J*_H,H_ = 7.2 Hz, Et–***CH***_***3***_, 3H); ^13^C NMR (126 MHz, DMSO-*d*_6_): δ 189.55 (BeIm-C2), 155.27 (Py-C1), 149.42 (Py-C5), 137.23 (Py-C3), 133.60, 132.70, 123.91, 123.83 (BeIm–C4–C7), 123.14, 122.10 (Py-C2/C4), 112.24, 111.91 (BeIm–C4–C7), 53.18 (Py-***CH***_***2***_), 43.75 (Et–***CH***_***2***_) 15.84 (Et–***CH***_***3***_); elemental analysis for C_15_H_15_AgBrN_3_ (theoretical/found [%]): C (42.38/42.71), H (3.56/3.47), N (9.89/9.48); MS (ESI+): *m*/*z* 580.9 [NHC–Ag–NHC]^+^, 238.0 [M – AgBr]^+^; (ESI−): 266.7 [Br–Ag–Br]^−^.

##### Iodido (1-Ethyl-3-(pyridin-2-ylmethyl)-benzimidazol-2-ylidene) Silver(I) (**2c**)

The compound was prepared from **L2c** (200 mg, 0.55 mmol) and isolated as a white powder, yield: 109 mg (0.23 mmol,42%); ^1^H NMR (500 MHz, DMSO-*d*_6_): δ 8.47 (ddd, *J*_H,H_ = 4.8, 1.8, 0.9 Hz, Py-H5, 1H), 7.86–7.83 (BeIm-H4/H7, 1H), 7.79–7.75 (m, Py–H2–H3, 1H), 7.45 (dt, *J*_H,H_ = 7.6, 1.1 Hz, BeIm-H4/H7, 2H), 7.44–7.40 (m, BeIm-H4/H7, 1H), 7.31 (ddd, *J*_H,H_ = 7.6, 4.9, 1.2 Hz, Py-H4, 1H), 5.87 (s, Py-***CH***_***2***_, 2H), 4.57 (q, *J*_H,H_ = 7.2 Hz Et–***CH***_***2***_, 2H), 1.44 (t, *J*_H,H_ = 7.2 Hz, Et–***CH***_***3***_, 3H); ^13^C NMR (126 MHz, DMSO-*d*_6_): δ 190.76 (BeIm-C2), 155.32 (Py-C1), 149.42 (Py-C5), 137.25 (Py-C3), 133.64, 132.78, 123.91, 123.84 (BeIm–C4–C7), 123.17, 122.17 (Py-C2/C4), 112.20, 111.88 (BeIm–C4–C7), 53.14 (Py-***CH***_***2***_), 43.66 (Et–***CH***_***2***_) 15.87 (Et–***CH***_***3***_); elemental analysis for C_15_H_15_AgIN_3_ (theoretical/found [%]): C (38.16/37.78), H (3.20/3.22), N (8.90/8.72); MS (ESI+): *m*/*z* 580.9 [NHC–Ag–NHC]^+^, 238.0[M-AgI]^+^; (ESI−): 360.7 [I–Ag–I]^−^.

##### Chlorido (1-Isopropyl-3-(pyridin-2-ylmethyl)-benzimidazol-2-ylidene) Silver(I) (**3a**)

The compound was prepared from **L3a** (300 mg, 1.04 mmol) and isolated as a white powder, yield: 305 mg (0.77 mmol, 74%); ^1^H NMR (500 MHz, DMSO-*d*_6_): δ 8.50 (ddd, *J*_H,H_ = 5.3, 1.8, 0.9 Hz, Py-H5, 1H), 7.98–7.90 (m, Py–H2–H3, 1H), 7.80 (tdd, *J*_H,H_ = 7.7, 4.0, 1,8 Hz BeIm-H4/H7, 1H), 7.77–7.69 (m, BeIm-H4/H7, 1H), 7.46–7.35 (m, BeIm-H4/H7, 3H) 7.32 (ddd, *J*_H,H_ = 7.6, 4,8, 1.1 Hz Py-H4, 1H), 5.81 (s, Py-***CH***_***2***_, 2H), 5.11 (hept, *J*_H,H_ = 6.9 Hz, iPr-***CH***, 1H) 1.68 (d, *J*_H,H_ = 6.9 Hz, iPr-***CH***_***3***_, 6H); ^13^C NMR (126 MHz, DMSO-*d*_6_): δ 186.79 (BeIm-C2), 155.24 (Py-C1), 149.44 (Py-C5), 137.23 (Py-C3), 133.82, 132.13, 123.98, 123.64 (BeIm–C4–C7), 123.13, 122.01 (Py-C2/C4), 112.57, 112.38 (BeIm–C4–C7), 53.62 (Py-***CH***_***2***_), 52.04 (iPr-***CH***) 22.35 (iPr-***CH***_***3***_); elemental analysis for C_16_H_17_AgClN_3_ (theoretical/found [%]): C (48.70/48.92), H (4.34/4.32), N (10.65/10.52); MS (ESI+): *m*/*z* 609.0 [NHC–Ag–NHC]^+^, 252.0[M – AgCl]^+^; (ESI−): 178.7 [Cl–Ag–Cl]^−^

##### Bromido (1-Isopropyl-3-(pyridin-2-ylmethyl)-benzimidazol-2-ylidene) Silver(I) (**3b**)

The compound was prepared from **L2b** (300 mg, 0.90 mmol) and isolated as an off-white powder, yield: 296 mg (0.67 mmol, 74%); ^1^H NMR (500 MHz, DMSO-*d*_6_): δ 8.50 (ddd, *J*_H,H_ = 4.9, 1.8, 0.9 Hz, Py-H5, 1H), 7.99–7.90 (m, Py–H2–H3, 1H), 7.83–7.72 (m, BeIm-H4/H7, 2H), 7.47–7.37 (m, BeIm-H4/H7, 3H), 7.32 (ddd, *J*_H,H_ = 7.5, 4,8, 1.1 Hz Py-H4, 1H), 5.83 (s, Py-***CH***_***2***_, 2H), 5.12 (hept, *J*_H,H_ = 6.9 Hz, iPr-***CH***, 1H) 1.68 (d, *J*_H,H_ = 6.9 Hz, iPr-***CH***_***3***_, 6H); ^13^C NMR (126 MHz, DMSO-*d*_6_): δ 187.88 (BeIm-C2), 155.20 (Py-C1), 149.44 (Py-C5), 137.26 (Py-C3), 133.79, 132.20 (BeIm–C4–C7), 124.00, 123.67, 123.16, 122.06 (Py-C2/C4), 112.57, 112.34 (BeIm–C4–C7), 53.53 (Py-***CH***_***2***_), 51.89 (iPr-***CH***) 22.40 (iPr-***CH***_***3***_); elemental analysis for C_16_H_17_AgBrN_3_ (theoretical/found [%]): C (43.77/44.25), H (3.90/3.78), N (9.57/9.36); MS (ESI+): *m*/*z* 609.0 [NHC–Ag–NHC]^+^, 252.0 [M – AgBr]^+^; (ESI−): 266.7 [Br–Ag–Br]^−^.

##### Iodido (1-Isopropyl-3-(pyridin-2-ylmethyl)-benzimidazol-2-ylidene) Silver(I) (**3c**)

The compound was prepared from **L3c** (200 mg, 0.53 mmol) and isolated as a white powder, yield: 123 mg (0.25 mmol, 48%); ^1^H NMR (600 MHz, DMSO-*d*_6_): δ 8.48 (ddd, *J*_H,H_ = 4.9, 1.8, 0.9 Hz, Py-H5, 1H), 7.98–7.91 (m, Py –H2–H3, 1H), 7.82–7.75 (m, BeIm-H4/H7, 2H), 7.45–7.41 (m, BeIm-H4/H7, 3H) 7.32 (ddd, *J*_H,H_ = 7.6, 4.8, 1.1 Hz Py-H4, 1H), 5.86 (s, Py-***CH***_***2***_, 2H), 5.16 (hept, *J*_H,H_ = 6.8 Hz, iPr-***CH***, 1H) 1.66 (d, *J*_H,H_ = 6.9 Hz, iPr-***CH***_***3***_, 6H); ^13^C NMR (151 MHz, DMSO-*d*_6_): δ 189.13 (BeIm-C2), 155.27 (Py-C1), 149.54 (Py-C5), 137.39 (Py-C3), 133.94, 132.30, 124.11, 123.79 (BeIm–C4–C7), 123.29, 122.22 (Py-C2/C4), 112.71, 112.39 (BeIm–C4–C7), 53.52 (Py-***CH***_***2***_), 51.96 (iPr-***CH***) 22.49 (iPr-***CH***_***3***_); elemental analysis for C_16_H_17_AgIN_3_ (theoretical/found [%]): C (39.53/39.65), H (3.53/3.55), N (8.64/8.70); MS (ESI+): *m*/*z* 611.2 [NHC–Ag–NHC]^+^, 252.5[M – AgI]^+^; (ESI-): 360.7 [I–Ag–I]^−^.

##### Bromido (1-(2-Methoxyethyl)-3-(pyridin-2-ylmethyl)-benzimidazol-2-ylidene) Silver(I) (**4b**)

The compound was prepared from **L4b** (300 mg, 0.86 mmol) and isolated as an off-white powder, yield: 269 mg (0.59 mmol, 69%); ^1^H NMR (600 MHz, DMSO-*d*_6_): δ 8.48 (ddd, *J*_H,H_ = 4.9, 1.8, 0.9 Hz, Py-H5, 1H), 7.86–7.68 (m, Py–H2–H3/BeIm-H4/H7, 3H), 7.47–7.36 (m, BeIm-H4/H7, 3H), 7.31 (ddd, *J*_H,H_ = 7.6, 4.9, 1.2 Hz, Py-H4, 1H), 5.84 (s, Py-***CH***_***2***_, 2H), 4.68 (t, *J*_H,H_ = 5.2 Hz N–CH_2_***CH***_***2***_OCH_3_, 2H), 3.79–3.76 (m, N–***CH***_***2***_CH_2_OCH_3_, 2H), 3.21 (s, N–CH_2_CH_2_O***CH***_***3***_, 3H); ^13^C NMR (151 MHz, DMSO-*d*_6_): δ 190.38 (BeIm-C2), 155.22 (Py-C1), 149.40 (Py-C5), 137.21 (Py-C3), 133.62, 133.42, 123.85, 123.76 (BeIm–C4–C7), 123.14, 122.04 (Py-C2/C4), 112.28, 112.15 (BeIm–C4–C7), 70.77 (N–CH_2_***CH***_***2***_OCH_3_), 58.17 (N–***CH***_***2***_CH_2_OCH_3_), 53.25 (Py-***CH***_***2***_), 48.38 (N–CH_2_CH_2_O***CH***_***3***_); elemental analysis for C_16_H_17_AgBrN_3_O (theoretical/found [%]): C (42.23/41.89), H (3.77/3.30), N (9.23/9.01); MS (ESI+): *m*/*z* 641.2 [NHC–Ag–NHC]^+^, 268.1 [M – AgBr]^+^; (ESI−): 266.7 [Br–Ag–Br]^−^

##### Iodido (1-(2-Methoxyethyl)-3-(pyridin-2-ylmethyl)-benzimidazol-2-ylidene) Silver(I) (**4c**)

The compound was prepared from **L5a** (200 mg, 0.51 mmol) and isolated as a white powder, yield: 115 mg (0.23 mmol, 45%); ^1^H NMR (600 MHz, DMSO-*d*_6_): δ 8.46 (ddd, *J*_H,H_ = 4.7, 1.8, 0.9 Hz, Py-H5, 1H), 7.87–7.74 (m, Py–H2–H3, 2H), 7.78–7.71 (m, BeIm-H4/H7, 1H), 7.48–7.36 (m, BeIm-H4/H7, 3H), 7.30 (ddd, *J*_H,H_ = 7.6, 4.9, 1.2 Hz, Py-H4, 1H), 5.87 (s, Py-***CH***_***2***_, 2H), 4.71 (t, *J*_H,H_ = 5.2 Hz, N–CH_2_***CH***_***2***_OCH_3_, 2H), 3.77 (t, *J*_H,H_ = 5.2 Hz, N–***CH***_***2***_CH_2_OCH_3_, 2H), 3.22 (s, N–CH_2_CH_2_O***CH***_***3***_, 3H); ^13^C NMR (151 MHz, DMSO-*d*_6_): δ 191.32 (BeIm-C2), 155.22 (Py-C1), 149.38 (Py-C5), 137.21 (Py-C3), 133.68, 133.40, 123.84, 123.76 (BeIm–C4–C7), 123.14, 122.09 (Py-C2/C4), 112.26, 112.12 (BeIm–C4–C7), 70.88 (N–CH_2_***CH***_***2***_OCH_3_), 58.19 (N–***CH***_***2***_CH_2_OCH_3_), 53.19 (Py-***CH***_***2***_), 48.30 (N–CH_2_CH_2_O***CH***_***3***_); elemental analysis for C_16_H_17_AgIN_3_O (theoretical/found [%]): C (38.27/38.63), H (3.41/3.17), N (8.37/8.28); MS (ESI+): *m*/*z* 641.2[NHC–Ag–NHC]^+^, 268.1[M – AgI]^+^; (ESI−): 360.7 [I–Ag–I]^−^.

##### Bromido (1-(Pyridin-2-ylmethyl)-3-benzyl-benzimidazol-2-ylidene) Silver(I) (**5b**)

The compound was prepared from **L5b** (300 mg, 0.79 mmol) and isolated as a white powder, yield: 230 mg(0.47 mmol, 60%); ^1^H NMR (500 MHz, DMSO-*d*_6_): δ 8.45 (ddd, *J*_H,H_ = 5.0, 1.8, 0,9 Hz, Py-H5, 1H), 7.81–7.60 (m, BeIm–H4–H7, 3H), 7.45 (dd, *J*_H,H_ = 7.9, 1.3 Hz, BeIm-H4/H7, 1H), 7.38 (dt, *J*_H,H_ = 7.3, 1.9 Hz, Bn–H2–H6, 4H), 7.35–7.26 (m, Py–H2–H4, 3H), 5.85 (s, Py-***CH***_***2***_, 2H), 5.76 (s, Bn-***CH***_***2***_, 2H); ^13^C NMR (126 MHz, DMSO-*d*_6_) 190.52 (BeIm-C2): δ 155.12 (Py-C1), 149.42 (Py-C5), 137.21 (Py-C3), 136.16 (Bn-C1), 133.76, 133.11 (BeIm–C4–C7), 128.67, 127.91, 127.25 (Bn–C2–C6) 124.02, 123.95 (BeIm–C4–C7), 123.15, 122.11 (Py-C2/C4), 112.33, 112.27 (BeIm–C4–C7), 53.24 (Py-***CH***_***2***_), 51.77 (Bn-***CH***_***2***_); elemental analysis for C_20_H_17_AgBrN_3_ (theoretical/found [%]): C (49.31/48.93), H (3.52/3.47), N (8.63/8.49); MS (ESI+): *m*/*z* 704.9 [NHC–Ag–NHC]^+^, 300.0 [M – AgBr]^+^; (ESI−): 266.7 [Br–Ag–Br]^−^.

#### Conductometry

Conductivity measurements were performed using an Apera Instruments EC9500 conductivity meter. Solutions (as indicated, otherwise 1.0 mM) of all compounds were prepared using freshly distilled DMSO with a specific electrical conductivity of ≤3 × 10^–8^ S cm^–1^. The conductivities were measured at 25 ± 2 °C immediately after complete dissolution and after 24 h.

#### Stability and Kinetic Solubility

The kinetic solubility was determined using laser nephelometry. A 25 mM stock solution of the test compounds in DMSO was prepared and further diluted with DMSO to achieve a dilution series of eight solutions with different concentrations. A volume of 0.5 μL of each of the DMSO solutions was then added in a row on a 96-well plate filled with 250 μL of aqueous phosphate buffer pH 7.4. The well plate was thoroughly shaken and scanned by a nephelometer (NepheloStar Plus, BMG Labtech, Ortenberg, Germany) at 25 °C. Unsolved particles scatter the laser light, which is detected by the nephelometer. The intensity of the scattered light is expressed as NTUs and is proportional to the particle concentration in the suspension. The NTU was plotted against the concentration to detect the solubility of compounds. The resulting curves were obtained in three independent experiments. After the experiment, the DMSO stock solutions of compounds were kept away from direct light at room temperature for 24 h to monitor the stability of solutions.

#### Antibacterial Screening and Effect of Culture Media

The following strains of ESKAPE panel were utilized and maintained at 37 °C in MHB (21 g/L Müller Hinton broth, pH 7.4) or TSY (30 g/L trypticase soy broth, 3 g/L yeast extract, pH 7.0–7.2) media: *A. baumannii* (DSM 30007, ATCC 19606), *E. coli* (DSM1116, ATCC 9637), *K. pneumoniae* (DSM 11678, ATCC33495), *P. aeruginosa* PA7 (DSM 24068) in MHB; *E. faecium* (DSM 20477, ATCC 19434), and *S. aureus* MRSA (DSM 11822, ICB 25701) in TSY. MIC values were determined following a standardized protocol in broth microdilution assays. The compounds and reference substances (AgNO_3_ and SSD) were serially diluted from 64 to 1 μg/mL. Starting inocula of 2–8 × 10^5^ CFU/mL in MHB or TSY media at 37 °C were used, and serial dilutions were carried out in 96-well microtiter plates in duplicate. After incubation of the plates for 22 h at 37 °C, the absorbance at 600 nm was measured using The Spark multimode microplate reader (Tecan Trading AG). The MIC values for the tested compounds were determined in three independent experiments and presented in μM with standard errors and also in μg/mL. Amikacin (*P. aeruginosa*), linezolid (*S. aureus*), and ciprofloxacin (all other strains) served as positive controls. The MIC and EC_50_ values were determined by curve fitting with Sigma Plot. To determine the effect of the culture medium on the activity of silver compounds, the assay was performed using MHB, TSY, or DMEM (Gibco). To observe the effect of individual components of TSY medium, antibacterial tests were performed using MHB with addition of tryptone (17 g/L) or yeast extract (3 g/L, both were purchased from Sigma).

#### Antibiofilm Activity

The antibiofilm activity was determined according to a literature method.^73^ A 1 mL aliquot of *P. aeruginosa* (PA 14) was taken from −20 °C stock, incubated in 25 mL LB medium (Luria–Bertani Broth) in a 250 mL flask, and kept at 37 °C at 100 rpm overnight. The optical density at 600 nm (OD_600_) of the culture solution was adjusted to match the turbidity of a 0.1 McFarland standard in M63 medium.^74^ Selected compounds were mixed with 150 μL of bacterial solution in U-bottom 96 well plates to the final respective concentrations (25–0.2 μg/mL). The plates were further incubated at 37 °C for 24 h while shaking at 150 rpm. Afterward, the plates were rinsed once with 150 μL of PBS buffer, and the biofilms were stained using 150 μL of crystal violet (0.1%) staining at room temperature for 15 min and then rinsed once with PBS buffer. After rinsing, 150 μL of ethanol (95%) was added, and the absorbance was quantified using a plate reader (Synergy 2, BioTek, Santa Clara, USA) at 550 nm. The resulting antibiofilm activity is presented as percentage of inhibition at the respective smallest active concentration in μM with standard deviation (SD) of two repeats with duplicates. Myxovalargin A and DMSO (2.5%) were used as the positive and negative controls, respectively.

#### Inhibition of Bacterial TrxR (*E. coli*) and GR (Baker’s Yeast and *E. coli*)

The TrxR (*E. coli*) and GR (baker’s yeast and *E. coli*) inhibition assays were performed according to previously published procedures.^62,75^ The assays are partly based on the procedure developed by Lu et al.^60^ and make use of the reduction of 5,5′-dithiobis(2-nitrobenzoic acid) (DTNB). Solutions of *E. coli* TrxR (35.4 U/mL) and *E. coli* thioredoxin (Trx) (156 μg/mL) (both purchased from Sigma-Aldrich) or baker’s yeast GR (0,08 U/mL, Sigma-Aldrich) or *E. coli* GR (4.2 U/ml, Antibodies online) and GSSG (0.28 mM, Sigma-Aldrich) in distilled water were prepared, as were fresh 2 mM stock solutions of the test compounds in DMSO. Then, solutions with several concentrations of the test compounds in DMSO were prepared, and 35 μL of each solution was diluted with 965 μL of TE buffer (Tris–HCl 50 mM, EDTA 1 mM, pH 7.5). These solutions (20 μL) or TE buffer without the test compounds (20 μL, as control) were mixed with the TrxR or GR solutions (10 μL), the Trx or GSSG solutions (10 μL), and a solution of NADPH (200 μM) in TE buffer (100 μL) in a well on a 96-well plate. As a blank solution, 200 μM NADPH in TE buffer (100 μL) mixed with a DMSO/buffer mixture (40 μL) was used (final concentrations of DMSO: 0.5% v/v). The plate was incubated for 75 min at 25 °C with moderate shaking. After incubation, 100 μL of a reaction mixture (TE buffer containing 200 μM NADPH and 5 mM DTNB) was added to each well to initiate the reaction. After thorough mixing, the formation of 5-TNB was monitored by a microplate reader at 405 nm in 35 s intervals (10 measurements). The values were corrected by subtraction of the blank solution absorption values. The increase in the concentration of 5-TNB followed a linear trend (*r*^2^ ≥ 0.990), and the enzymatic activities were calculated as the gradients (increase in absorbance per second) thereof. The absence of interference with the assay components was confirmed by a negative control experiment for each test compound, where the highest test compound concentration was used and the enzyme solution was replaced by TE buffer. The inhibition is presented as the mean IC_50_ values and standard deviations obtained in three independent experiments.

#### Toxicity against Mammalian Cells (CaCo-2)

Caco-2 cells were grown as almost confluent monolayers in 96-well plates. The complexes were dissolved in wells of 96-well plates as stock solutions in DMSO (0.2% v/v) diluted with DMEM cell culture medium, which was supplemented with 10% fetal calf serum and 50 mg/L gentamicin. The cell layers were incubated with the drug containing media for 24 h at 37 °C/5% CO_2_ in an incubator. The cell viability was determined using crystal violet (0.02%) staining and was calculated as percentage of an untreated control. Results were obtained in three independent experiments.

## Supporting Information Available

Additional data, ^1^H NMR, ^13^C NMR, and MS spectra (PDF)
