# Supplementary material for: Silver Organometallics that are Highly Potent Thioredoxin and Glutathione Reductase Inhibitors: Exploring the Correlations of Solution Chemistry with the Strong Antibacterial Effects
Source: ACS Infect Dis. 2024 Apr 12;10(5):1753–66. doi: 10.1021/acsinfecdis.4c00104 (PMC11091889; doi:10.1021/acsinfecdis.4c00104)
Supplement: Supplementary file 1 — id4c00104_si_001.pdf [file id4c00104_si_001.pdf]

Supporting Information

Silver organometallics that are highly potent thioredoxin and glutathione reductase inhibitors: Exploring the correlations of solution chemistry with the strong antibacterial effects

Igor V. Esarev [a], Bianka Karge[b], Haoxuan Zeng[c,d], Petra Lippmann[a], Peter G. Jones[e], Hedda Schrey[c,d] Mark Brönstrup[b], Ingo Ott[a*]

a) Institute of Medicinal and Pharmaceutical Chemistry, Technische Universität Braunschweig, Beethovenstraße 55, 38106 Braunschweig, Germany
b) Department of Chemical Biology, Helmholtz Centre for Infection Research, Inhoffenstraße 7, 38124 Braunschweig, Germany
c) Department of Microbial Drugs, Helmholtz Centre for Infection Research GmbH and German Centre for Infection Research (DZIF), Partner Site Hannover/Braunschweig, Inhoffenstraße 7, 38124 Braunschweig, Germany
d) Institute of Microbiology, Technische Universität Braunschweig, Spielmannstraße 7, 38106 Braunschweig, Germany
e) Institute of Inorganic and Analytical Chemistry, Technische Universität Braunschweig, Hagenring 30, 38106 Braunschweig, Germany

*corresponding author email: ingo.ott@tu-braunschweig.de

Table of contents

Table S1. Results of elemental analysis of the insoluble precipitate S3
Figure S1. ^{1}H NMR 2D (EXSY) spectra of **4a** recorded in DMSO-d_6 at T = 300 K. S3
Figure S2. ^{1}H NMR 2D (EXSY) spectra of **4b** recorded in DMSO-d_6 at T = 300 K. S4
Figure S3. ^{1}H NMR 2D (EXSY) spectra of **4b** recorded in DMSO-d_6 at T = 300 K. S5
Figure S4. Conductivity Λ_M (S cm^2 mol^{-1}) in 0.1 to 1.0 mM solutions of **1a-1c** in DMSO S5
Figure S5. Conductivity Λ_M (S cm^2 mol^{-1}) in 0.1 to 1.0 mM solutions of **4a-4c** in DMSO S6
Crystal Structure Determinations S6
Table S2. Crystallographic data and structure refinement details S7
Figure S6. The tube-shaped polymer of compound **1c** S8
Figure S7. The asymmetric unit of compound **6b** in the crystal S8
Figure S8. The ribbon polymer of compound **6a** S8
Table S3. Selected bond lengths [Å] and angles [°] for compound **1c** S9
Table S4. Selected bond lengths [Å] and angles [°] for compound **6a** S9
Table S5. Selected bond lengths [Å] and angles [°] for compound **6b** S10
Figure S9. Degradation of silver complexes after dissolution and after 24 h S10
Figure S10. NTU of silver complexes at the highest tested concentration (50 µM) S11
Figure S11. Solubility curve of silver nitrate ($AgNO_3$) in a DMSO/PBS (0.2 v%) mixture S11
Table S6. Mean EC_{50} values [µM] for antibacterial activity S12
Figure S12. The activity of selected compounds in different media against *E.coli* S13
Figure S13. Inhibition of biofilm formation of *Pseudomonas aeruginosa* by the tested silver halido NHCs at various concentrations in µg/ml S13
Table S7. Composition of culture media used in antibacterial and antibiofilm assays S14
Figure S14. Cytotoxic effect of (NHC)AgI complexes at 100 µM against almost confluent cell layers Caco-2 cells S14
Figures S15-S65: ^{1}H-NMR, ^{13}C-NMR, MS spectra S15
Reference S40

Table S1. Results of elemental analysis of the insoluble precipitate left from the synthesis of silver iodido NHCs. The values given (%) are average values from two independent determinations

	**1c**·AgI		**2c**·AgI		**3c**·AgI		**4c**·AgI	
	Theor.	Calc.	Theor.	Calc.	Theor.	Calc.	Theor.	Calc.
N	6.07	5.44	5.94	5.55	5.83	5.52	5.70	4.49
C	24.27	23.30	25.49	24.87	26.66	25.95	26.08	21.11
H	1.89	1.60	2.14	1.93	2.38	2.25	2.33	1.54

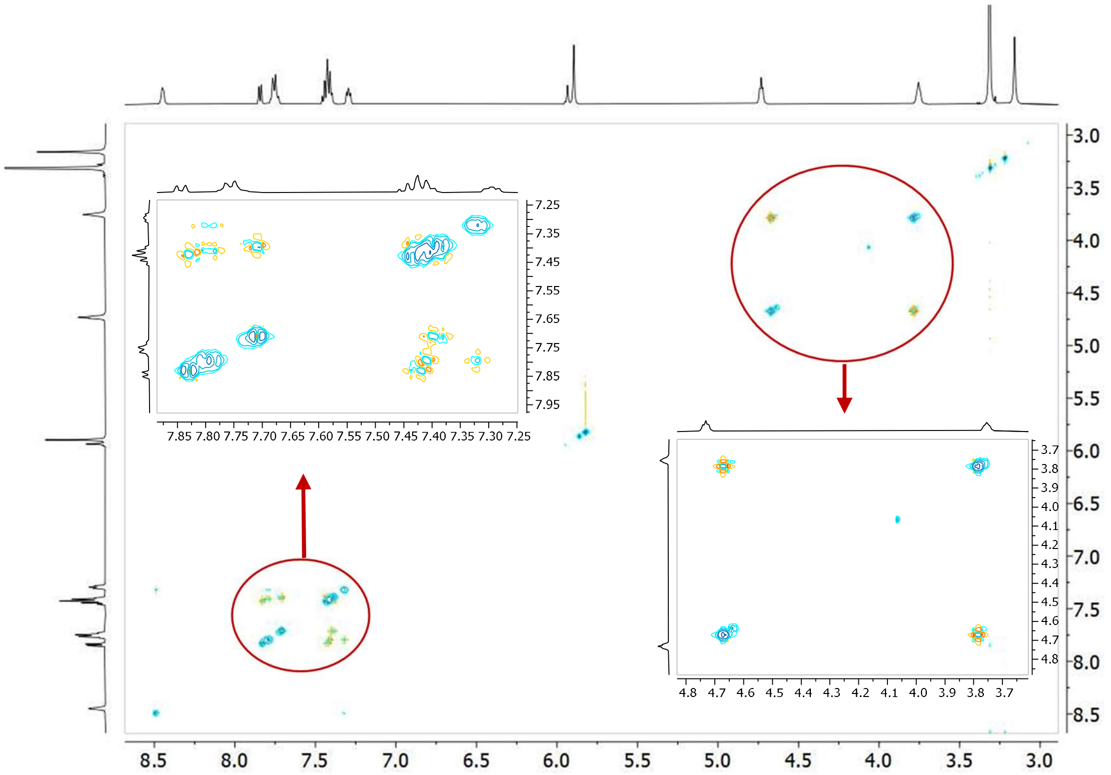

Figure S1. ^{1}H NMR 2D (EXSY) spectra of **4a** recorded in DMSO-d_6 at T = 300 K. The mixing time was 5 ms. Positive cross peaks of aromatic protons and protons of the methoxyethyl side chain are marked with red circles.

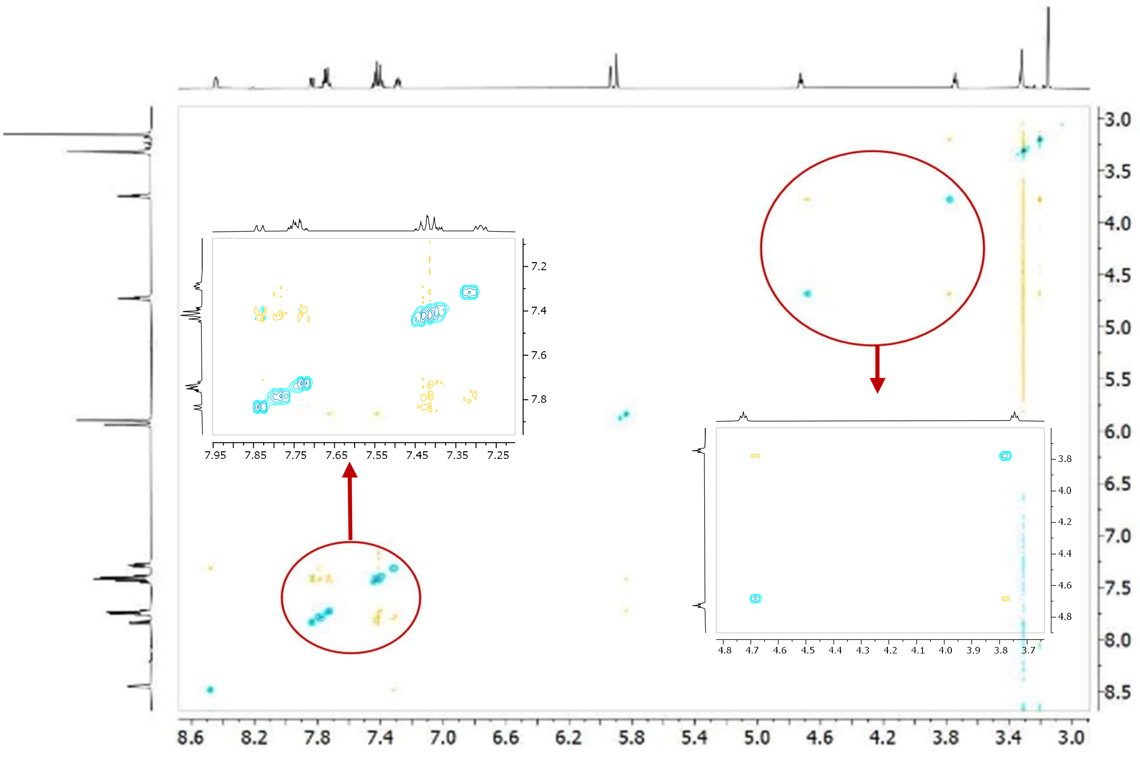

Figure S2. ^{1}H NMR 2D (EXSY) spectra of **4b** recorded in DMSO-d_6 at T = 300 K. The mixing time was 5 ms. Positive cross peaks of aromatic protons and protons of the methoxyethyl side chain are marked with red circles.

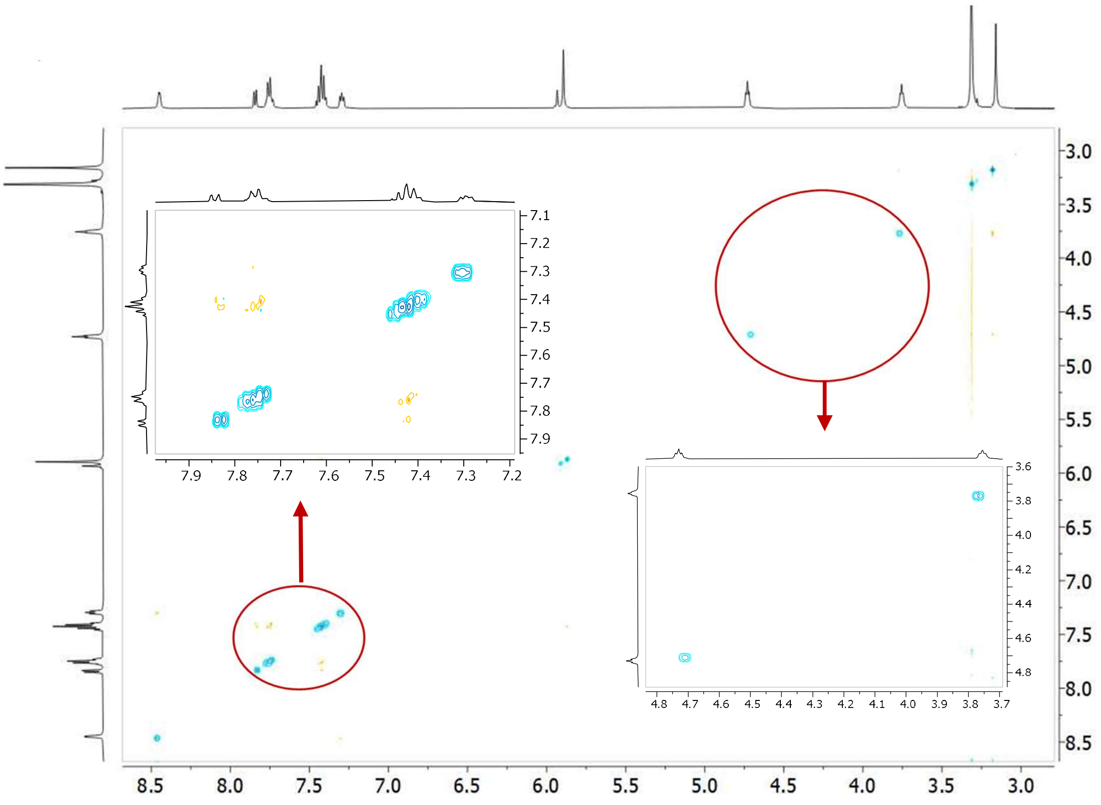

Figure S3. ^{1}H NMR 2D (EXSY) spectra of **4c** recorded in DMSO-d_6 at T = 300 K. The mixing time was 5 ms. Positive cross peaks of aromatic protons and protons of the methoxyethyl side chain are marked with red circles.

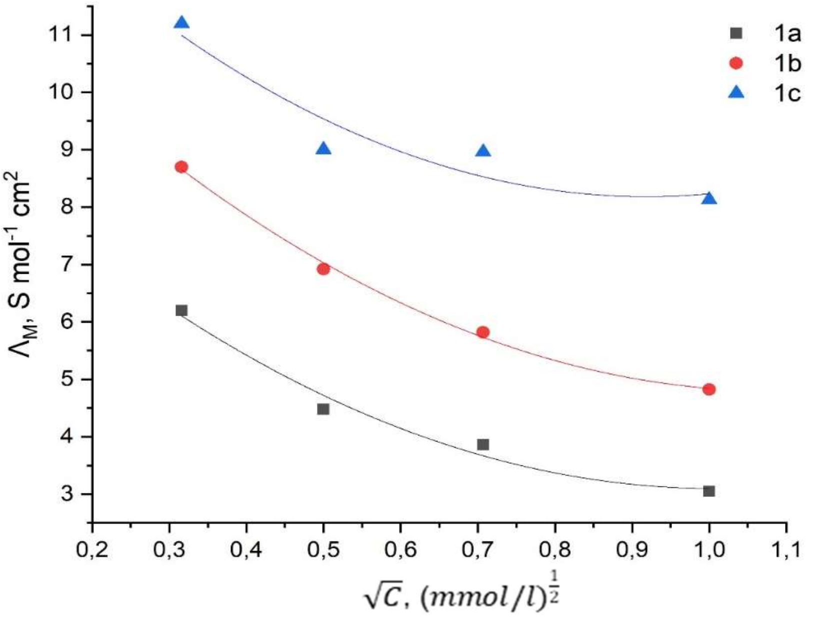

Figure S4. Conductivity Λ_M (S cm^2 mol^{-1}) in 0.1 to 1.0 mM solutions of **1a-1c** in DMSO, plotted against the square root of the concentration

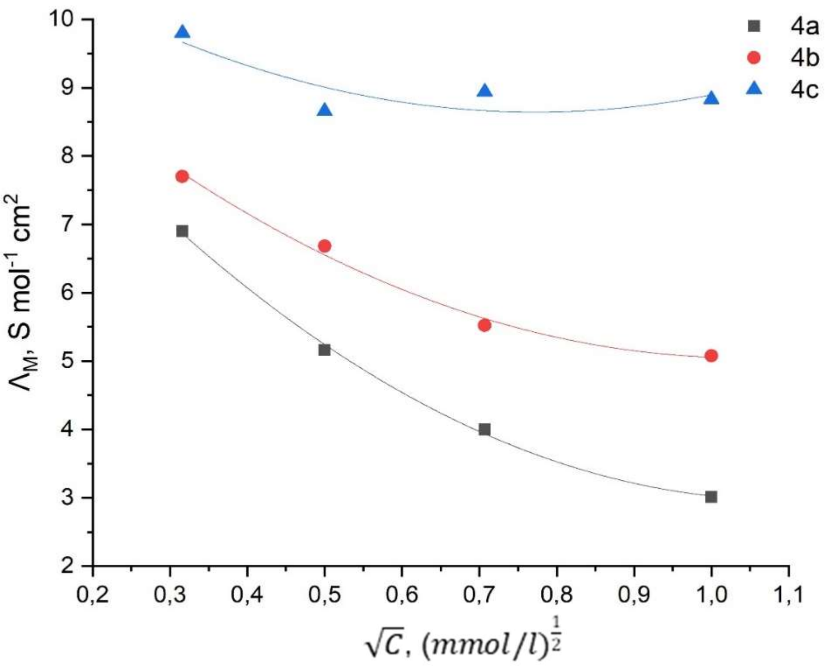

Figure S5. Conductivity Λ_M (S cm^2 mol^{-1}) in 0.1 to 1.0 mM solutions of **4a**-**4c** in DMSO, plotted against the square root of the concentration

Crystal Structure Determinations

Crystals were mounted in inert oil on Hampton loops and transferred to the cold gas stream of the appropriate Rigaku/OD XtaLAB Synergy diffractometer. Mirror-focussed Cu-*K*α radiation was employed for the intensity measurements of **1c** (because the crystals were small thin needles; despite the high absorption coefficient, the structure is of good quality) and mirror-focussed Mo-*K*α radiation for **6a** and **6b**. Absorption corrections were implemented on the basis of multi-scans. The structures were refined anisotropically on F^2 using the program SHELXL-2018.[1] Hydrogen atoms were included using rigid methyl groups or a riding model starting from calculated positions.

Exceptions/special details: The data were of sufficient quality to distinguish the C and N atoms of the pyridyl rings. For **1c**, an extinction correction was performed; this led to an extinction parameter of 0.000235(18). For **6b**, a non-standard cell was employed ($\gamma > 90°$ rather than $\gamma < 90°$) to facilitate comparison with the isotypic structure **6a**.

Crystallographic data are summarized in Table S2. For polymeric structures, the definition of the formula unit is to some extent arbitrary; formal molecular weights and *Z* values are based on the formula units given in the Table. Selected bond lengths and angles are given in Tables

S3–S5. In the Figures, all ellipsoids correspond to 50% probability levels; hydrogen atoms are omitted from the packing diagrams.

Additionally, complete data have been deposited with the Cambridge Crystallographic Data Centre under the numbers CCDC 2322861, 2322863 and 2322864. Copies of the data can be obtained free of charge from www.ccdc.cam.ac.uk/data_request/cif.

Table S2. Crystallographic data and structure refinement details

Compound	**1c**	**6a**	**6b**
CCDC number	2322861	2322863	2322864
Formula	$C_{14}H_{13}Ag_2I_2N_3$	$C_{38}H_{32}Ag_2Cl_2N_8$	$C_{38}H_{32}Ag_2Br_2N_8$
M_r	692.81	887.35	976.27
Cryst. size (mm)	0.10 x 0.02 x 0.02	0.2 x 0.15 x 0.12	0.15 x 0.1 x 0.05
Crystal system	monoclinic	triclinic	triclinic
Space group	$P2_1/n$	P(-1)	P(-1)
Temperature (°C)	-173	-173	-173
a (Å)	4.65326(6)	9.0484(3)	9.17460(17)
b (Å)	27.8329(3)	9.7891(2)	9.8502(3)
c (Å)	12.84431(14)	10.1025(3)	10.1748(3)
α (°)	90	70.265(2)	69.988(3)
β (°)	98.0236(10)	87.529(2)	87.7383(18)
γ (°)	90	89.721(2)	90.4055(18)
V (Å^3)	1647.23	841.45	863.11
Z	4	1	1
D_x (Mg m^{-3})	2.794	1.751	1.878
λ (Å)	1.54184	0.71073	0.71073
μ (mm^{-1})	48.5	1.37	3.49
Transmissions	0.077 – 1.000	0.934 – 1.000	0.805 – 1.000
F(000)	1272	444	480
$2\theta_{max}$	149.9	89.9	82.6
Refl. measured	67677	137910	81422
Refl. indep.	3487	13835	166987
R_{int}	0.048	0.024	0.034
Parameters	192	226	226
$wR(F^2$, all refl.)	0.055	0.056	0.045
$R(F, >4\sigma(F))$	0.021	0.019	0.020
S	1.14	1.06	1.09
Max. Δp (e Å^{-3})	1.09, –0.82	2.62, –0.98	1.72, –0.98

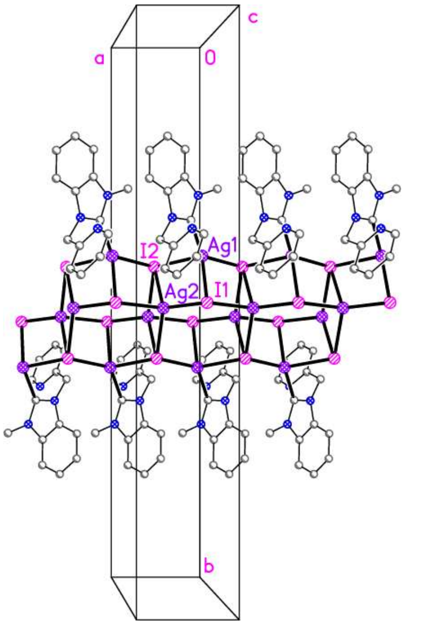

Figure S6. The tube-shaped polymer of compound **1c**, which propagates parallel to the *a* axis

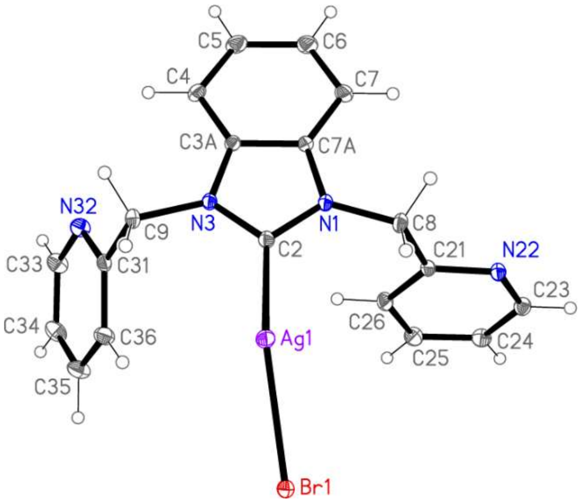

Figure S7. The asymmetric unit of compound **6b** in the crystal. The polymeric structure is strictly analogous to that of the isotypic compound **6a**

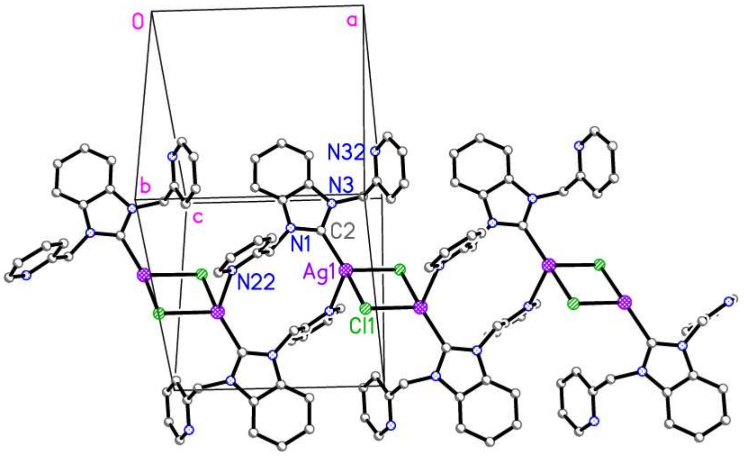

Figure S8. The ribbon polymer of compound **6a**, which propagates parallel to the *a* axis

Table S3. Selected bond lengths [Å] and angles [°] for compound **1c**

Ag(1)-C(2)	2.156(3)	Ag(2)-I(1)	2.7815(3)
Ag(1)-I(2)#1	2.8165(3)	Ag(2)-I(2)#2	2.8547(3)
Ag(1)-I(1)	2.9512(3)	Ag(2)-I(1)#3	2.8840(3)
Ag(1)-I(2)	3.1219(4)	Ag(2)-I(2)	2.8935(3)
C(2)-Ag(1)-I(2)#1	131.09(9)	Ag(2)-I(1)-Ag(2)#1	110.424(11)
C(2)-Ag(1)-I(1)	115.92(9)	Ag(2)-I(1)-Ag(1)	71.731(9)
I(2)#1-Ag(1)-I(1)	97.883(9)	Ag(2)#1-I(1)-Ag(1)	80.617(9)
C(2)-Ag(1)-I(2)	101.31(9)	Ag(1)#3-I(2)-Ag(2)#2	107.531(10)
I(2)#1-Ag(1)-I(2)	103.061(10)	Ag(1)#3-I(2)-Ag(2)	82.763(9)
I(1)-Ag(1)-I(2)	104.620(10)	Ag(2)#2-I(2)-Ag(2)	64.077(9)
I(1)-Ag(2)-I(2)#2	113.875(11)	Ag(1)#3-I(2)-Ag(1)	103.061(10)
I(1)-Ag(2)-I(1)#3	110.425(11)	Ag(2)#2-I(2)-Ag(1)	117.704(10)
I(2)#2-Ag(2)-I(1)#3	100.443(10)	Ag(2)-I(2)-Ag(1)	67.825(8)
I(1)-Ag(2)-I(2)	115.772(10)	N(1)-C(2)-Ag(1)	129.2(2)
I(2)#2-Ag(2)-I(2)	115.923(9)	N(3)-C(2)-Ag(1)	125.4(2)
I(1)#3-Ag(2)-I(2)	97.684(10)		

Symmetry transformations used to generate equivalent atoms:
#1 *x*-1, *y*, *z*; #2 -*x*+1, -*y*+1, -*z*; #3 *x*+1, *y*, *z*.

Table S4. Selected bond lengths [Å] and angles [°] for compound **6a**

Ag(1)-C(2)	2.1392(6)	Ag(1)-N(22)#1	2.5283(5)
Ag(1)-Cl(1)	2.52199(17)	Ag(1)-Cl(1)#2	2.79621(17)
C(2)-Ag(1)-Cl(1)	148.506(16)	Ag(1)-Cl(1)-Ag(1)#2	88.456(5)
C(2)-Ag(1)-N(22)#1	109.98(2)	N(3)-C(2)-Ag(1)	127.86(4)
Cl(1)-Ag(1)-N(22)#1	91.435(14)	N(1)-C(2)-Ag(1)	126.95(4)
C(2)-Ag(1)-Cl(1)#2	111.196(16)	N(22)-C(21)-C(26)	122.31(6)
Cl(1)-Ag(1)-Cl(1)#2	91.544(6)	C(23)-N(22)-Ag(1)#1	114.13(4)
N(22)#1-Ag(1)-Cl(1)#2	88.489(13)	C(21)-N(22)-Ag(1)#1	124.05(4)

Symmetry transformations used to generate equivalent atoms:
#1 -*x*+1, -*y*+1, -*z*+2; #2 -*x*+2, -*y*+1, -*z*+2.

Table S5. Selected bond lengths [Å] and angles [°] for compound **6b**

Ag(1)-C(2)	2.1457(7)	Ag(1)-N(22)#1	2.5283(5)
Ag(1)-N(22)#1	2.5340(6)	Ag(1)-Cl(1)#2	2.79621(17)
C(2)-Ag(1)-N(22)#1	111.26(2)	Ag(1)-Br(1)-Ag(1)#2	87.466(4)
C(2)-Ag(1)-Br(1)	144.66(2)	N(3)-C(2)-Ag(1)	128.21(5)
N(22)#1-Ag(1)-Br(1)	92.531(16)	N(1)-C(2)-Ag(1)	126.62(5)
C(2)-Ag(1)-Br(1)#2	112.287(19)	C(23)-N(22)-Ag(1)#1	113.42(5)
N(22)#1-Ag(1)-Br(1)#	290.307(15)	C(21)-N(22)-Ag(1)#1	124.91(5)
Br(1)-Ag(1)-Br(1)#2	92.535(4)		

Symmetry transformations used to generate equivalent atoms:
#1 -*x*+1, -*y*+1, -*z*+2; #2 -*x*+2, -*y*+1, -*z*+2.

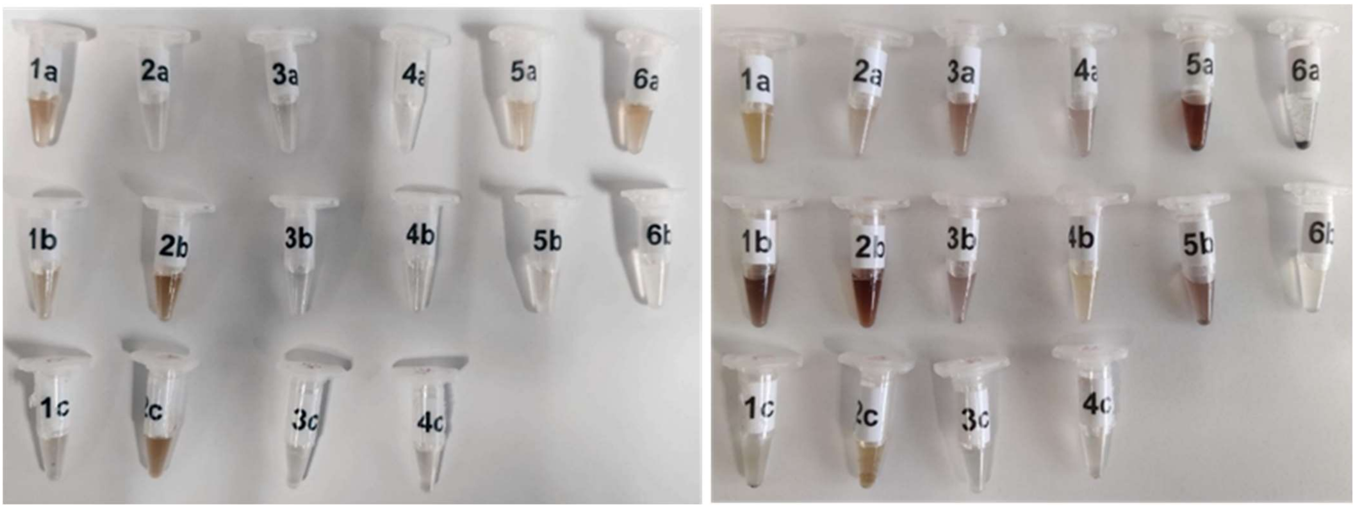

Figure S9. Degradation of silver complexes immediately after dissolution (left) and after 24 h (right)

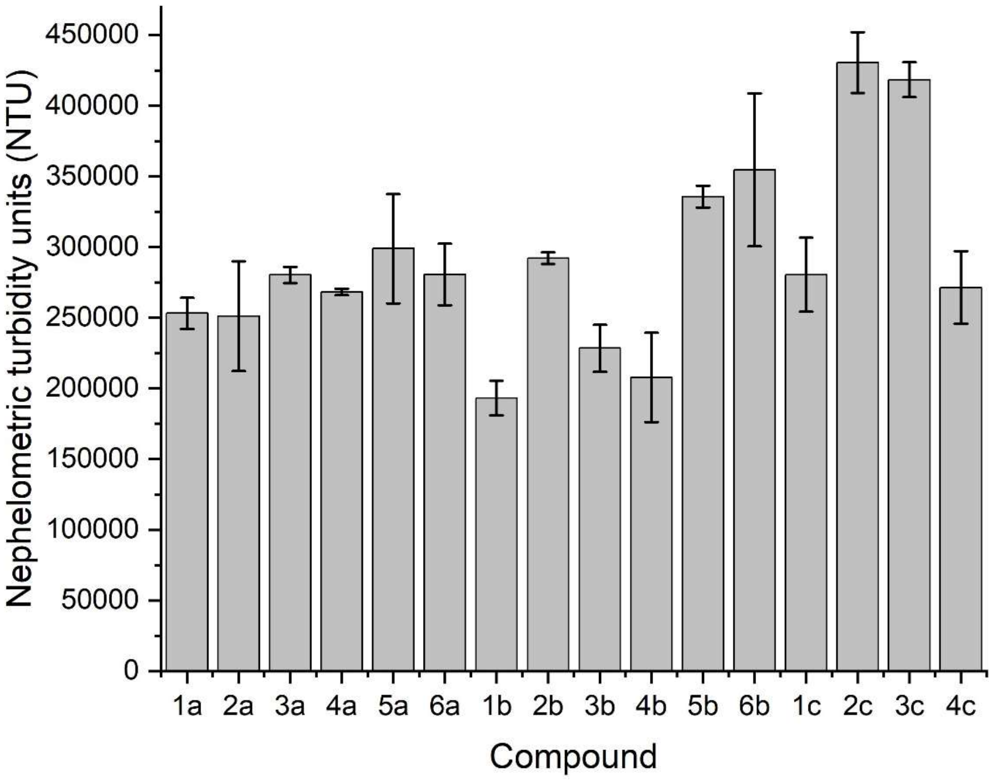

Figure S10. NTU of silver complexes at the highest test concentration (50 μM)

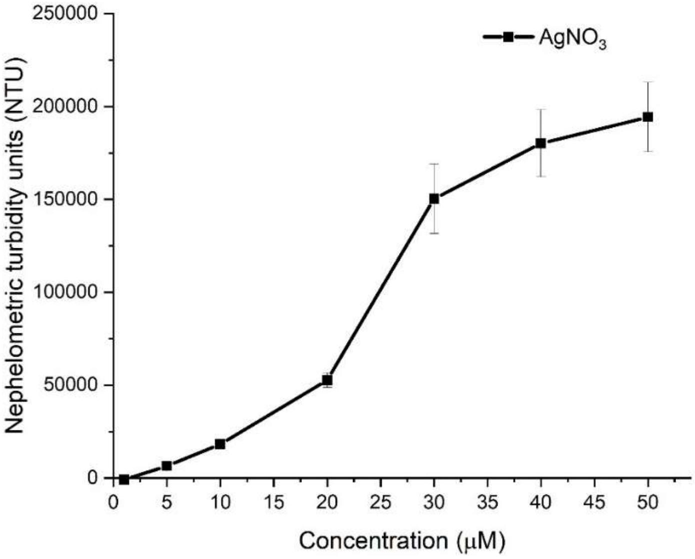

Figure S11. Solubility curve of silver nitrate ($AgNO_3$) in a DMSO/PBS (0.2 v%) mixture; the turbidity (NTU) is plotted against the concentration

Table S6. Mean EC_{50} values of silver complexes and references in μM ± standard error (n=3).

	Gram-negative bacteria				Gram-positive bacteria	
	E.coli	*P.aeruginosa*	*K.pneumoniae*	*A. baumannii*	*MRSA*	*E.faecium*
**1a(Cl)**	53.3 ± 8.6	5.7 ± 0.8	8.5 ± 1.2	1.7 ± 0.4	146 ± 2.8	145 ± 2.3
**1b(Br)**	71.3 ± 6.7	10.9 ± 3.9	10.0 ± 2.2	2.6 ± 0.3	134 ± 14	129 ± 2.8
**1c(I)**	6.4 ± 1.1	5.0 ± 0.5	2.0 ± 0.2	4.1 ± 0.4	> 140	> 140
**2a(Cl)**	33.3 ± 4.7	6.4 ± 0.5	12.0 ± 0.9	2.4 ± 0.1	98.5 ± 0.8	132 ± 6.8
**2b(Br)**	35.6 ± 9.4	6.9 ± 0.5	9.0 ± 1.0	2.2 ± 0.3	88.5 ± 1.6	128 ± 0.9
**2c(I)**	12.8 ± 1.1	14.2 ± 0.8	5.9 ± 1.7	2.9 ± 0.5	> 136	>136
**3a(Cl)**	35.3 ± 1.1	39.1 ± 8.8	9.8 ± 2.4	1.8 ± 0.3	88.6 ± 1.6	139 ± 0.4
**3b(Br)**	28.7 ± 3.6	30.0 ± 6.9	8.6 ± 0.2	0.53 ± 0.14	78.8 ± 0.4	123 ± 0.4
**3c(I)**	8.8 ± 2.5	6.0 ± 1.0	2.3 ± 0.3	1.9 ± 0.1	> 132	60.9 ± 0.1
**4a(Cl)**	31.1 ± 1.8	9.6 ± 0.3	5.2 ± 0.1	1.9 ± 0.4	85.7 ± 0.6	123 ± 6.1
**4b(Br)**	59.2 ± 4.3	8.6 ± 1.1	9.8 ± 1.0	2.1 ± 0.1	78.3 ± 2.0	120 ± 0.8
**4c (I)**	14.4 ± 2.7	4.9 ± 1.2	2.0 ± 0.0	1.9 ± 0.1	>128	>128
**5a (Cl)**	35.1 ± 1.1	16.0 ± 1.7	8.5 ± 1.0	1.7 ± 0.0	82.9 ± 1.5	119 ± 3.6
**5b (Br)**	27.1 ± 1.2	28.2 ± 2.7	9.5 ± 0.3	0.72 ± 0.15	75.5 ± 1.4	111 ± 0.2
**6a (Cl)**	38.1 ± 6.9	33.6 ± 5.8	5.9 ± 0.2	1.6 ± 0.1	91.3 ± 0.6	133 ± 0.6
**6b (Br)**	14.6 ± 1.0	13.5 ± 3.7	6.4 ± 0.2	0.47 ± 0.02	90.7 ± 4.5	105 ± 0.1
**$AgNO_3$**	4.87 ± 0.1	4.12 ± 0.40	4.41 ± 0.50	4.16 ± 0.40	98.9 ± 6.4	75.9 ± 5.7
**SSD**	2.37 ± 0.0	2.50 ± 0.05	2.77 ± 0.1	2.34 ± 0.0	104 ± 1.0	82.4 ± 14.2
**L3a**	>250	>250	>250	>250	>250	>250
**L3b**	>250	>250	>250	>250	>250	≥250
**L3c**	>250	≥250	>250	>250	>250	>250

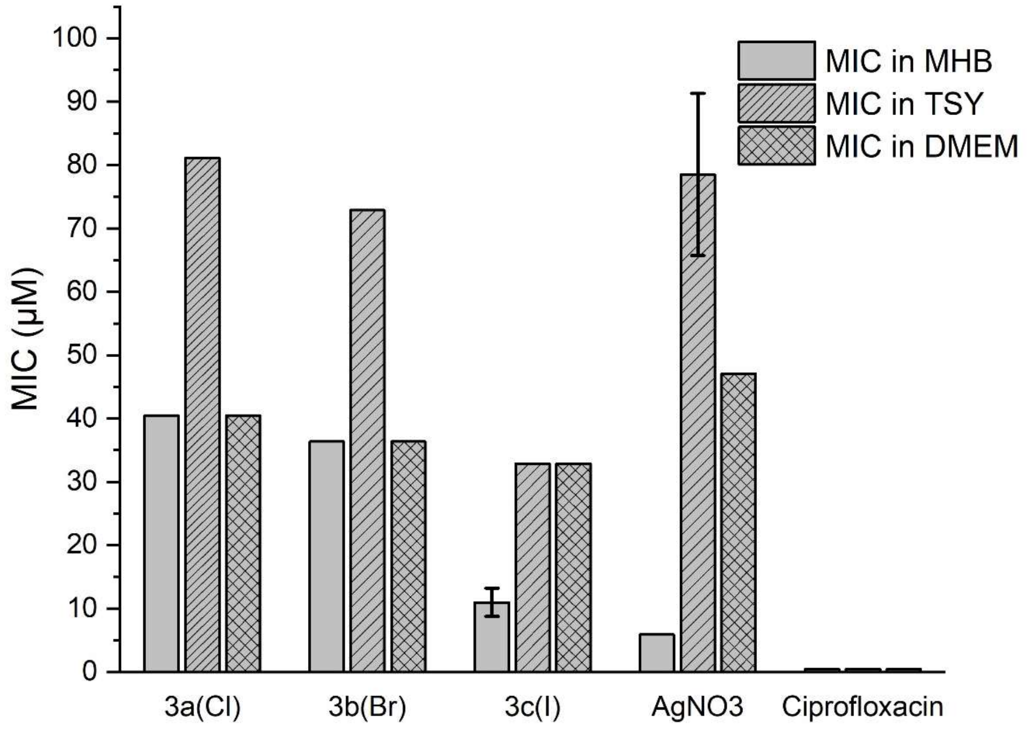

Figure S12. The activity of selected compounds in different media against E.coli (n = 3)

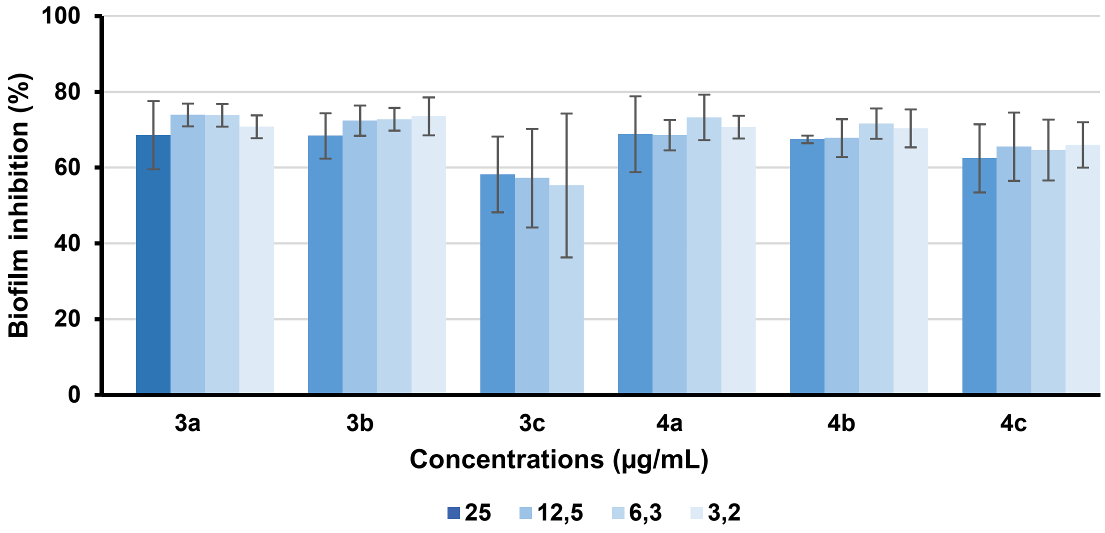

Figure S13. Inhibition of biofilm formation of *Pseudomonas aeruginosa* by the tested silver halido NHCs at various concentrations in µg/ml

Table S7. Composition of culture media used in antibacterial and antibiofilm assays

Medium	Composition for 1L solution	pH
MHB (Müller-Hinton broth)	17.5 g acid hydrolysate of casein, 3g beef extract, 1.5 g starch	7.4
TSY (Tryptic soy yeast broth)	17 g Tryptone, 2.5 g K_2HPO_4, 2.5 g Glucose, 5 g NaCl, 3 g soya peptone, 3 g yeast extract	7.0-7.2
LB (Lysogeny broth)	10 g Tryptone, 5 g yeast extract, 5 g NaCl, pH 7.0-7.4	7.4
M63 broth	2 g $(NH_4)_2SO_4$, 13.6 g KH_2PO_4 supplemented with 0.2% Glucose, 120 mg $MgSO_4$, casamino acids (CAA,0.5%), citric acid (0.4%), glutamic acid (monosodium salt,0.4%) or 0.8 mg $FeSO_4{\cdot}7H_20$	7.0
PBS (Phosphate-buffered saline)	8 g NaCl, 0.2 g KCl, 1.44 g Na_2HPO_4, 0.24 g KH_2PO_4	7.4

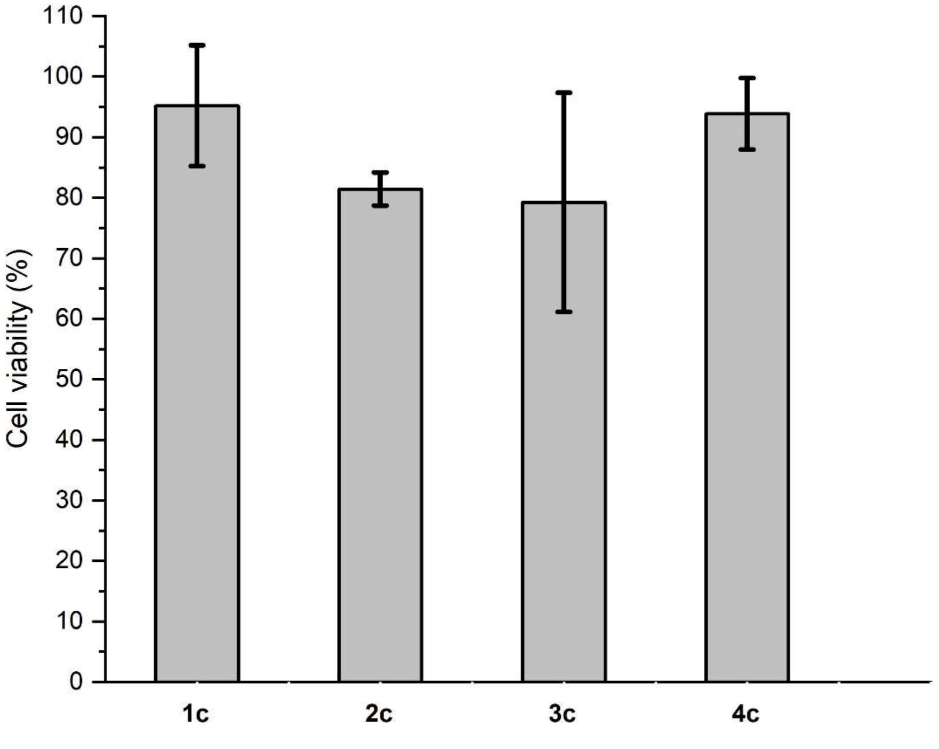

Figure S14. Cytotoxic effect of (NHC)AgI complexes at 100 μM against almost confluent cell layers Caco-2 cells (as % control of untreated cells)

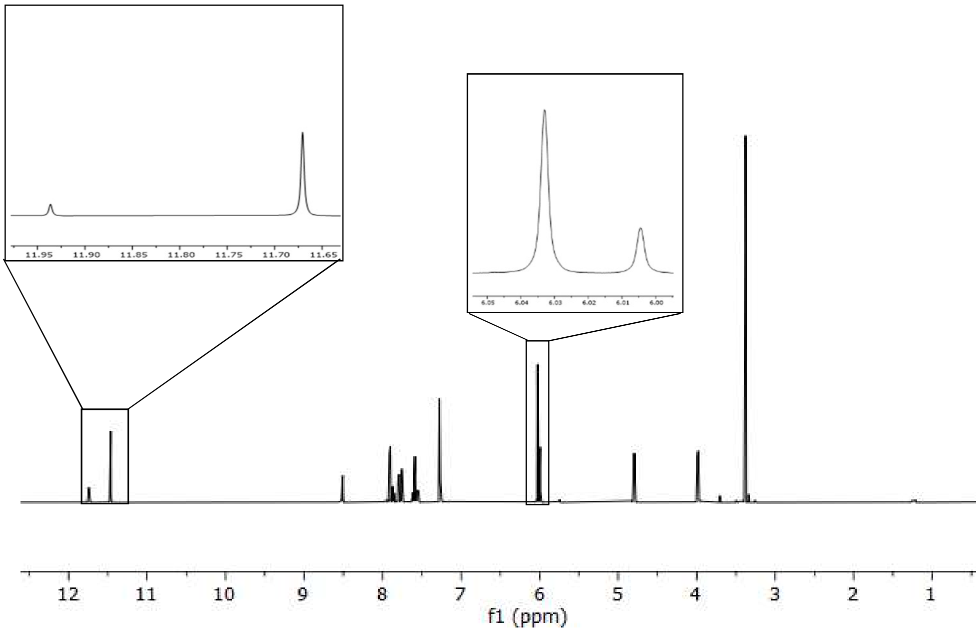

Figure S15. **L4a**, ^{1}H-NMR (600 MHz, $CDCl_3$-*d*$_1$) δ = 11.67 (s, BeIm-H2, 1H), 8.51 (ddd, $J_{H,H}$ = 4.8, 1.8, 0.9 Hz, Py-H5, 1H), 7.97 – 7.89 (m, Py -H2-H3, 2H), 7.82-7.72 (m, BeIm-H4/H7, 2H), 7.64-7.51 (m, BeIm-H4/H7, 2H), 7.29 – 7.22 (m, Py-H4, 1H), 6.02 (s, Py-***CH₂***, 2H), 4.86-4.78 (m, N-CH_2***CH_2***OCH_3, 2H), 3.99-3.94 (m, N-***CH_2***CH_2OCH_3, 2H), 3.37 (s, N-CH_2CH_2O***CH_3***, 3H)

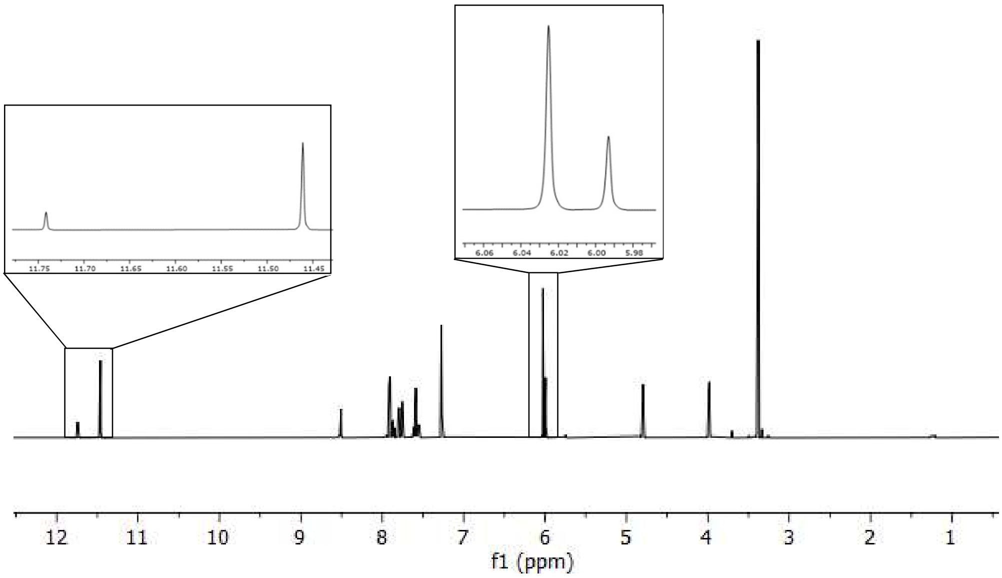

Figure S16. **L4b**, ^{1}H-NMR (600 MHz, $CDCl_3$-*d*$_1$) δ = 11.46 (s, BeIm-H2, 1H), 8.51 (ddd, $J_{H,H}$ = 4.7, 1.8, 0,9 Hz, Py-H5, 1H), 7.97 – 7.81 (m, Py -H2-H3, 2H), 7.82-7.72 (m, BeIm-H4/H7, 2H), 7.64-7.52 (m, BeIm-H4/H7, 2H), 7.29 – 7.24 (m, Py-H4, 1H), 6.03 (s, Py-***CH_2***, 2H), 4.81-4.79 (m, N-CH_2***CH_2***OCH_3, 2H), 4.01-3.95 (m, N-***CH_2***CH_2OCH_3, 2H), 3.38 (s, N-CH_2CH_2O***CH_3***, 3H)

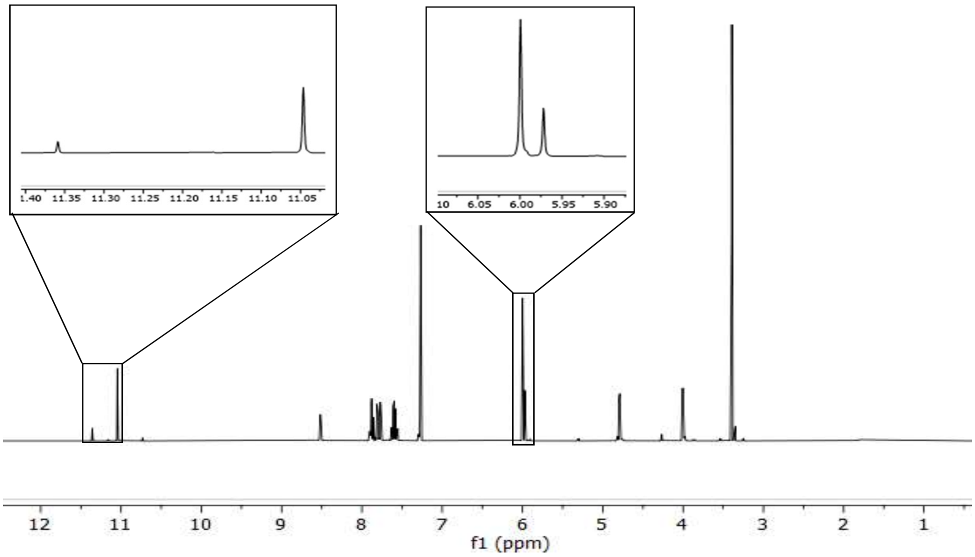

Figure S17. **L4c**, ^{1}H-NMR (500 MHz, CDCl3-d_1) δ = 11.05 (s, BeIm-H2, 1H), 8.52 (ddd, *J*H,H = 4.8, 1.8, 1.0 Hz, Py-H5, 1H), 7.92 – 7.84 (m, Py -H2-H3, 2H), 7.82-7.74 (m, BeIm-H4/H7, 2H), 7.63-7.55 (m, BeIm-H4/H7, 2H), 7.31 – 7.25 (m, Py-H4, 1H), 6.00 (s, Py-***CH_2***, 2H), 4.80-4.78 (m, N-CH2***CH_2***OCH_3, 2H), 4.03-3.96 (m, N-***CH_2***CH_2OCH_3, 2H), 3.39 (s, N-CH_2CH_2O***CH_3***, 3H)

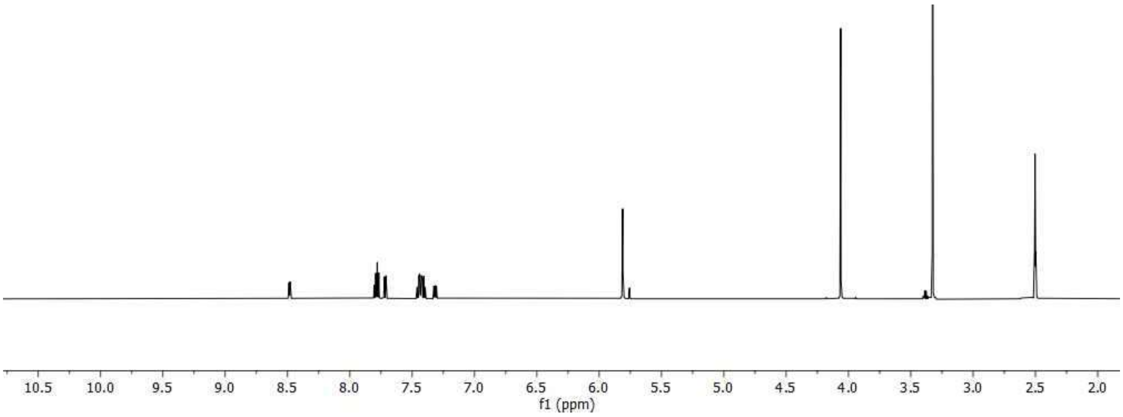

Figure S18. **1a**, ^{1}H-NMR (600 MHz, DMSO-d_6) δ = 8.48 (ddd, $J_{H,H}$ = 4.8, 1.8, 0.9 Hz, Py-H5, 1H), 7.83 – 7.75 (m, Py -H2-H3, 2H), 7.72 (dt, $J_{H,H}$ = 7.9, 0.9 Hz, BeIm-H4/H7, 1H), 7.48 – 7.36 (m, BeIm-H4/H7, 3H), 7.32 (ddd, $J_{H,H}$ = 7.6, 4.8, 1.1 Hz, Py-H4, 1H), 5.81 (s, Py-***CH_2***, 2H), 4.06 (s, CH_3, 3H)

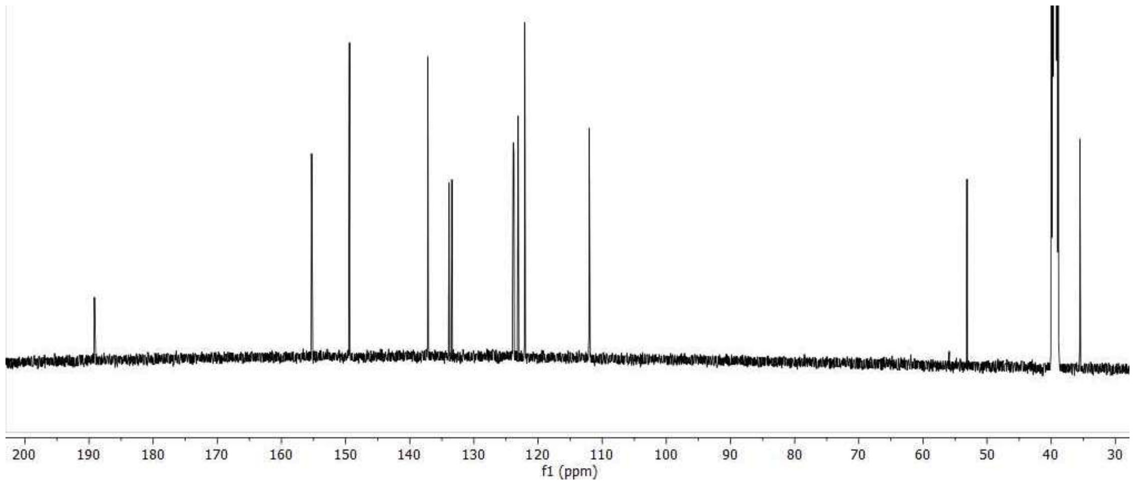

Figure S19. **1a**, ^{13}C-NMR (151 MHz, DMSO-d_6) δ = 189.15 (BeIm-C2) 155.30 (Py-C1), 149.39 (Py-C5), 137.17 (Py-C3), 133.87, 133.43, 123.87, 123.77 (BeIm-C4-C7), 123.10, 122.07 (Py-C2/C4), 112.03, 111.94 (BeIm-C4-C7), 53.13 (Py-***CH_2***), 33.53 (CH_3)

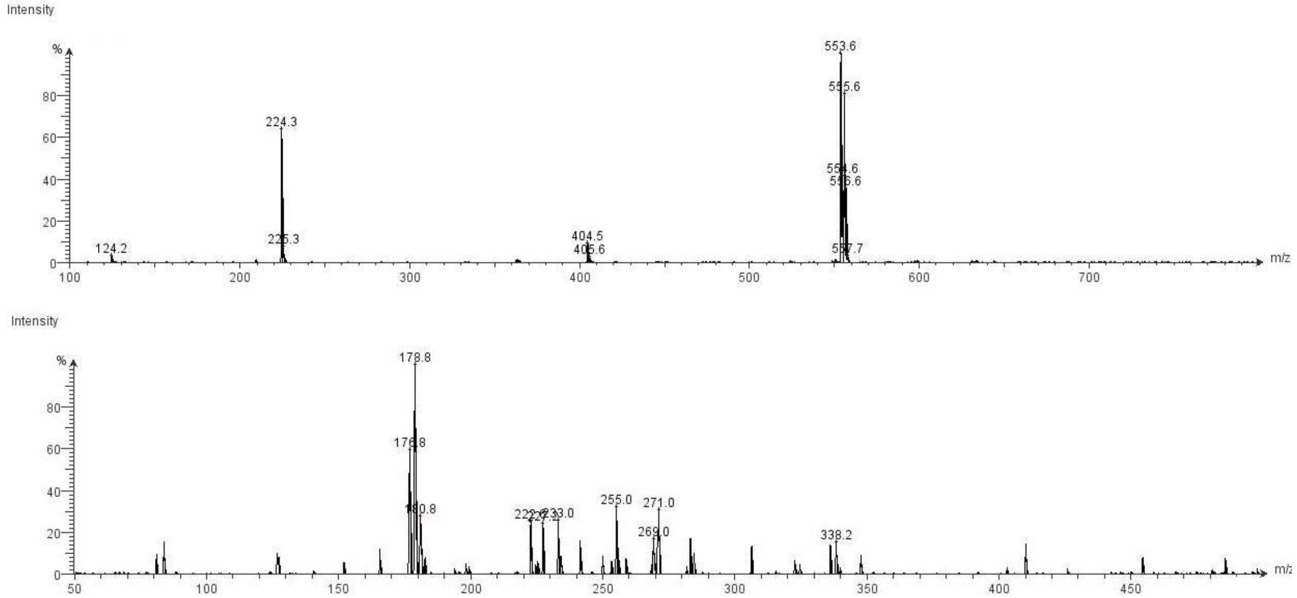

Figure S20. **1a,** positive (top) and negative (bottom) ESI-MS: m/z (ESI+) 555.7 [NHC-Ag-NHC]$^{+}$, 224.3[M-AgCl]$^{+}$; (ESI-): 178.7 [Cl-Ag-Cl]$^{-}$

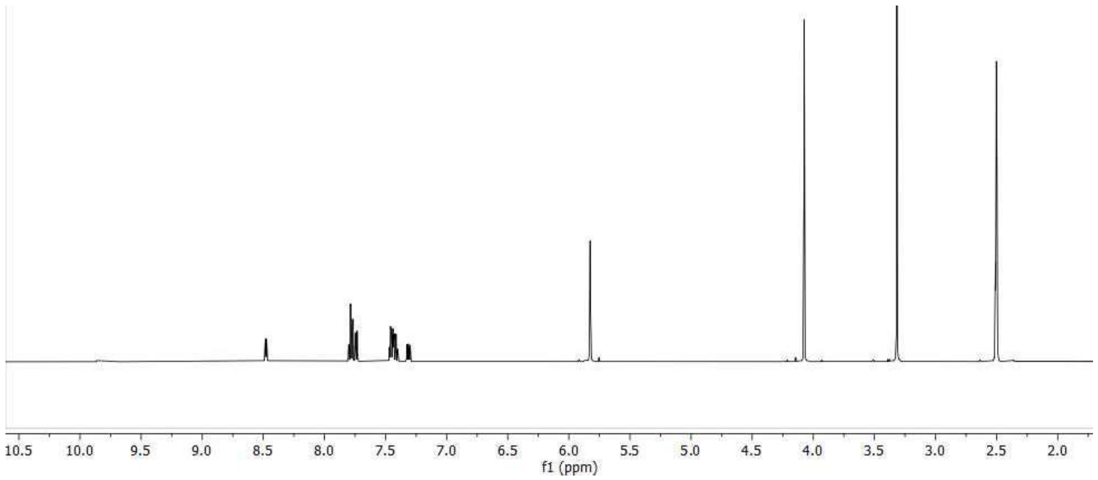

Figure S21. 1b, ^{1}H-NMR (600 MHz, DMSO-d_6) δ = 8.47 (ddd, $J_{H,H}$ = 4.8, 1.8, 1.0 Hz, Py-H5, 1H), 7.78 (td, $J_{H,H}$ = 7.7, 1.8 Hz, Py -H2-H3, 2H), 7.77-7.71 (m, BeIm-H4/H7, 1H), 7.49 - 7.42 (m, BeIm-H4/H7, 2H), 7.45-7.38 (m, BeIm-H4/H7, 1H), 7.31 (ddd, $J_{H,H}$ = 7.6, 4.8, 1.2 Hz, Py-H4, 1H), 5.83 (s, Py-***CH₂***, 2H), 4.07 (s, CH_3, 3H)

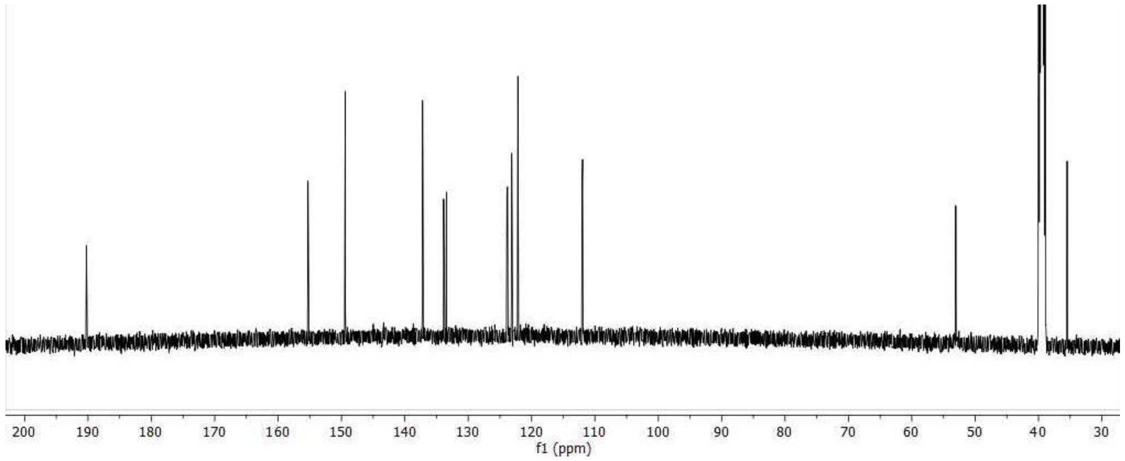

Figure S22. **1b**, ^{13}C-NMR (151 MHz, DMSO-d_6) δ = 190.25 (BeIm-C2), 155.29 (Py-C1), 149.40 (Py-C5), 137.20 (Py-C3), 133.87, 133.43 (BeIm-C4-C7), 123.78, 123.89, 123.13, 122.16 (Py-C2/C4), 112.01, 111.95 (BeIm-C4-C7), 53.06 (Py-***CH_2***), 35.48 (CH_3)

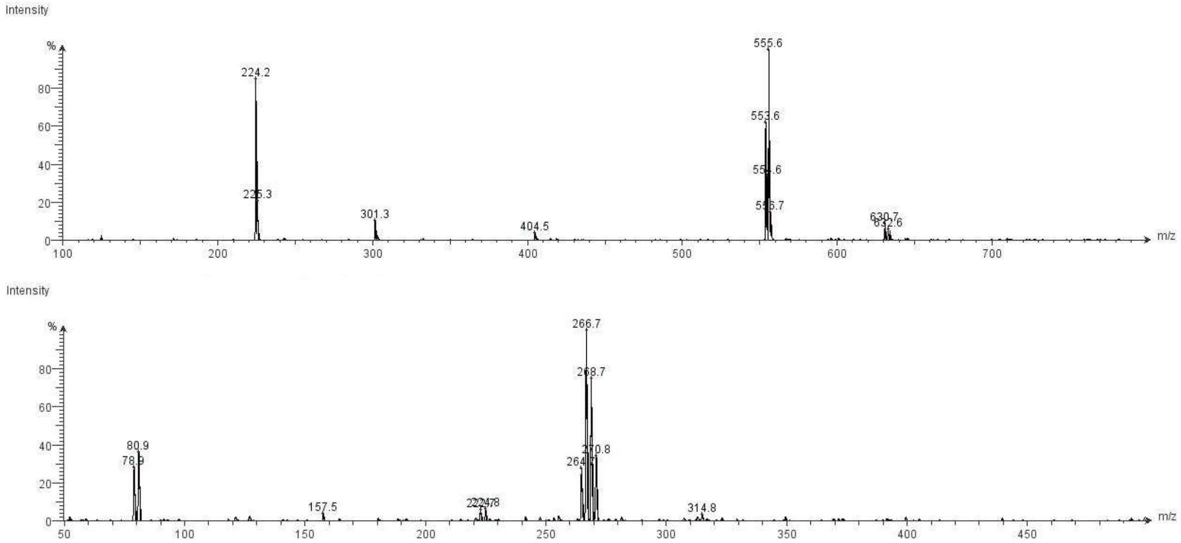

Figure S23. **1b,** positive (top) and negative (bottom) ESI-MS: m/z (ESI+) 555.7 [NHC-Ag-NHC]$^{+}$, 224.3[M-AgBr]$^{+}$; (ESI-): 266.7 [Br-Ag-Br]$^{-}$

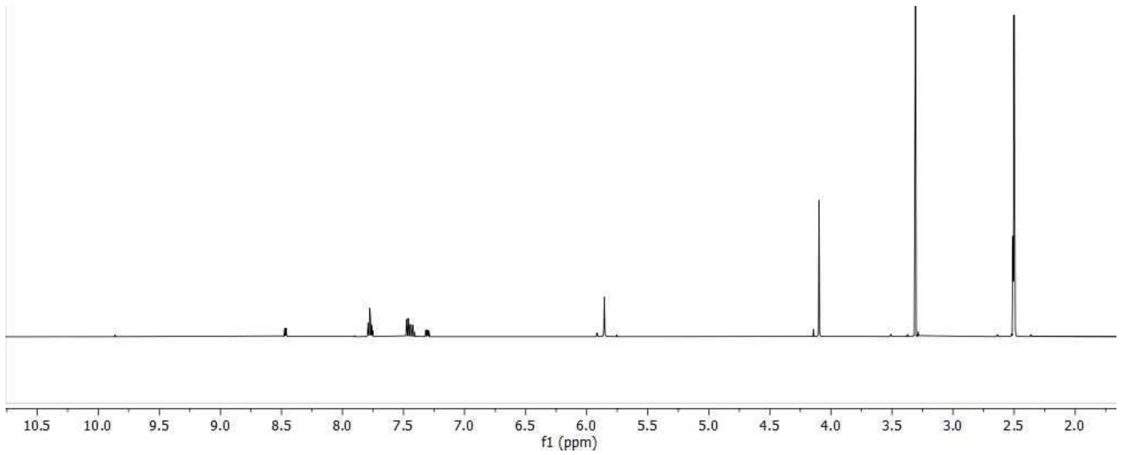

Figure S24. **1c**, ^{1}H-NMR (500 MHz, DMSO-d_6) δ = 8.47 (ddd, $J_{H,H}$ = 4.9, 1.8, 0.9 Hz, Py-H5, 1H), 7.81 – 7.74 (m, Py -H2-H3/BeIm-H4/H7, 3H), 7.50 – 7.44 (m, BeIm-H4/H7, 2H), 7.46, 7.39 (m, BeIm-H4/H7, 1H), 7.30 (ddd, $J_{H,H}$ = 7.6, 4.8, 1.2 Hz, Py-H4, 1H), 5.86 (s, Py-***CH₂***, 2H), 4.10 (s, CH_3, 3H)

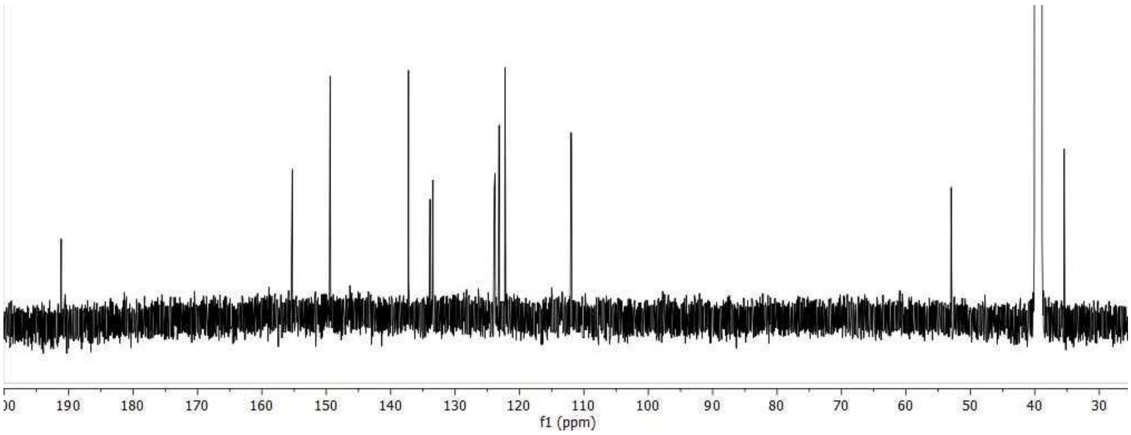

Figure S25. **1c**, ^{13}C-NMR (126 MHz, DMSO-d_6) δ = 191.15 (BeIm-C2), 155.28 (Py-C1), 149.40 (Py-C5), 137.23 (Py-C3), 133.89, 133.43 (BeIm-C4-C7), 123.90, 123.80, 123.15, 122.25 (Py-C2/C4), 111.98, 111.94 (BeIm-C4-C7), 52.98 (Py-***CH_2***), 35.43 (CH_3)

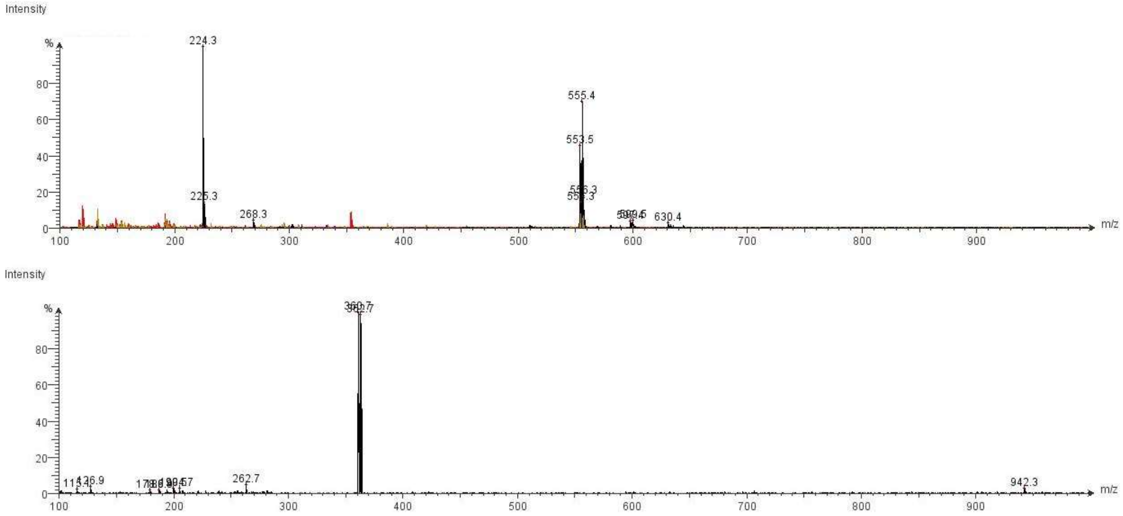

Figure S26. **1c,** positive (top) and negative (bottom) ESI-MS: m/z (ESI+) 555.7 [NHC-Ag-NHC]$^+$, 224.3[M-AgBr]$^+$; (ESI-): 360.7 [I-Ag-I]$^-$

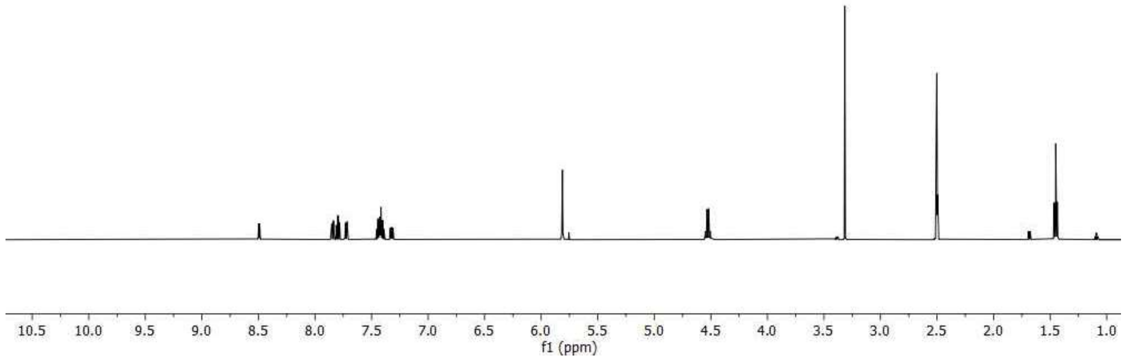

Figure S27. **2a**, ^{1}H-NMR (500 MHz, DMSO-d_6) δ = 8.49 (ddd, $J_{H,H}$ = 4.8, 1.8, 0.9 Hz, Py-H5, 1H), 7.87-7.76 (m, Py -H2-H3, 2H), 7.76-7.70 (m, BeIm-H4/H7, 1H), 7.48 – 7.36 (m, BeIm-H4/H7, 3H), 7.26 (ddd, $J_{H,H}$ = 7.6, 4.8, 1.1 Hz, Py-H4, 1H), 5,81 (s, Py-$\boldsymbol{CH_2}$, 2H), 4.53 (q, $J_{H,H}$ = 7.2 Hz Et-$\boldsymbol{CH_2}$, 2H), 1.45 (t, $J_{H,H}$ = 7.2 Hz, Et-$\boldsymbol{CH_3}$, 3H)

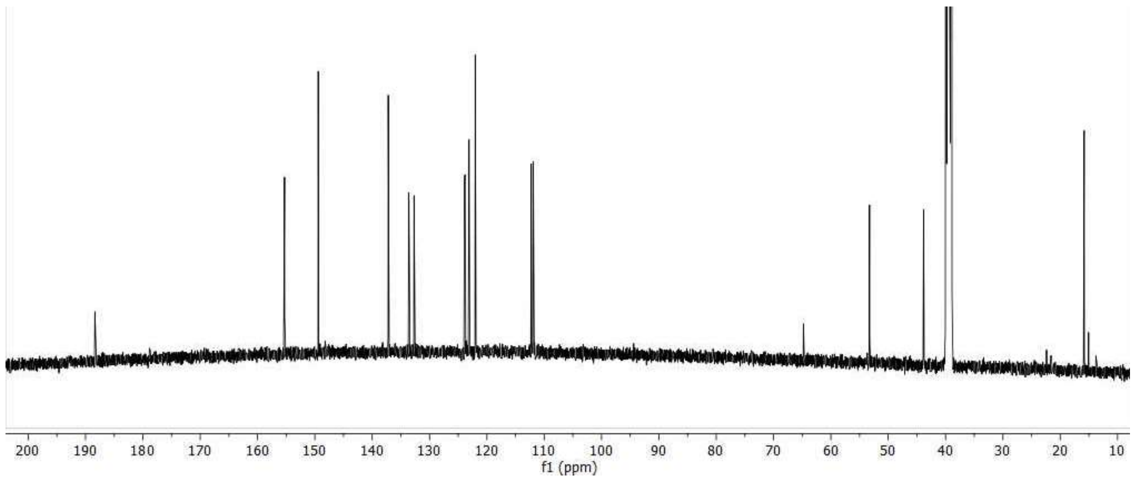

Figure S28. **2a**, ^{13}C-NMR (126 MHz, DMSO-d_6) δ = 188.36 (BeIm-C2), 155.28 (Py-C1), 149.42 (Py-C5), 137.20 (Py-C3), 133.61, 132.70, 123.89, 123.81 (BeIm-C4-C7), 123.11, 122.03 (Py-C2/C4), 112.25, 111.91 (BeIm-C4-C7), 53.26 (Py-$\boldsymbol{CH_2}$), 43.83 (Et-$\boldsymbol{CH_2}$) 15.82 (Et-$\boldsymbol{CH_3}$)

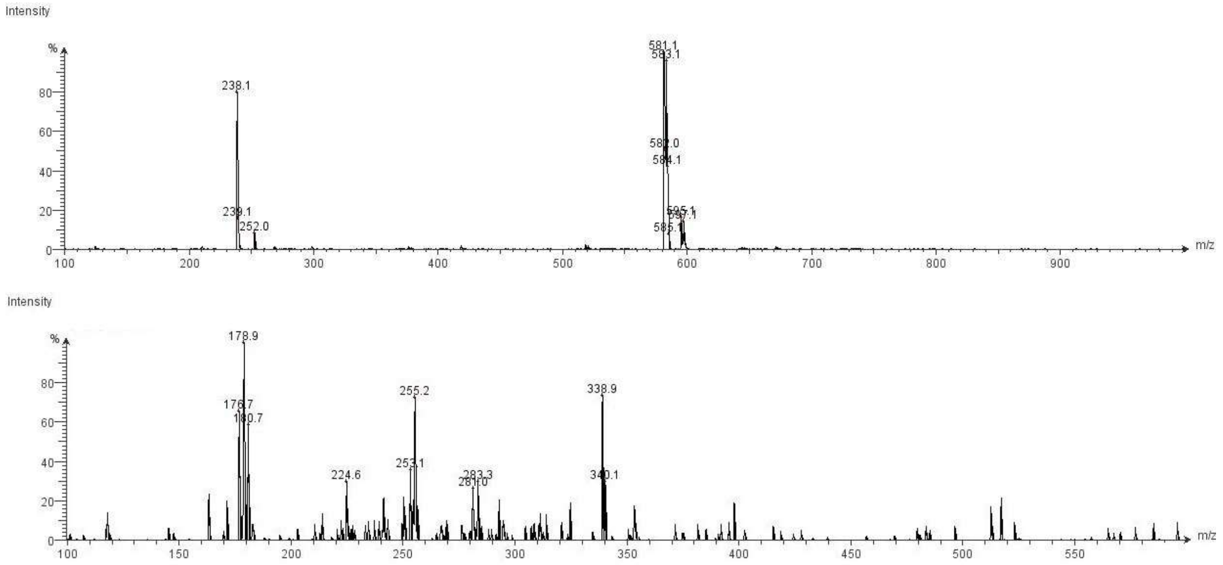

Figure S29. **2a,** positive (top) and negative (bottom) ESI-MS: m/z (ESI+) 580.9 [NHC-Ag-NHC]$^{+}$, 238.0 [M-AgCl]$^{+}$; (ESI-): 178.7 [Cl-Ag-Cl]$^{-}$

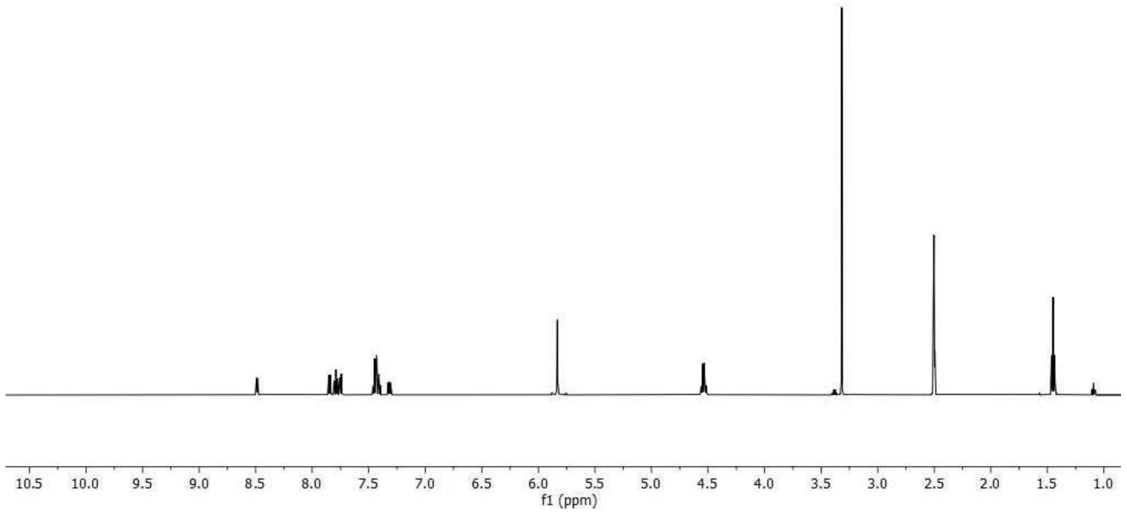

Figure S30. **2b**, ^{1}H-NMR (500 MHz, DMSO-d_6) δ = 8.49 (ddd, $J_{H,H}$ = 4.8, 1.8, 0.9 Hz, Py-H5, 1H), 7.88-7.80 (m, BeIm-H4/H7, 1H), 7.83-7.72 (m, Py -H2-H3, 2H), 7.49 – 7.37 (m, BeIm-H4/H7, 3H), 7.26 (ddd, $J_{H,H}$ = 7.6, 4.9, 1.2 Hz, Py-H4, 1H), 5,83 (s, Py-***CH$_2$***, 2H), 4.54 (q, $J_{H,H}$ = 7.2 Hz Et-***CH$_2$***, 2H), 1.45 (t, $J_{H,H}$ = 7.2 Hz, Et-***CH$_3$***, 3H)

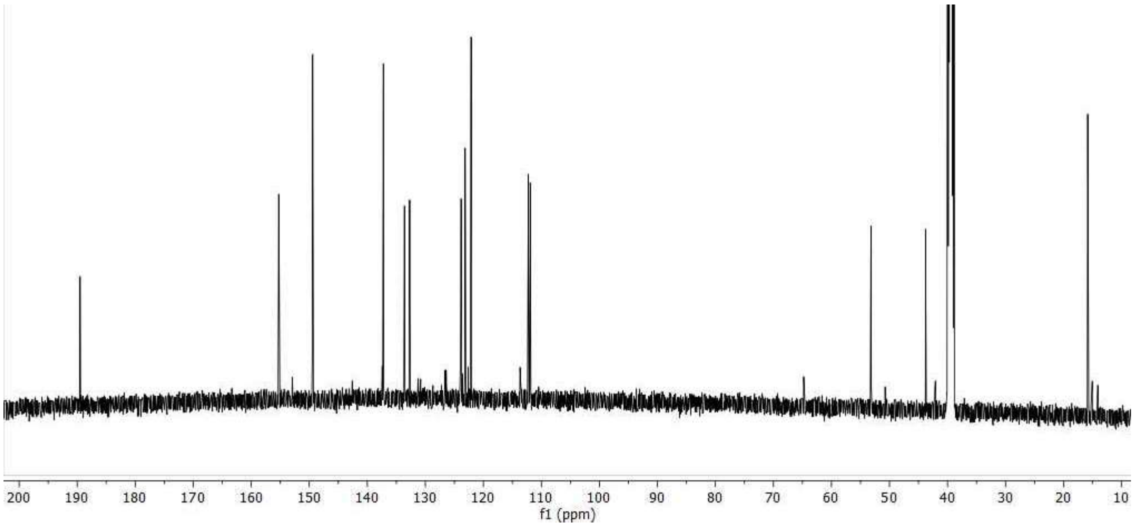

Figure S31. **2b**, ^{13}C-NMR (126 MHz, DMSO-d_6) δ = 189.55 (BeIm-C2), 155.27 (Py-C1), 149.42 (Py-C5), 137.23 (Py-C3), 133.60, 132.70, 123.91, 123.83 (BeIm-C4-C7), 123.14, 122.10 (Py-C2/C4), 112.24, 111.91 (BeIm-C4-C7), 53.18 (Py-***CH₂***), 43.75 (Et-***CH₂***) 15.84 (Et-***CH₃***)

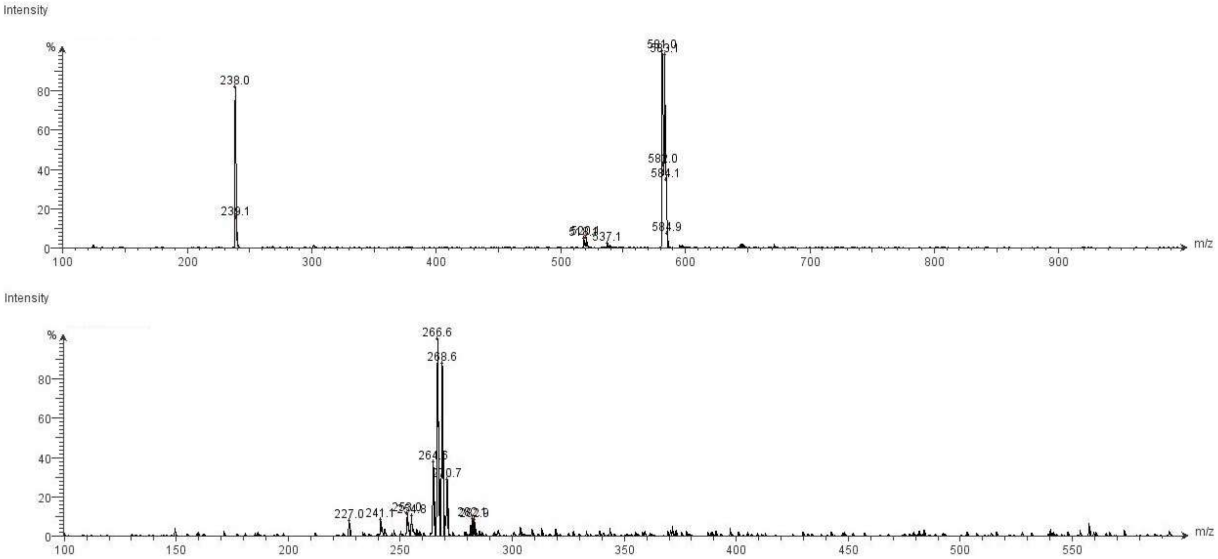

Figure S32. **2b**, positive (top) and negative (bottom) ESI-MS: m/z (ESI+) 580.9 [NHC-Ag-NHC]$^+$, 238.0 [M-AgCl]$^+$; (ESI-): 266.7 [Br-Ag-Br]$^-$

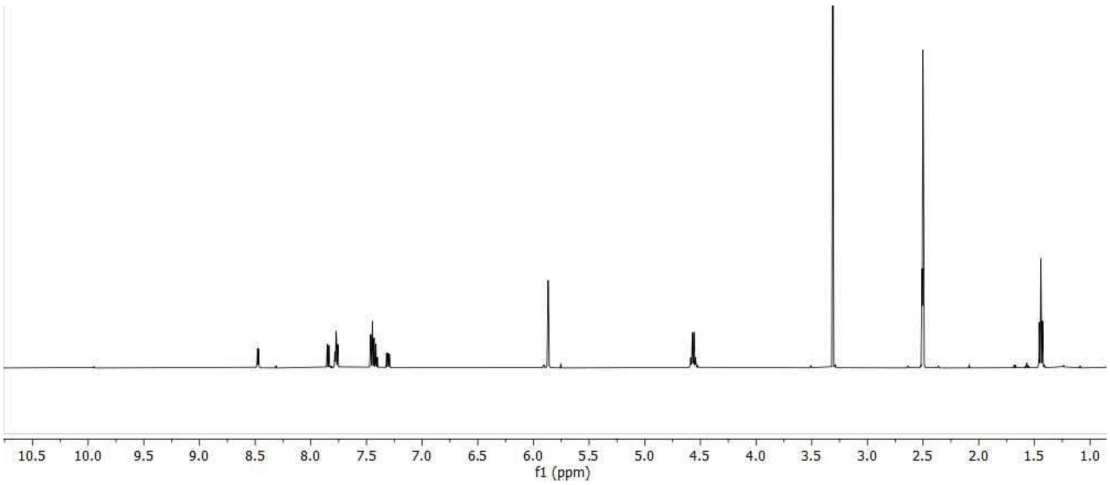

Figure S33. **2c**, ^{1}H-NMR (500 MHz, DMSO- d_6) δ = 8.47 (ddd, $J_{H,H}$ = 4.8, 1.8, 0.9 Hz, Py-H5, 1H), 7.86-7.83 (BeIm-H4/H7, 1H), 7.79-7.75 (m, Py -H2-H3, 1H), 7.45 (dt, $J_{H,H}$ = 7.6, 1.1 Hz, BeIm-H4/H7, 2H), 7.44-7.40 (m, eIm-H4/H7, 1H), 7.31 (ddd, $J_{H,H}$ = 7.6, 4.9, 1.2 Hz, Py-H4, 1H), 5.87 (s, Py-***CH_2***, 2H), 4.57 (q, $J_{H,H}$ = 7.2 Hz Et-***CH_2***, 2H), 1.44 (t, $J_{H,H}$ = 7.2 Hz, Et-***CH_3***, 3H)

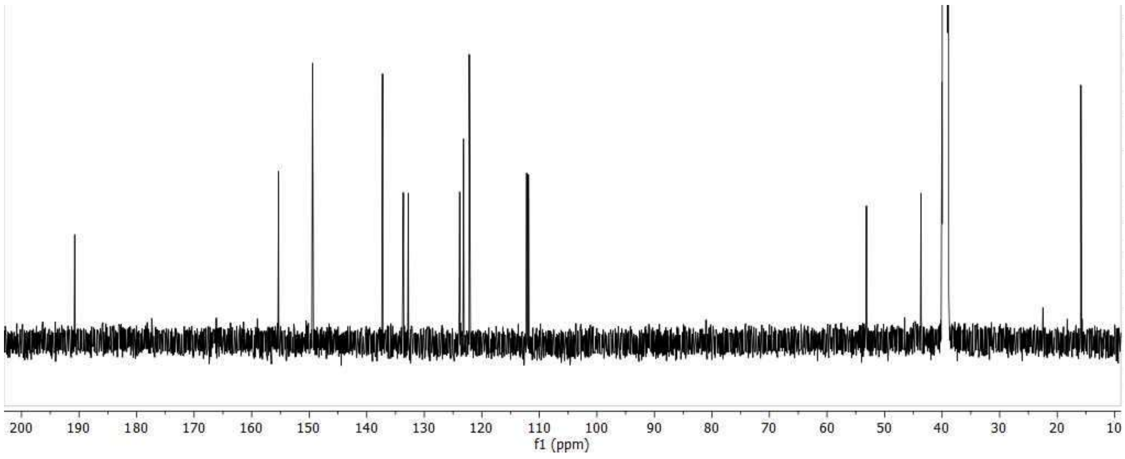

Figure S34. 2c, 13C-NMR (126 MHz, DMSO-d_6) δ = 190.76 (BeIm-C2), 155.32 (Py-C1), 149.42 (Py-C5), 137.25 (Py-C3), 133.64, 132.78, 123.91, 123.84 (BeIm-C4-C7), 123.17, 122.17 (Py-C2/C4), 112.20, 111.88 (BeIm-C4-C7), 53.14 (Py- ***CH_2***), 43.66 (Et- ***CH_2***) 15.87 (Et- ***CH_3***)

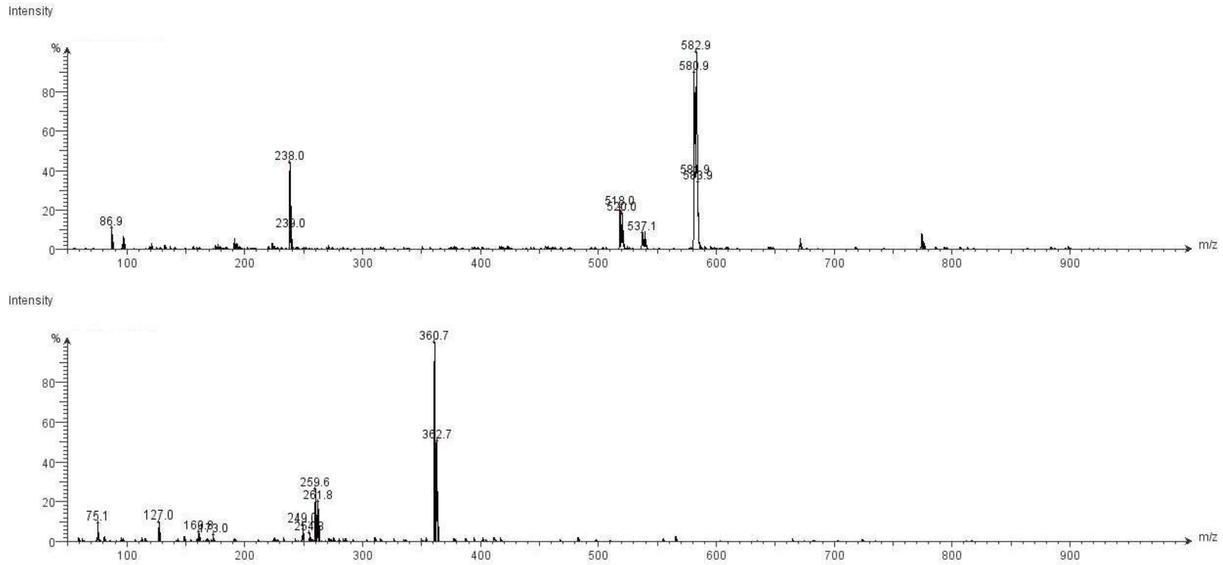

Figure 35. 2c, positive (top) and negative (bottom) ESI-MS: m/z (ESI+) 580.9 [NHC-Ag-NHC]$^+$, 238.0 [M-AgCl]$^+$; (ESI-): 360.7 [I-Ag-I]$^-$

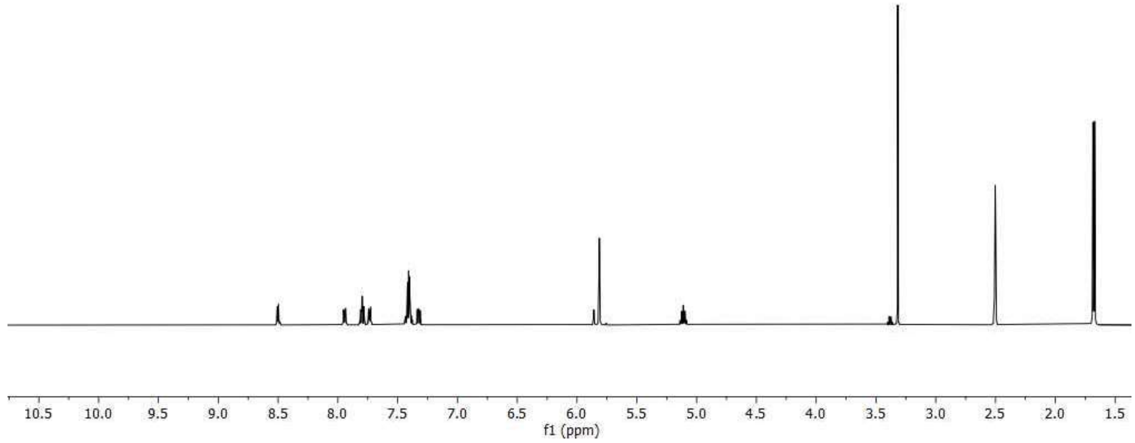

Figure S36. **3a** ^{1}H-NMR (500 MHz, DMSO-d_6) δ = 8.50 (ddd, $J_{H,H}$ = 5.3, 1.8, 0.9 Hz, Py-H5, 1H), 7.98 –7.90 (m, Py -H2-H3, 1H), 7.80 (tdd, $J_{H,H}$ = 7.7, 4.0, 1,8 Hz BeIm-H4/H7, 1H), 7.77 – 7.69 (m, BeIm-H4/H7, 1H), 7.46 – 7.35 (m, BeIm-H4/H7, 3H) 7.32 (ddd, $J_{H,H}$ = 7.6, 4,8, 1.1 Hz Py-H4, 1H), 5.81 (s, Py-***CH₂***, 2H), 5.11 (hept, $J_{H,H}$ = 6.9 Hz, iPr-***CH***, 1H) 1.68 (d, $J_{H,H}$ = 6.9 Hz, iPr-***CH₃***, 6H)

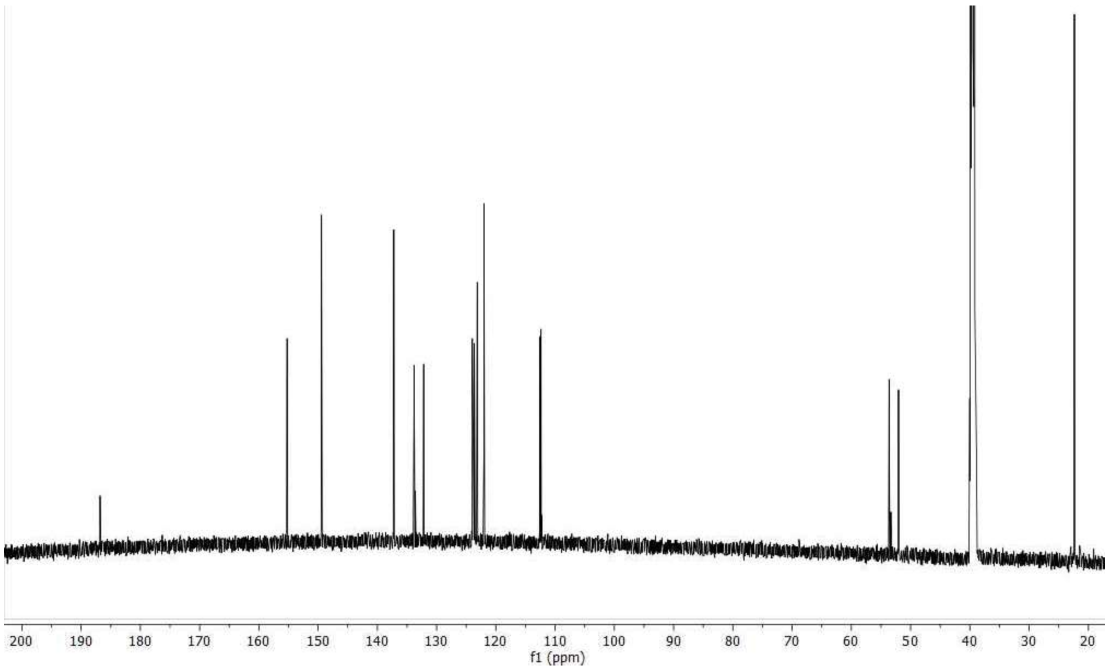

Figure S37. 3a, ^{13}C-NMR (126 MHz, DMSO-d_6) δ = 186.79 (BeIm-C2), 155.24 (Py-C1), 149.44 (Py-C5), 137.23 (Py-C3), 133.82, 132.13, 123.98, 123.64 (BeIm-C4-C7), 123.13, 122.01 (Py-C2/C4), 112.57, 112.38 (BeIm-C4-C7), 53.62 (Py-***CH_2***), 52.04 (iPr-***CH***) 22.35 (iPr-***CH_3***)

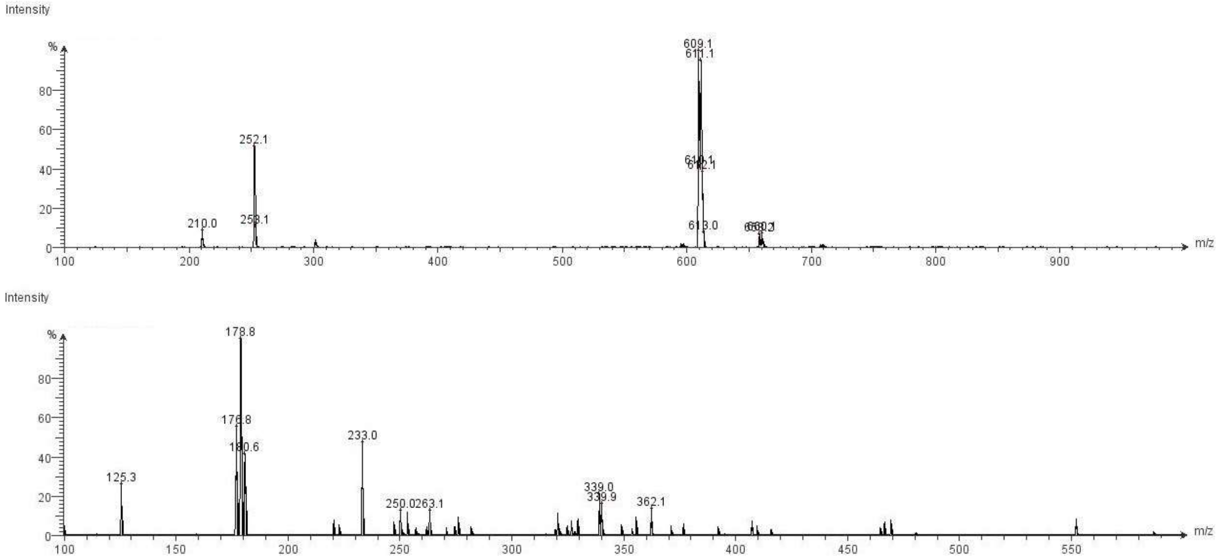

Figure S38. **3a**, positive (top) and negative (bottom) ESI-MS: m/z (ESI+) 609.0 [NHC-Ag-NHC]$^+$, 252.0[M-AgCl]$^+$; (ESI-): 178.7 [Cl-Ag-Cl]$^-$

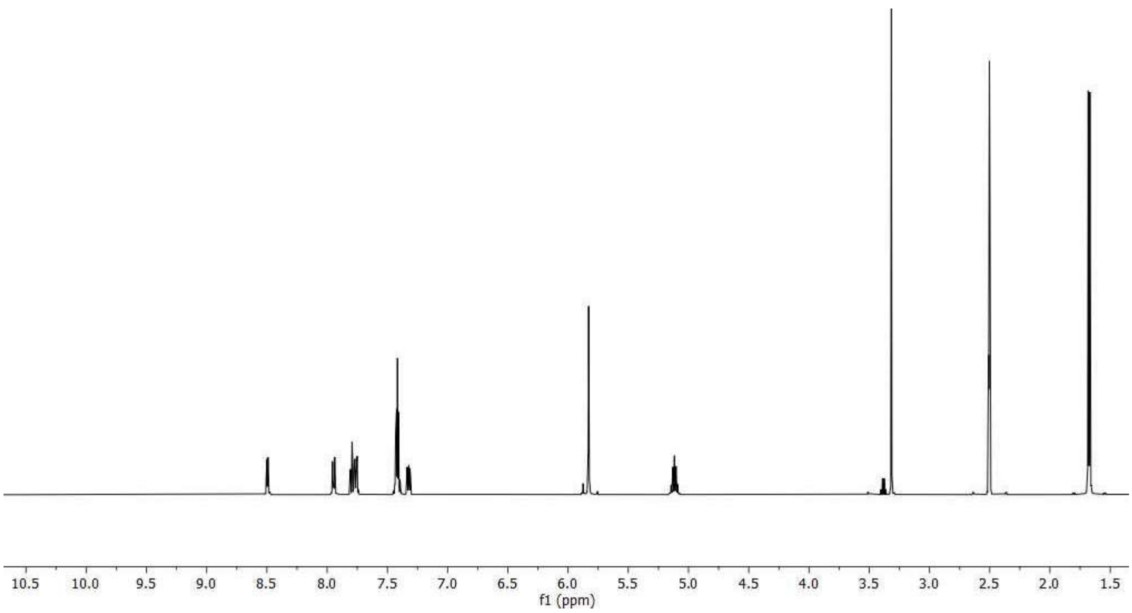

Figure S39. 3b, ^{1}H-NMR (500 MHz, DMSO-*d_6*) δ = 8.50 (ddd, $J_{H,H}$ = 4.9, 1.8, 0.9 Hz, Py-H5, 1H), 7.99 – 7.90 (m, Py -H2-H3, 1H), 7.83-7.72 (m, BeIm-H4/H7, 2H), 7.47 – 7.37 (m, BeIm-H4/H7, 3H), 7.32 (ddd, $J_{H,H}$ = 7.5, 4,8, 1.1 Hz Py-H4, 1H), 5.83 (s, Py-***CH_2***, 2H), 5.12 (hept, $J_{H,H}$ = 6.9 Hz, iPr-***CH***, 1H) 1.68 (d, $J_{H,H}$ = 6.9 Hz, iPr-***CH_3***, 6H)

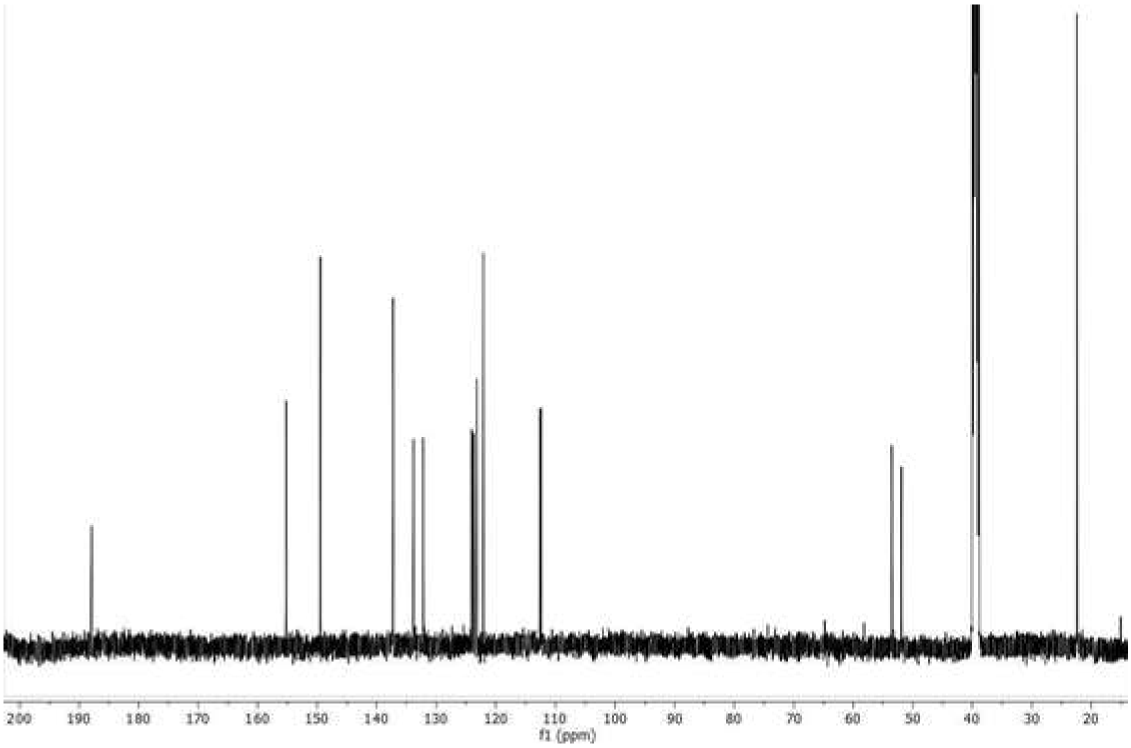

Figure S40. 3b, ^{13}C-NMR (126 MHz, DMSO-*d_6*) δ = 187.88 (BeIm-C2), 155.20 (Py-C1), 149.44 (Py-C5), 137.26 (Py-C3), 133.79, 132.20 (BeIm-C4-C7), 124.00, 123.67, 123.16,122.06 (Py-C2/C4), 112.57, 112.34 (BeIm-C4-C7), 53.53 (Py-***CH_2***), 51.89 (iPr-***CH***) 22.40 (iPr-***CH_3***)

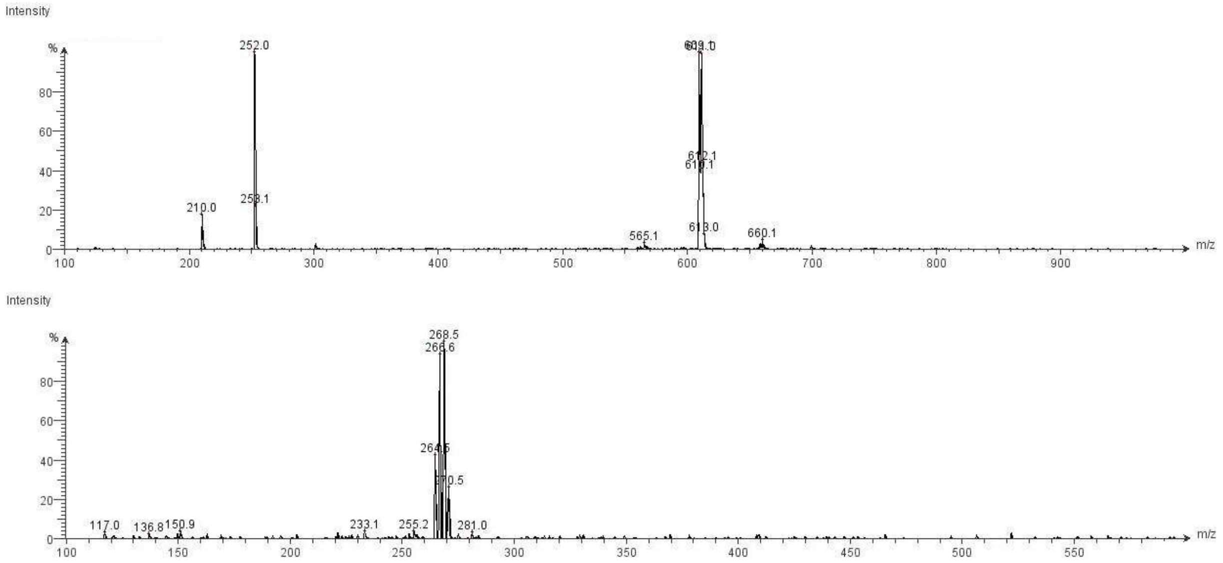

Figure S41. **3b**, positive (top) and negative (bottom) ESI-MS: m/z (ESI+) 609.0 [NHC-Ag-NHC]$^+$, 252.0[M-AgCl]$^+$; (ESI-): 266.7 [Br-Ag-Br]$^-$

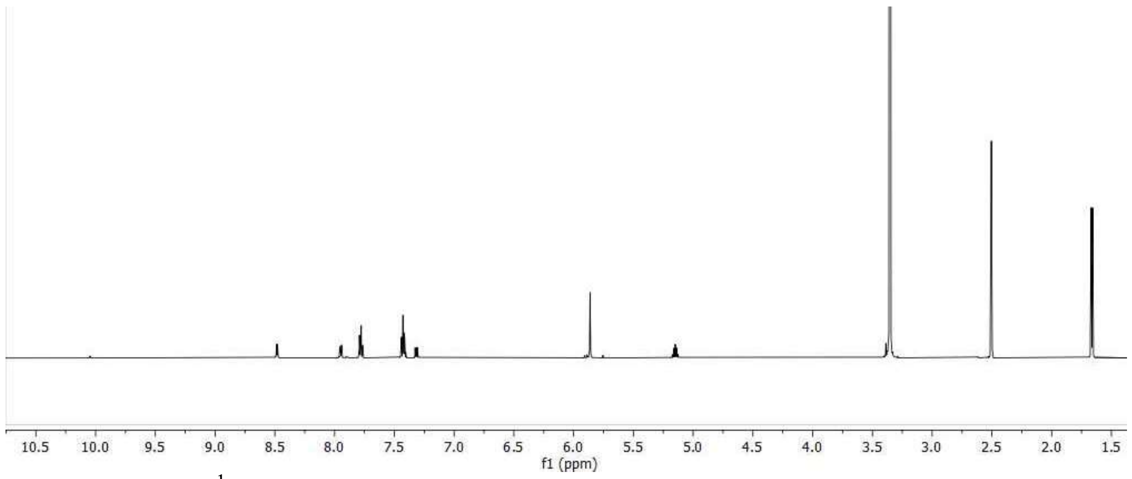

Figure S42. 3c, ^{1}H-NMR (600 MHz, DMSO-d_6) δ = 8.48 (ddd, $J_{H,H}$ = 4.9, 1.8, 0.9 Hz, Py-H5, 1H), 7.98 –7.91 (m, Py -H2-H3, 1H), 7.82 – 7.75 (m, BeIm-H4/H7, 2H), 7.45 – 7.41 (m, BeIm-H4/H7, 3H) 7.32 (ddd, $J_{H,H}$ = 7.6, 4.8, 1.1 Hz Py-H4, 1H), 5.86 (s, Py-***CH_2***, 2H), 5.16 (hept, $J_{H,H}$ = 6.8 Hz, iPr-***CH***, 1H) 1.66 (d, $J_{H,H}$ = 6.9 Hz, iPr-***CH_3***, 6H)

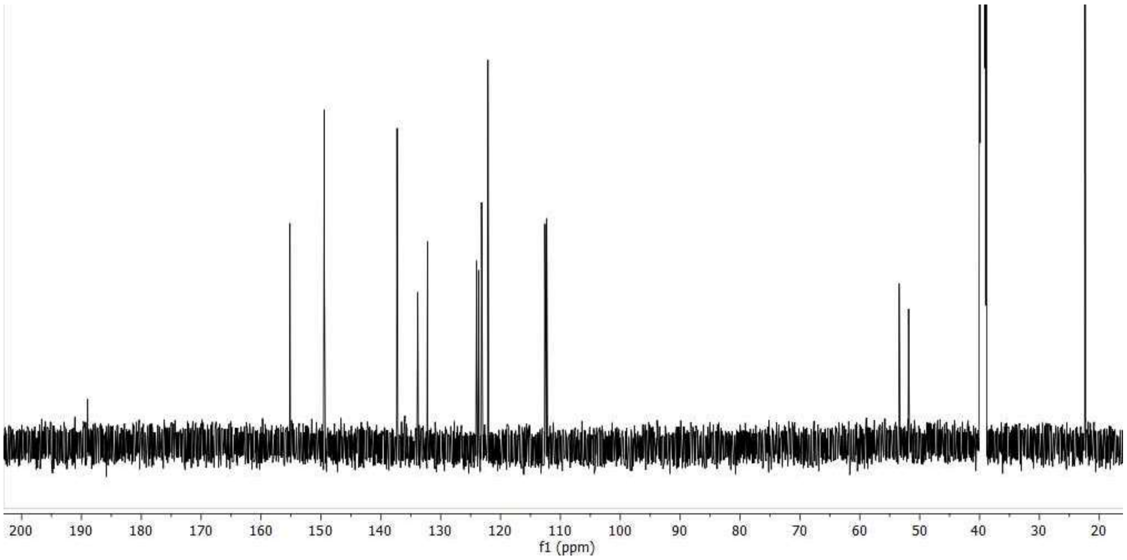

Figure S43. 3c, ^{13}C-NMR (151 MHz, DMSO-d_6) δ = 189.13 (BeIm-C2), 155.27 (Py-C1), 149.54 (Py-C5), 137.39 (Py-C3), 133.94, 132.30, 124.11, 123.79 (BeIm-C4-C7), 123.29, 122.22 (Py-C2/C4), 112.71, 112.39 (BeIm-C4-C7), 53.52 (Py-***CH₂***), 51.96 (iPr-***CH***) 22.49 (iPr-***CH₃***)

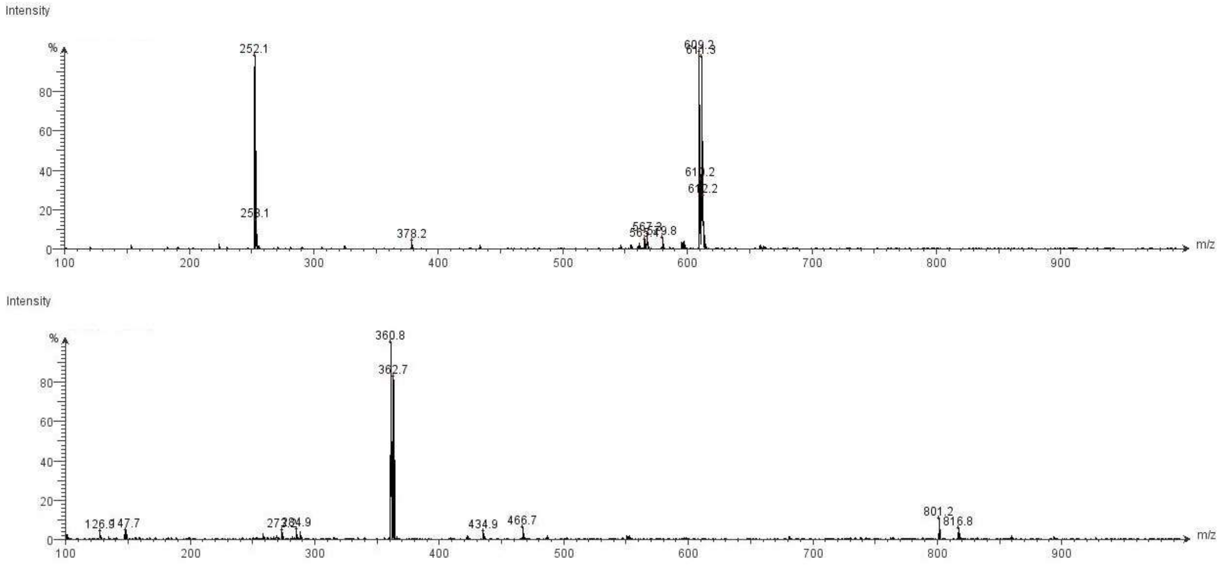

Figure S44. **3c**, positive (top) and negative (bottom) ESI-MS: m/z (ESI+) 609.0 [NHC-Ag-NHC]$^+$, 252.0[M-AgCl]$^+$; (ESI-): 360.7 [I-Ag-I]$^-$

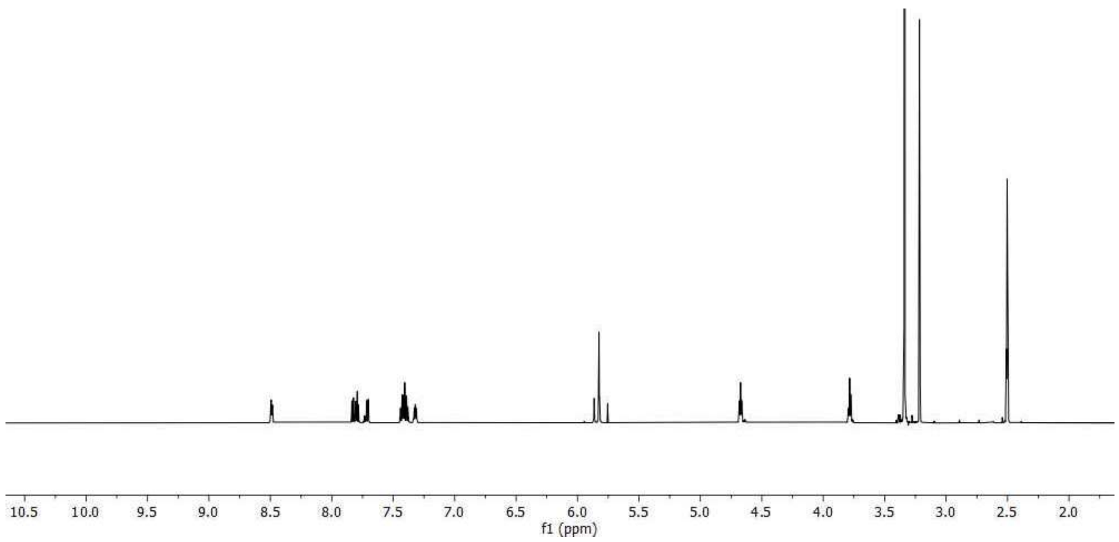

Figure S45. 4a, ^{1}H-NMR (600 MHz, DMSO-d_6) δ = 8.46 (ddd, $J_{H,H}$ = 4.8, 1.9, 0.9 Hz, Py-H5, 1H), 7.86– 7.75 (m, Py -H2-H3, 2H), 7.75-7.68 (m, BeIm-H4/H7, 1H), 7.47-7.35 (m, BeIm-H4/H7, 3H), 7.32 (ddd, $J_{H,H}$ = 7.6, 4.9, 1.2 Hz , Py-H4, 1H), 5.87 (s, Py-$\boldsymbol{CH_2}$, 2H), 4.70-4.61 (m, N-$CH_2$$\boldsymbol{CH_2}$$OCH_3$, 2H), 3.81-3.74 (m, N-$\boldsymbol{CH_2}$$CH_2OCH_3$, 2H), 3.22 (s, N-$CH_2CH_2O$$\boldsymbol{CH_3}$, 3H)

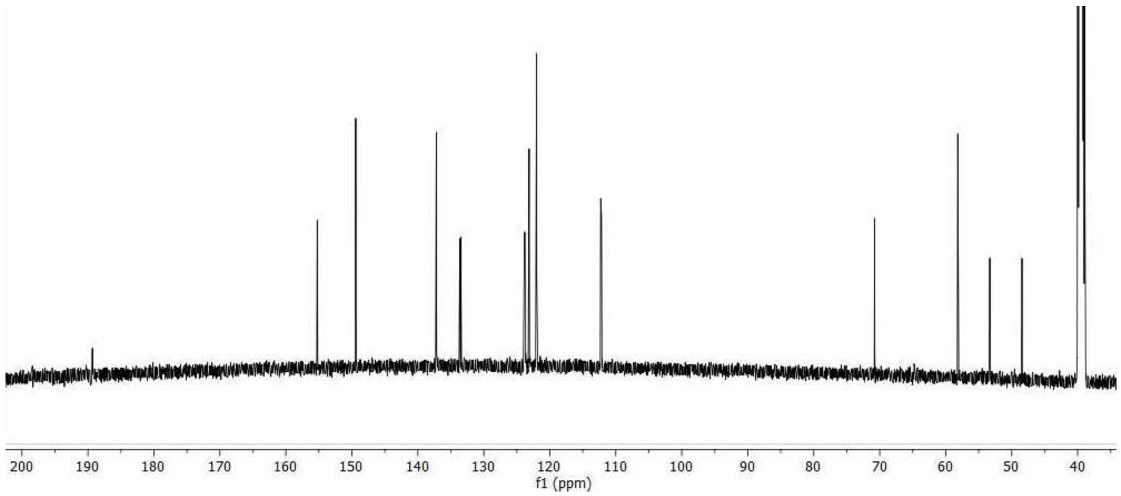

Figure S46. 4a, ^{13}C-NMR (151 MHz, DMSO-d_6) δ = 189.33 (BeIm-C2), 155.24 (Py-C1), 149.40 (Py-C5), 137.19 (Py-C3), 133.62, 133.44, 123.83, 123.74 (BeIm-C4-C7), 123.11, 121.98 (Py-C2/C4), 112.28, 112.15 (BeIm-C4-C7), 70.76 (N-$CH_2$$\boldsymbol{CH_2}$OCH), 58.16 (N-$\boldsymbol{CH_2}$$CH_2OCH_3$), 53.31 (Py-$\boldsymbol{CH_2}$), 48.43 (N-$CH_2CH_2O$$\boldsymbol{CH_3}$)

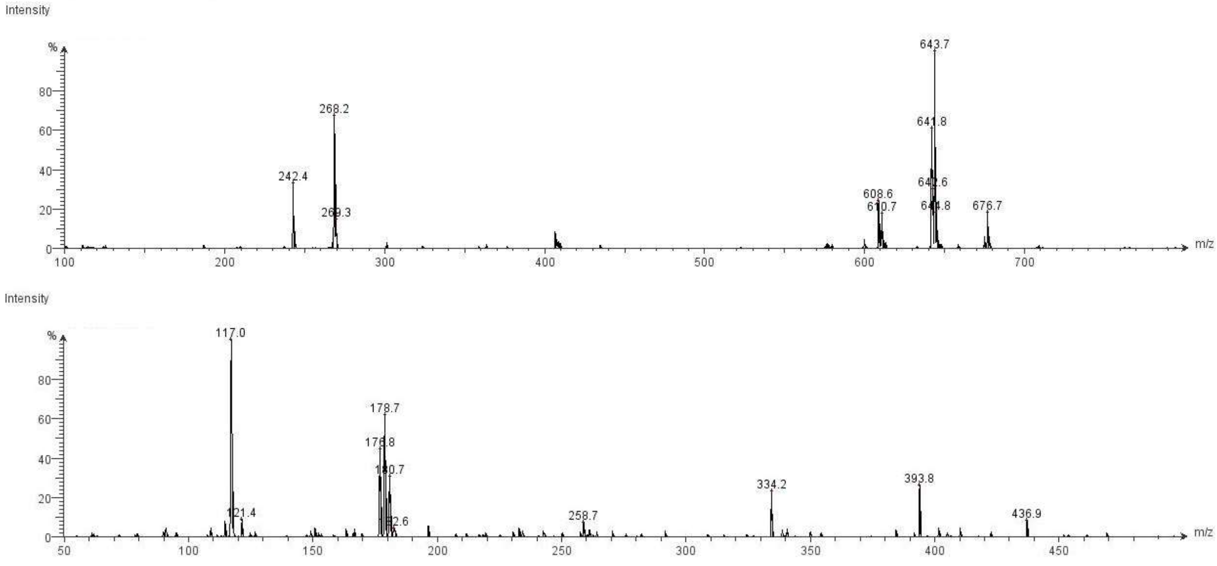

Figure S47. **4a**, positive (top) and negative (bottom) ESI-MS: m/z (ESI+) 641.2 [NHC-Ag-NHC]$^+$, 268.1 [M-AgCl]$^+$; (ESI-): 178.7 [Cl-Ag-Cl]$^+$

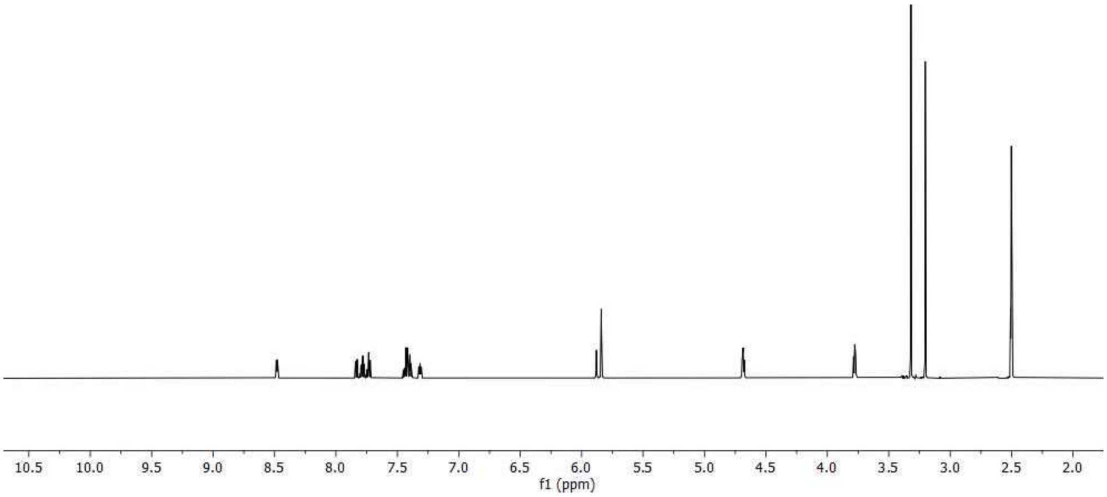

Figure S48. 4b, ^{1}H-NMR (600 MHz, DMSO-d_6) δ = 8.48 (ddd, $J_{H,H}$ = 4.9, 1.8, 0.9 Hz, Py-H5, 1H), 7.86 – 7.68 (m, Py -H2-H3/BeIm-H4/H7, 3H), 7.47-7.36 (m, BeIm-H4/H7, 3H), 7.31 (ddd, $J_{H,H}$ = 7.6, 4.9, 1.2 Hz , Py-H4, 1H), 5.84 (s, Py-$\boldsymbol{CH_2}$, 2H), 4.68 (t, $J_{H,H}$ = 5.2 Hz N-$CH_2\boldsymbol{CH_2}OCH_3$, 2H), 3.79-3.76 (m, N-$\boldsymbol{CH_2}CH_2OCH_3$, 2H), 3.21 (s, N-$CH_2CH_2O\boldsymbol{CH_3}$, 3H)

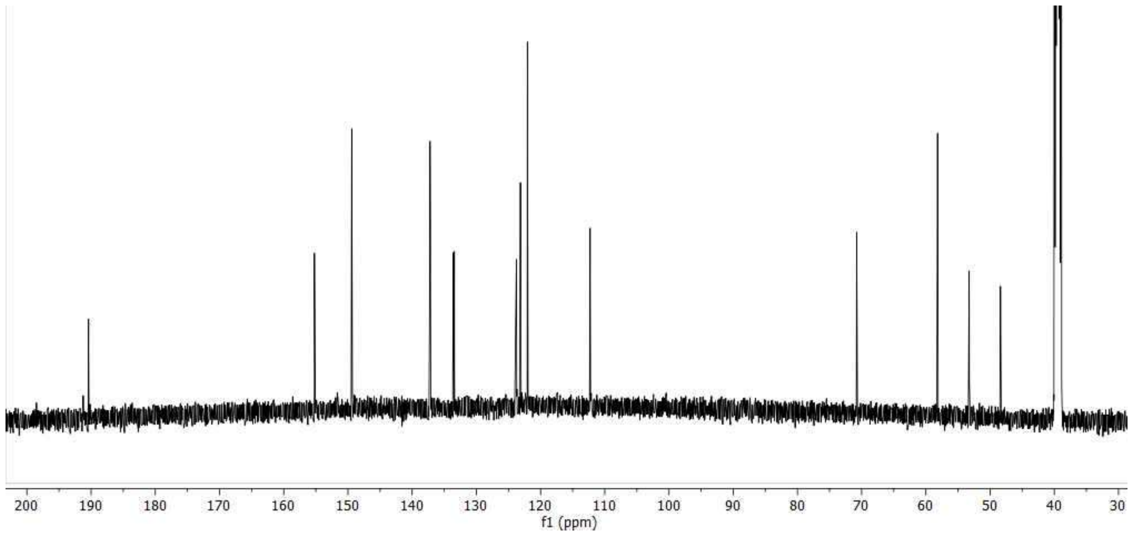

Figure S49. 4b, ^{13}C-NMR (151 MHz, DMSO-d_6) δ = 190.38 (BeIm-C2), 155.22 (Py-C1), 149.40 (Py-C5), 137.21 (Py-C3), 133.62, 133.42, 123.85, 123.76 (BeIm-C4-C7), 123.14, 122.04 (Py-C2/C4), 112.28, 112.15 (BeIm-C4-C7), 70.77 (N-CH$_2$***CH$_2$***OCH$_3$), 58.17 (N-***CH$_2$***CH$_2$OCH$_3$), 53.25 (Py-***CH$_2$***), 48.38 (N-CH$_2$CH$_2$O***CH$_3$***)

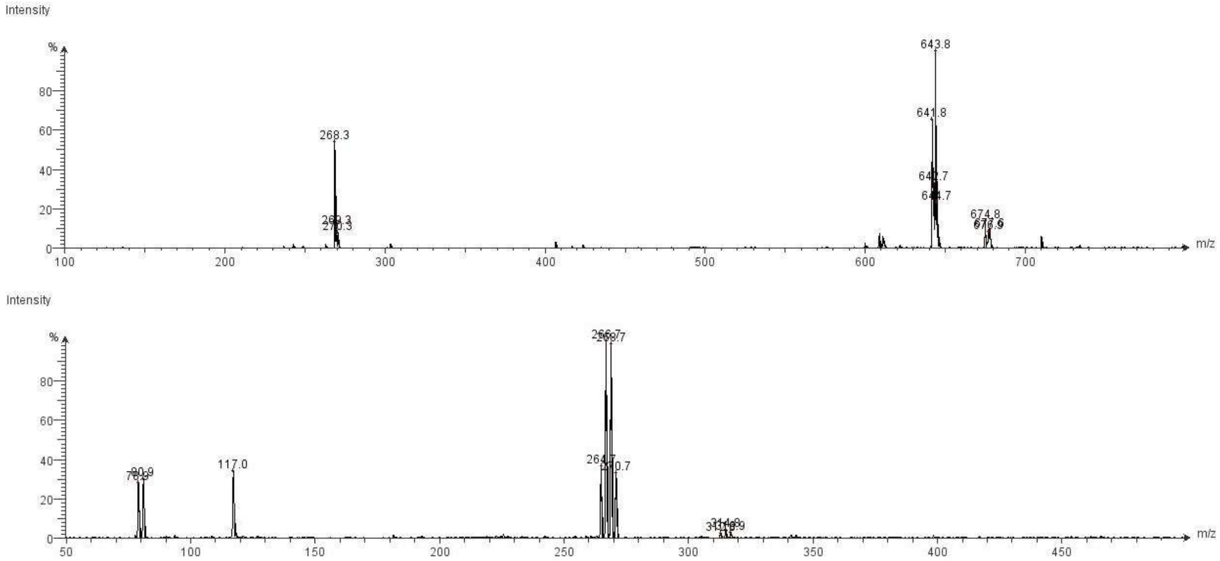

Figure S50. **4b**, positive (top) and negative (bottom) ESI-MS: m/z (ESI+) 641.2 [NHC-Ag-NHC]$^+$, 268.1 [M-AgCl]$^+$; (ESI-): 266.7 [Br-Ag-Br]$^-$

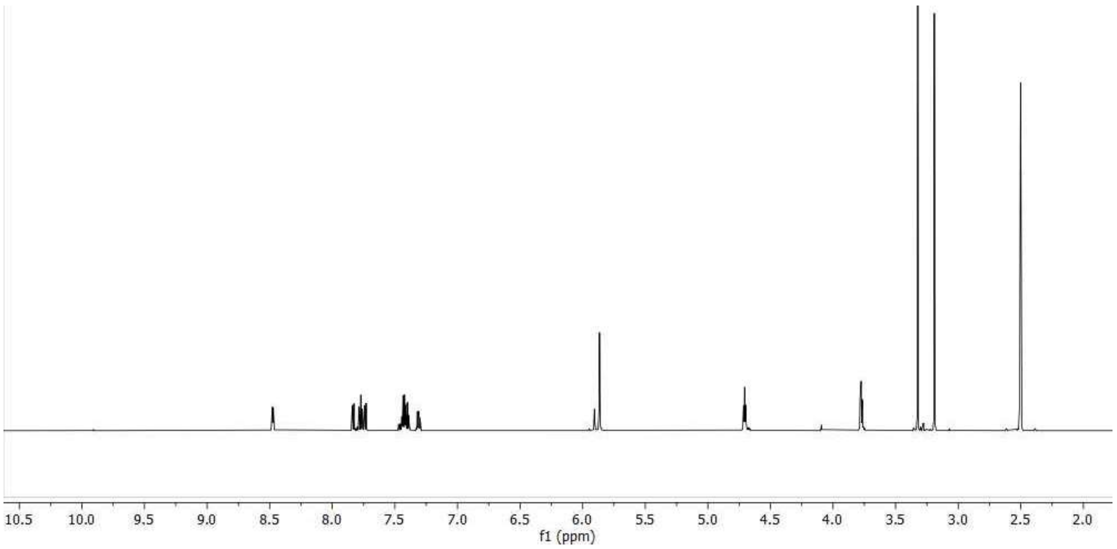

Figure S51. 4c, ^{1}H-NMR (600 MHz, DMSO-d_6) δ = 8.46 (ddd, $J_{H,H}$ = 4.7, 1.8, 0.9 Hz, Py-H5, 1H), 7.87 – 7.74 (m, Py -H2-H3, 2H), 7.78-7.71 (m, BeIm-H4/H7, 1H), 7.48-7.36 (m, BeIm-H4/H7, 3H), 7.30 (ddd, $J_{H,H}$ = 7.6, 4.9, 1.2 Hz , Py-H4, 1H), 5.87 (s, Py-***CH_2***, 2H), 4.71 (t, $J_{H,H}$ = 5.2 Hz, N-CH_2***CH_2***OCH_3, 2H), 3.77 (dd, $J_{H,H}$ = 5.6, 4.7 Hz, N-***CH_2***CH_2OCH_3, 2H), 3.22 (s, N-CH_2CH_2O***CH_3***, 3H)

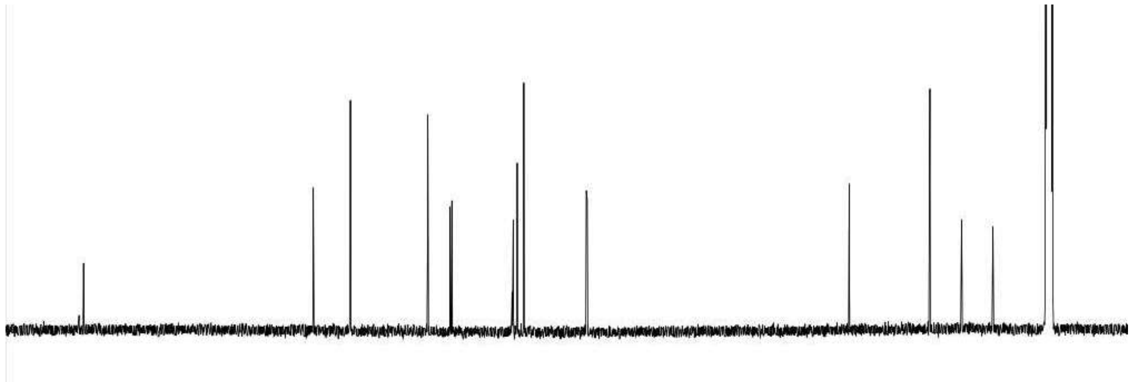

Figure S52. 4c, ^{13}C-NMR (151 MHz, DMSO-d_6) δ = 191.32 (BeIm-C2), 155.22 (Py-C1), 149.38 (Py-C5), 137.21 (Py-C3), 133.68, 133.40, 123.84, 123.76 (BeIm-C4-C7), 123.14, 122.09 (Py-C2/C4), 112.26, 112.12 (BeIm-C4-C7), 70.88 (N-CH_2***CH_2***OCH_3), 58.19 (N-***CH_2***CH_2OCH_3), 53.19 (Py-***CH_2***), 48.30 (N-CH_2CH_2O***CH_3***)

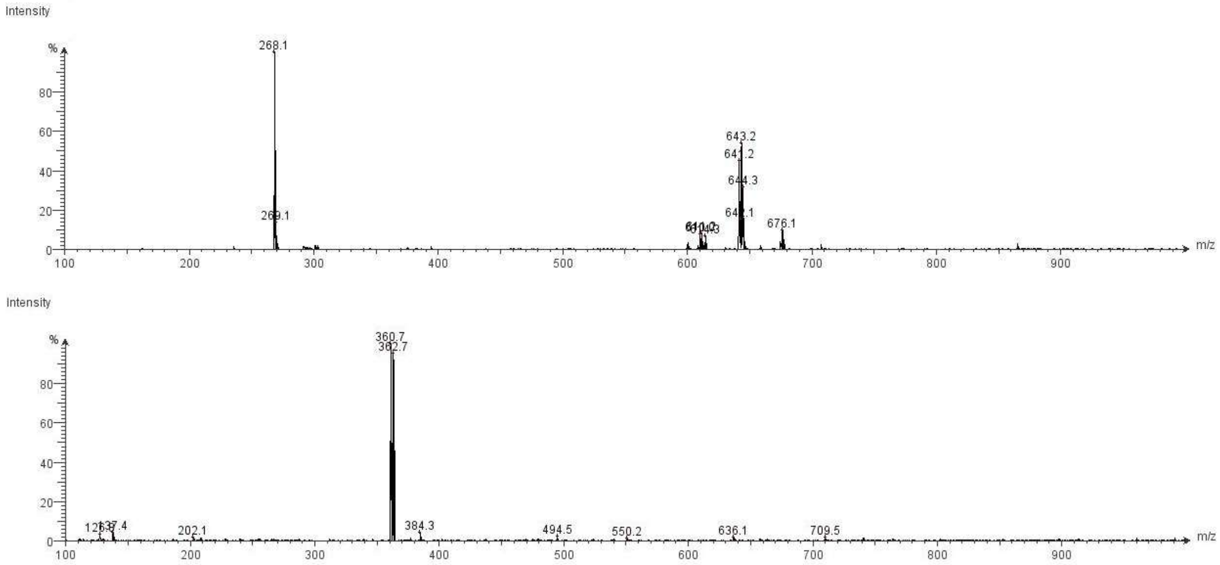

Figure S53. **4c**, positive (top) and negative (bottom) ESI-MS: m/z (ESI+) 641.2 [NHC-Ag-NHC]$^+$, 268.1 [M-AgCl]$^+$; (ESI-): 360.7 [I-Ag-I]$^-$

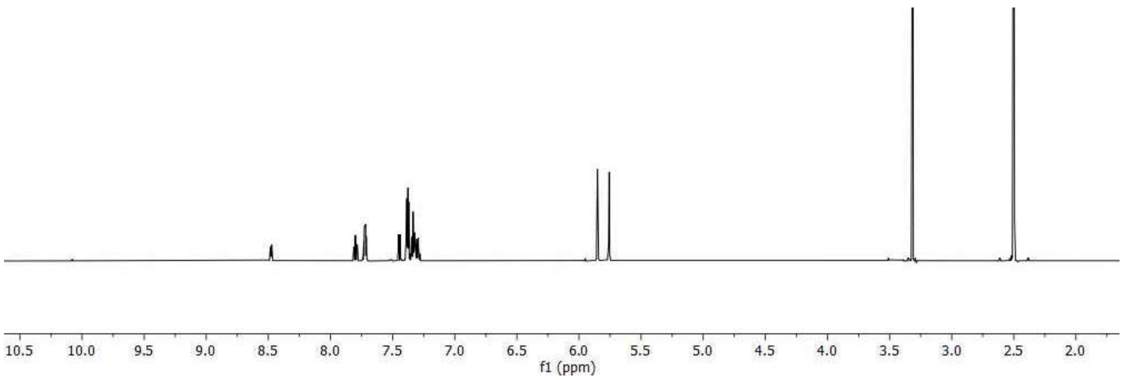

Figure S54. 5a, ^{1}H-NMR (600 MHz, DMSO-*d$_6$*) δ = 8.48 (ddd, $J_{H,H}$ = 4.8, 1.8, 0.9 Hz, Py-H5, 1H), 7.80 (td, $J_{H,H}$ = 7.7, 1.8 Hz, BeIm-H4/H7, 1H), 7.75-7.69 (m, BeIm-H4/H7, 2H), 7.44 (dt, $J_{H,H}$ = 7.8, 1.1 Hz, BeIm-H4/H7, 1H), 7.41-7.36 (m, Bn-H2-H6, 4H), 7.38– 7.26 (m, Py-H2-H4, 3H), 5.85 (s, Py-***CH$_2$***, 2H), 5.76 (s, Bn-***CH$_2$***, 2H)

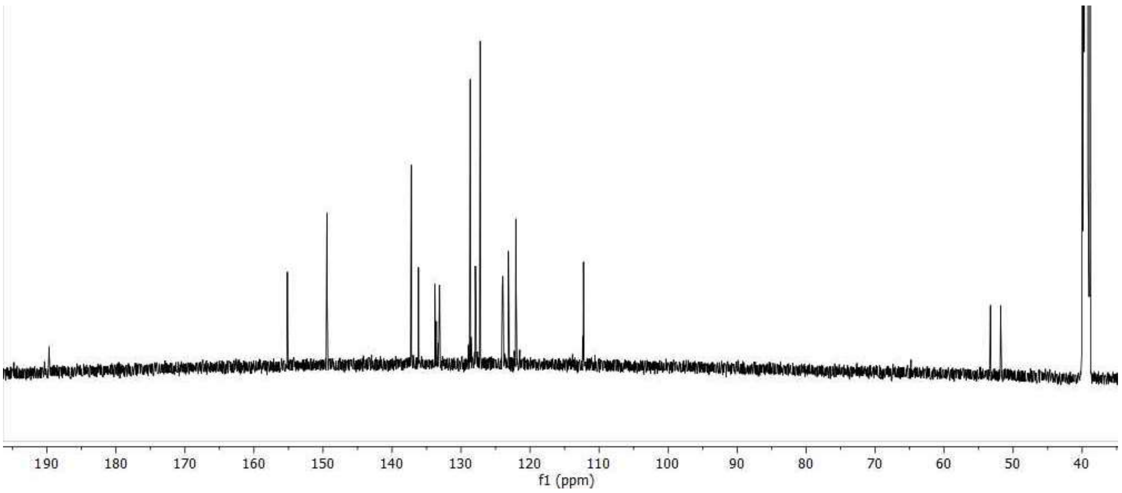

Figure S55. 5a, ^{13}C-NMR (151 MHz, DMSO-d_6) δ = 189.69 (BeIm-C2), 155.16 (Py-C1), 149.43 (Py-C5), 137.21 (Py-C3), 136.16 (Bn-C1), 133.78, 133.12 (BeIm-C4-C7), 128.68, 127.92, 127.23 (Bn-C2-C6) 124.01, 123.94 (BeIm-C4-C7), 123.13, 122.06 (Py-C2/C4), 112.35, 112.26 (BeIm-C4-C7), 53.30 (Py-***CH₂***), 51.81 (Bn-***CH₂***)

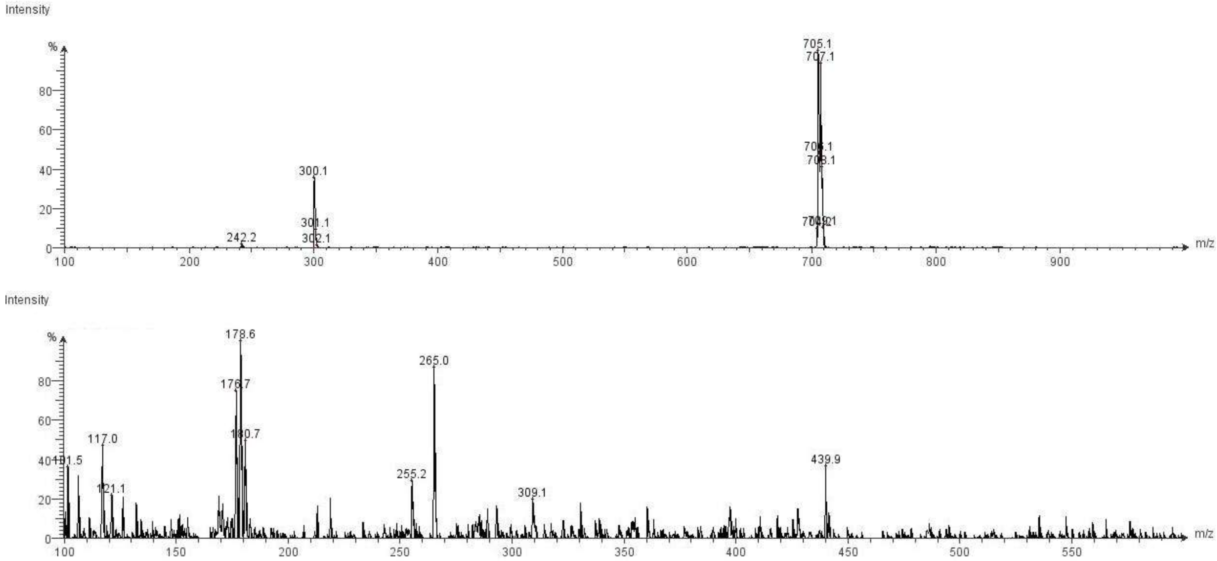

Figure S56. **5a**, positive (top) and negative (bottom) ESI-MS: m/z (ESI+) 705.1 [NHC-Ag-NHC]$^+$, 300.1[M-AgCl]$^+$; (ESI-): 178.7 [Cl-Ag-Cl]$^-$

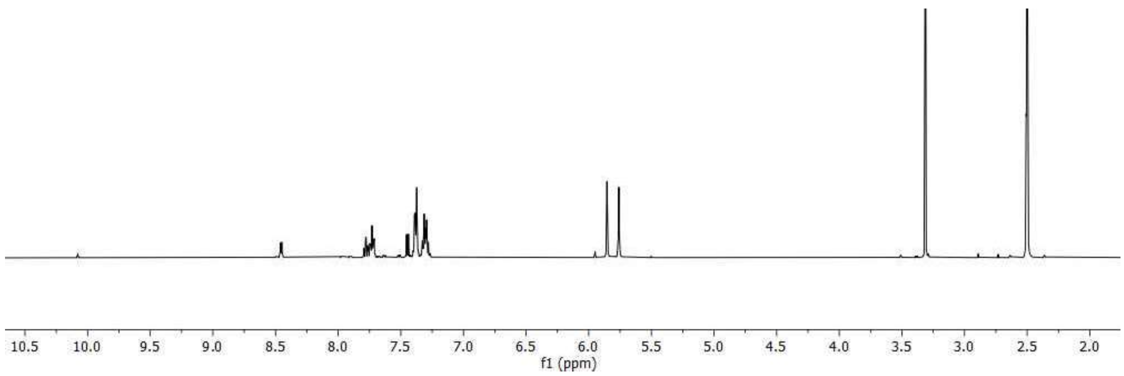

Figure S57. 5b, ^{1}H-NMR (500 MHz, DMSO-d_6) δ = 8.45 (ddd, $J_{H,H}$ = 5.0, 1.8, 0.9 Hz, Py-H5, 1H), 7.81-7.60 (m, BeIm-H4-H7, 3H), 7.45 (dd, $J_{H,H}$ = 7.9, 1.3 Hz, BeIm-H4/H7, 1H), 7.38 (dt, $J_{H,H}$ = 7.3, 1.9 Hz, Bn-H2-H6, 4H), 7.35– 7.26 (m, Py-H2-H4, 3H), 5.85 (s, Py-***CH₂***, 2H), 5.76 (s, Bn-***CH₂***, 2H)

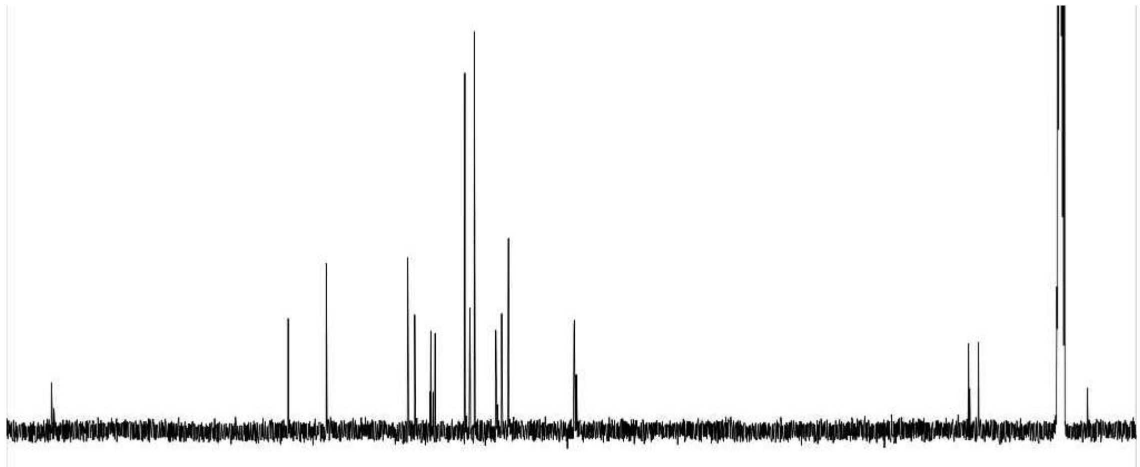

Figure S58. 5b, ^{13}C-NMR (126 MHz, DMSO-d_6), δ = 190.52 (BeIm-C2), 155.12 (Py-C1), 149.42 (Py-C5), 137.21 (Py-C3), 136.16 (Bn-C1), 133.76, 133.11 (BeIm-C4-C7), 128.67, 127.91, 127.25 (Bn-C2-C6) 124.02, 123.95 (BeIm-C4-C7), 123.15, 122.11 (Py-C2/C4), 112.33, 112.27 (BeIm-C4-C7), 53.24 (Py-***CH₂***), 51.77 (Bn-***CH₂***)

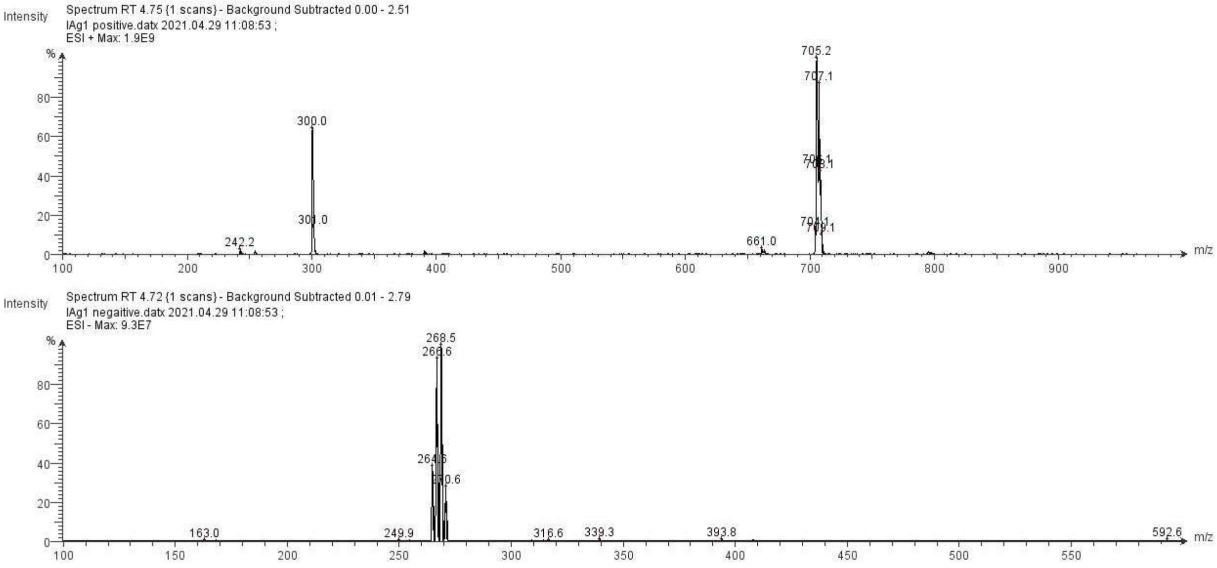

Figure S59. **5b**, positive (top) and negative (bottom) ESI-MS: m/z (ESI+) 705.1 [NHC-Ag-NHC]$^{+}$, 300.1[M-AgCl]$^{+}$; (ESI-): 266.7 [Br-Ag-Br]$^{-}$

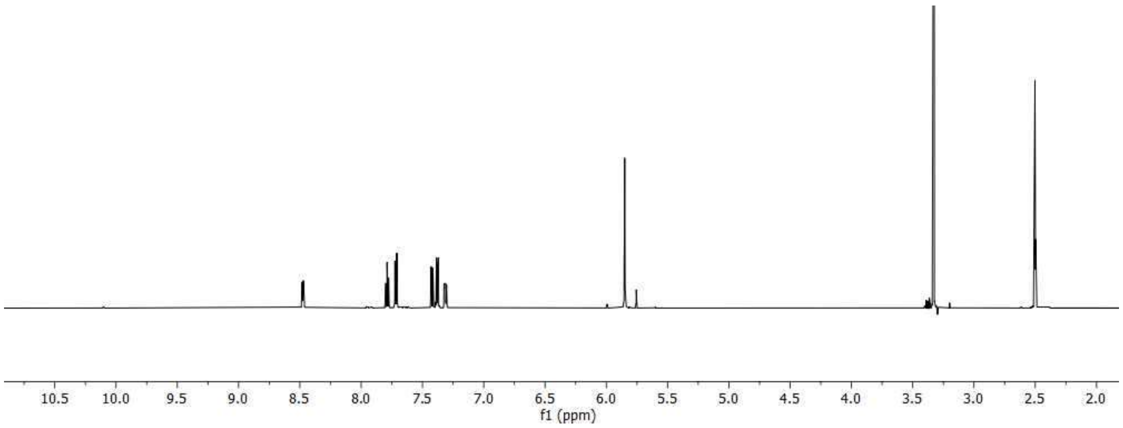

Figure S60. 6a, ^{1}H-NMR (600 MHz, DMSO-d_6) δ = 8.48 (ddd, $J_{H,H}$ = 4.8, 1.8, 0.9 Hz, Py-H5, 1H), , 7.79 (td, $J_{H,H}$ = 7.7, 1.8 Hz, BeIm-H4/H7, 1H), 7.75 – 7.62 (m, Py -H2-H3, 1H), 7.45-7.34 (m, BeIm-H4/H7, 2H), 7.36-7.28 (m, Py-H4, 1H), 5.85 (s, Py-***CH₂***, 4H)

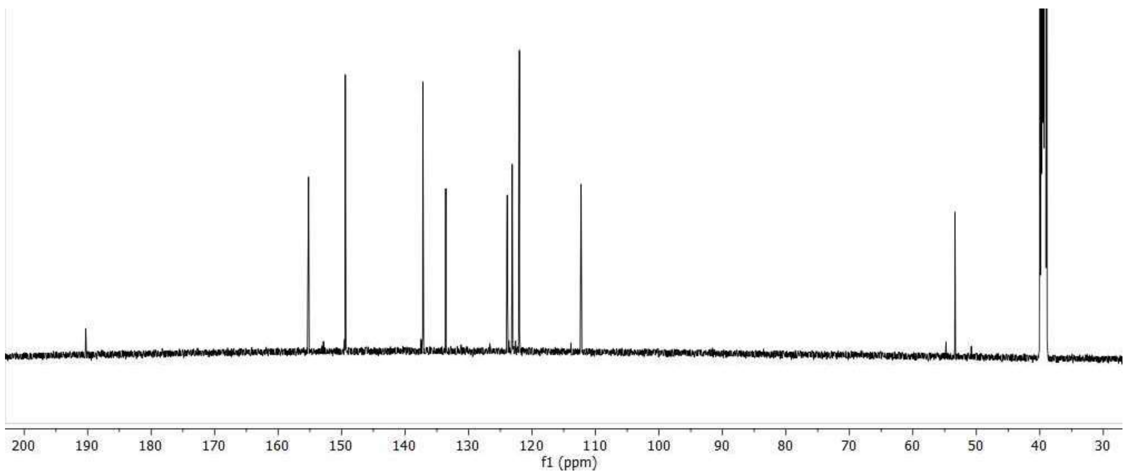

Figure S61. 6a, ^{13}C-NMR (151 MHz, DMSO-d_6) δ = 190.29 (BeIm-C2), 155.20 (Py-C1), 149.52 (Py-C5), 137.20 (Py-C3), 133.58, 123.90, 123.11 (BeIm-C4-C7), 121.99 (Py-C2/C4), 112.27 (BeIm-C4-C7), 53.36 (Py-***CH₂***)

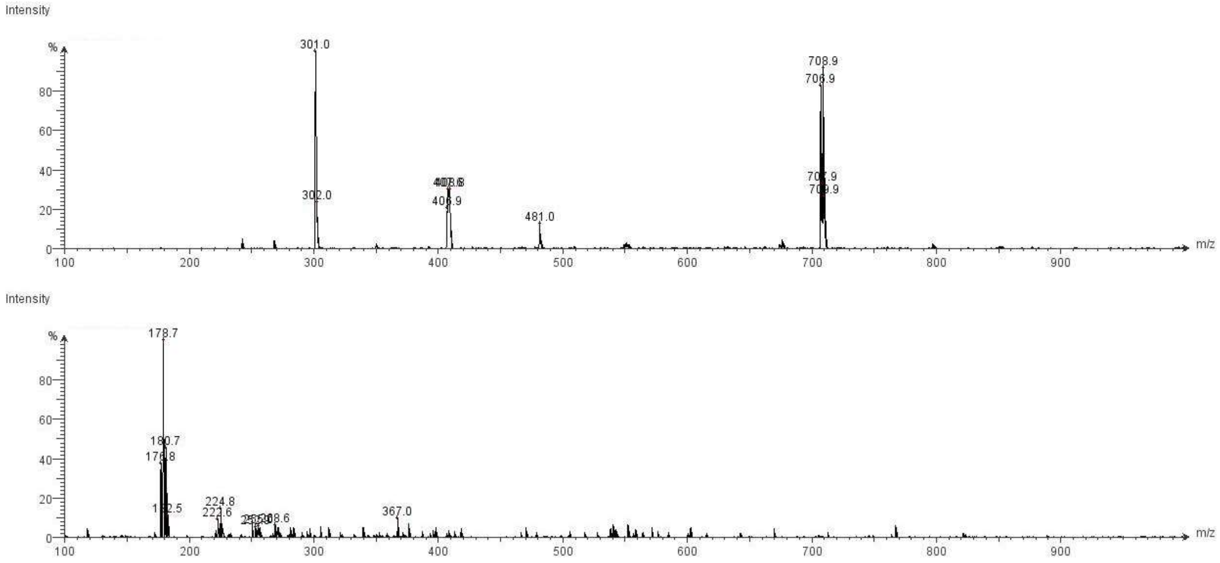

Figure S62. **6a**, positive (top) and negative (bottom) ESI-MS: m/z (ESI+) 707.9 [NHC-Ag-NHC]$^+$, 301.4[M-AgCl]$^+$; (ESI-): 178.7 [Cl-Ag-Cl]$^-$

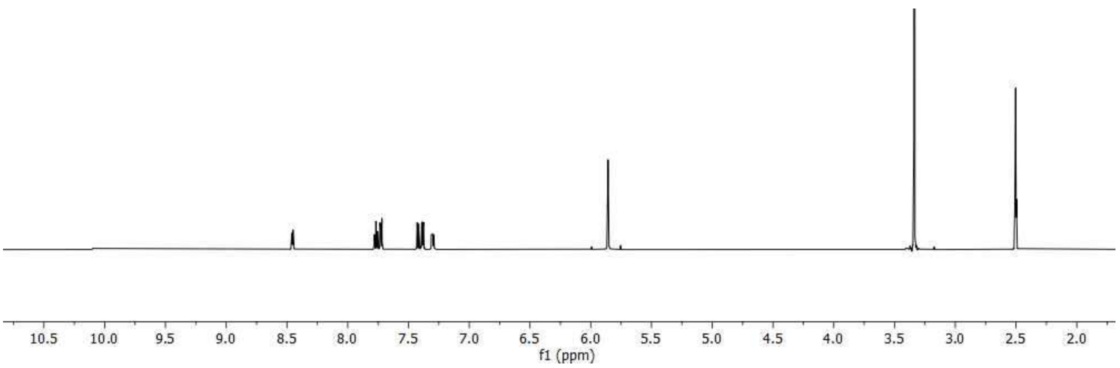

Figure S63. 6b, ^{1}H-NMR (600 MHz, DMSO-d_6) δ = 8.45 (ddd, $J_{H,H}$ = 4.8, 1.8, 0.9 Hz, Py-H5, 1H), , 7.77 (td, $J_{H,H}$ = 7.7, 1.8 Hz, BeIm-H4/H7, 1H), 7.76 – 7.70 (m, Py -H2-H3, 1H), 7.42 (dt, $J_{H,H}$ = 7.8, 1.1 Hz, BeIm-H4/H7, 1H), 7.38 (dd, $J_{H,H}$ = 6.2, 3.1 Hz, BeIm-H4/H7, 1H), 7.30 (ddd, $J_{H,H}$ = 7.6, 4.8, 1.1 Hz, Py-H4, 1H), 5.86 (s, Py-***CH₂***, 4H)

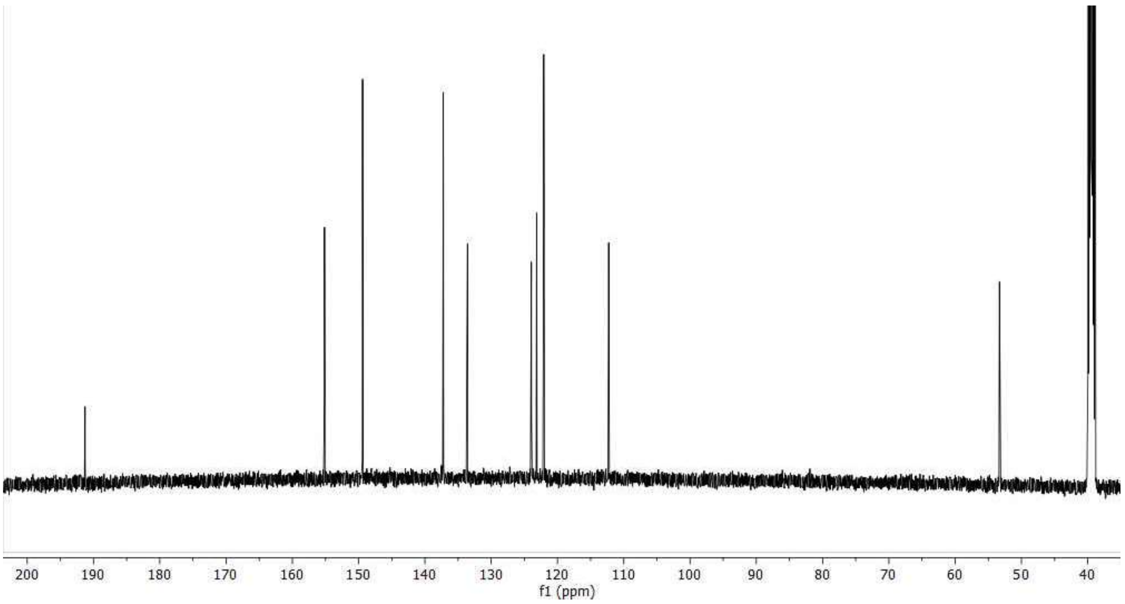

Figure S64. 6b, ^{13}C-NMR (151 MHz, DMSO-d_6) δ = 191.16 (BeIm-C2), 155.17 (Py-C1), 149.42 (Py-C5), 137.23 (Py-C3), 133.58, 123.95, 123.15 (BeIm-C4-C7), 122.06 (Py-C2/C4), 112.28 (BeIm-C4-C7), 53.33 (Py-$\boldsymbol{CH_2}$)

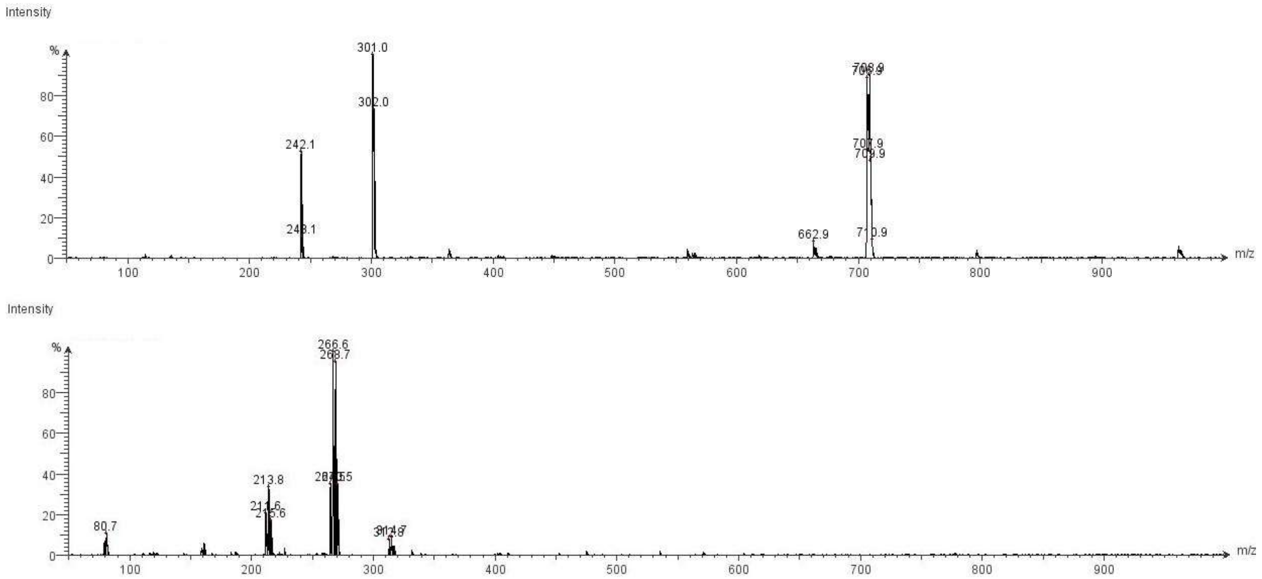

Figure S65. **6b**, positive (top) and negative (bottom) ESI-MS: m/z (ESI+) 707.9 [NHC-Ag-NHC]$^{+}$, 301.4[M-AgCl]$^{+}$; (ESI-): 266.7 [Br-Ag-Br]$^{-}$

Reference:

1. Sheldrick, G. M. Crystal structure refinement with SHELXL. *Acta Cryst*. **2015**, C71, 3–8. DOI: 10.1107/S2053229614024218.
